# Peptidomic analysis of endogenous plasma peptides from patients with pancreatic neuroendocrine tumours

**DOI:** 10.1002/rcm.8183

**Published:** 2018-07-17

**Authors:** Richard G. Kay, Benjamin G. Challis, Ruth T. Casey, Geoffrey P. Roberts, Claire L. Meek, Frank Reimann, Fiona M. Gribble

**Affiliations:** ^1^ Institute of Metabolic Science Metabolic Research Laboratories Addenbrooke's Hospital, Hills Road Cambridge CB2 0QQ UK; ^2^ Institute of Metabolic Science Wolfson Diabetes and Endocrine Centre Addenbrooke's Hospital Cambridge UK; ^3^ IMED Biotech Unit, Clinical Discovery Unit, AstraZeneca UK

## Abstract

**Rationale:**

Diagnosis of pancreatic neuroendocrine tumours requires the study of patient plasma with multiple immunoassays, using multiple aliquots of plasma. The application of mass spectrometry based techniques could reduce the cost and amount of plasma required for diagnosis.

**Methods:**

Plasma samples from two patients with pancreatic neuroendocrine tumours were extracted using an established acetonitrile‐based plasma peptide enrichment strategy. The circulating peptidome was characterised using nano and high flow rate liquid chromatography/mass spectrometry (LC/MS) analyses. To assess the diagnostic potential of the analytical approach, a large sample batch (68 plasmas) from control subjects, and aliquots from subjects harbouring two different types of pancreatic neuroendocrine tumour (insulinoma and glucagonoma), were analysed using a 10‐min LC/MS peptide screen.

**Results:**

The untargeted plasma peptidomics approach identified peptides derived from the glucagon prohormone, chromogranin A, chromogranin B and other peptide hormones and proteins related to control of peptide secretion. The glucagon prohormone derived peptides that were detected were compared against putative peptides that were identified using multiple antibody pairs against glucagon peptides. Comparison of the plasma samples for relative levels of selected peptides showed clear separation between the glucagonoma and the insulinoma and control samples.

**Conclusions:**

The combination of the organic solvent extraction methodology with high flow rate analysis could potentially be used to aid diagnosis and monitor treatment of patients with functioning pancreatic neuroendocrine tumours. However, significant validation will be required before this approach can be clinically applied.

## 1 INTRODUCTION

The application of mass spectrometry to protein analysis is common place, and routine in academic proteomics core facilities. However, the application of mass spectrometry to the analysis of endogenous plasma peptides is less common. A major focus of endogenous peptide research has arguably been the field of peptide neurotransmitters, and in particular those found in brain.^1^ The plasma peptidome is very poorly investigated, due, in part, to the complexity of the circulating protein repertoire, peptide stability and challenging sample handling requirements.^2^ The main obstruction to both plasma proteomics and peptidomics has been sample preparation, requiring the removal of the high‐abundance proteins from samples prior to analysis. Without removing these highly abundant components, the analyses will be hampered by the liquid chromatography/mass spectrometry (LC/MS) instrument detecting signature peptides from these proteins, at the expense of missing the low‐abundance components. Historically, plasma protein extraction methods have involved selective protein removal via immunoaffinity of albumin, immunoglobulins and the top 12 to 14 most abundant proteins.^3^, ^4^ Although effective, the nature of these extraction devices means that throughput is low, and sample volume is limited to tens of microliters per extraction,^5^ which is not conducive to the analysis of peptides in the low pg/mL range. Therefore, these methods are inappropriate for peptidomics. Other sample handling procedures (for both human and rodent plasma) have used size‐exclusion‐based extraction techniques, such as ultrafiltration spin devices.^6^, ^7^ Some plasma peptidomic studies have employed solid‐phase extraction (SPE), whereby plasma is extracted by SPE followed by high‐performance liquid chromatography (HPLC) fractionation and subsequent enzymatic digestion prior to LC/MS analysis.^8^

Although these previous methodologies have shown effectiveness at extracting peptides from plasma, they are not considered high throughput, and in the case of immunoaffinity extraction, have to be performed consecutively. A high‐throughput approach for enriching the low molecular weight (LMW) plasma peptidome/proteome is the use of protein precipitation – either through solvent‐ or acid‐based precipitation.^9^, ^10^ The organic solvent approach has demonstrated the capability of detecting peptides in plasma in the low pg/mL range on its own^11^ or with a subsequent SPE phase using selected reaction monitoring (SRM)‐based analyses.^12^, ^13^ These approaches have shown that plasma pre‐treatment combined with highly targeted triple quadrupole analysis is capable of detecting specific peptides at low pg/mL concentrations, although very few untargeted peptidomics analyses have been published. Studies by Albrechtsen and colleagues have demonstrated the ability to detect gut‐derived peptide hormones in the circulation using untargeted approaches, but these protocols involved large sample volumes and extensive method workflows.^8^, ^14^ Whilst this method proved to be effective, it did not detect the peptides intact (protease digestion was performed), therefore complicating data interpretation. A study by Parker et al. used 200 μL of plasma from multiple time‐points that were combined using a tandem mass tag protocol.^10^ Intact peptides from the proglucagon gene were detected in this study but only the glicentin‐related polypeptide (GRPP) was identified after significant sample workup. In some clinical settings it is not feasible or practical to obtain large plasma sample volumes, and analysis methodologies would be required to do more with less starting material.

Pancreatic neuroendocrine tumours (PNETs) are rare neoplasms that account for 1–2% of all pancreatic tumours.^15^ PNETs may be sporadic or part of an inherited tumour syndrome such as multiple endocrine neoplasia type 1 or von Hippel Lindau syndrome. Furthermore, PNETs can be subdivided into non‐functioning or functioning tumours, the latter referring to tumours capable of secreting peptide hormones or biogenic amines into the circulation usually in a dysregulated manner to produce defined tumour syndromes with varying clinical manifestations. For example, pancreatic islet α‐cell tumours that overexpress proglucagon are typically associated with the glucagonoma syndrome, a rare disease characterised by necrolytic migratory erythema, impaired glucose tolerance, thromboembolic complications and psychiatric disturbance.^15^ Currently, diagnosis of a PNET is made through a combination of clinical examination and history as well as radiological, histological and biochemical investigations. The latter are based, primarily, on immunoassays directed towards a limited number of peptide hormone biomarkers. However, these are of limited diagnostic and prognostic value and there is an unmet need for new and innovative methodologies that can measure and identify novel circulating biomarkers that may be of greater clinical utility.^16^

This study describes the application of a well‐characterised and validated organic solvent based peptide extraction method to the analysis of plasma samples derived from patients with functioning PNETs. Plasma from one of these patients was previously studied extensively using immunoassay‐based techniques to characterise circulating peptidic components from the pancreas, revealing high concentrations of proglucagon‐derived peptides (Case study 1).^17^ Plasma from a second patient diagnosed with a metastatic pancreatic glucagonoma, that had not been previously characterised by immunoassay, was also analysed (Case study 2), along with plasma from a subject harbouring a benign insulinoma and plasma samples taken from a cohort of healthy control subjects. Samples were analysed by low‐ and high‐throughput LC/MS methodologies to assess the feasibility of developing these techniques for use in a clinical setting.

## 2 EXPERIMENTAL

### 2.1 Ethical approvals

This study was approved by the local National Health Service Research Ethics Committee and conducted in accordance with the ethical standards of the Helsinki Declaration of 1975.

### 2.2 Case study 1

This patient has been described previously.^17^ Briefly, a 57‐year‐old female presented with a 12‐month history of necrolytic migratory erythema, constipation, early satiety, nausea and vomiting and weight loss. She was also found to have profound hyperinsulinaemic hypoglycaemia. Radiological, histopathological and initial biochemical investigations confirmed a diagnosis of a well‐differentiated glucagon‐secreting pancreatic neuroendocrine tumour (grade 1) with widespread hepatic metastases. Subsequent gel filtration chromatography and analysis with immunoassays specific for proglucagon‐derived peptides revealed elevated plasma concentrations of several peptides including proglucagon, glucagon, GLP‐1 and GLP‐2. A trial of octreotide (100 μg thrice daily), a short acting somatostatin analogue, successfully reduced plasma levels of proglucagon‐derived peptides resulting in marked clinical improvement including abolition of the hypoglycaemia.

### 2.3 Case study 2

A 54‐year‐old male was diagnosed with a metastatic pancreatic neuroendocrine tumour following investigations for weight loss and abdominal pain. Biochemical assessment of commonly measured fasting gut hormones and neuroendocrine tumour markers by immunoassay revealed isolated elevation of glucagon (179 pmol/L (normal range (NR): 0–50)), chromogranin A (223 pmol/L (NR: 0–60)), and chromogranin B (909 pmol/L (NR: 0–150)). He was commenced on a somatostatin analogue (Lanreotide 120 mg every 28 days) and had radiologically stable disease for the subsequent ten years. In 2017, surveillance cross‐sectional imaging revealed disease progression (Figures S1A and S1B, supporting information). In addition, the patient reported symptoms suggestive of hypoglycaemia and hyperinsulism that were confirmed following a 12‐h fast (plasma glucose 2.8 mmol/L; plasma insulin 30 pmol/L). Everolimus therapy was commenced which normalised glucose levels. The patient remains under close clinical, radiological and biochemical surveillance.

### 2.4 Case study 3

A 22‐year‐old female presented with a 2‐year history of symptoms suggestive of hypoglycaemia that were most troublesome on waking or following exercise and were quickly relieved following carbohydrate ingestion. A supervised inpatient fast confirmed hyperinsulinaemia hypoglycaemia (fasting glucose 1.8 mmol/L; fasting insulin 55 pmol/L; fasting proinsulin 112 pmol/L (NR: 0–70)). Endoscopic ultrasound localised a 44 × 40 mm lesion in the pancreatic tail (Figure S2, supporting information) with no distant metastases identified on computed tomography imaging. EUS‐guided fine needle aspiration of the pancreatic lesion confirmed a diagnosis of a well‐differentiated pancreatic neuroendocrine tumour. Immunohistochemical staining was positive for insulin, chromogranin A and synaptophysin. The patient underwent an uncomplicated distal pancreatectomy. Genetic testing for multiple endocrine neoplasia type 1 was negative. Three years following surgery the patient remains disease free with no biochemical evidence of hypoglycaemia. A fasting plasma sample taken prior to surgery was used for the analyses described herewith.

### 2.5 Control subjects (including ethics and informed consent)

Control plasma samples were collected from 28 healthy adult volunteers as part of a larger study examining endocrine physiology after an oral glucose tolerance or liquid meal test (REC 13/EE/0195). A mixture of samples was available from these individuals, where some were in the fasting state and some from 240 min after a 75 g oral glucose tolerance test, and some samples were obtained from individuals from repeat visits. Blood was collected in EDTA plasma tubes, centrifuged for 10 min at 4°C/3500 *g* and plasma aliquoted and snap‐frozen on dry ice within 30 min of phlebotomy. A total of 62 plasma samples were selected for analysis from the 28 individuals.

### 2.6 Plasma peptide extraction for nano‐LC/MS analysis (Case study 1)

Plasma from Case study 1^17^ was thawed on ice and 50 μL of sample was aliquoted into two separate Eppendorf tubes. One sample had 300 μL of 80:20 ACN/H_2_O (v/v) added and the second aliquot had 300 μL of 80:20:0.1 ACN/H_2_O/formic acid (FA) (v/v/v) added. Both samples were vortex mixed and centrifuged at 14,000 *g* at 4°C and the supernatants transferred to Lo bind Eppendorf tubes. The supernatant was evaporated under a stream of oxygen‐free nitrogen heated to 40°C using a Biotage SPE dry (Upsala, Sweden) evaporation system. The residue was reconstituted into 200 μL of 0.1% FA (v/v) and loaded directly onto an Oasis Prime μ‐elution 96‐well plate (Waters, Wilmslow, UK) and slowly extracted on a positive pressure manifold (Waters). The cartridges were washed with 200 μL of 0.1% FA in water and then 5% methanol in water with 1% acetic acid. The peptides were eluted from the cartridge using 2 × 30 μL of 60% methanol in water with 10% acetic acid. The eluant was evaporated and then reconstituted into 50 μL of 0.1% FA in water and a volume of 20 μL was injected onto a nano‐LC/MS system.

### 2.7 Plasma peptide extraction for high‐throughput LC/MS analysis

An almost identical extraction method was performed on the sample from Case 2 (same plasma as described in case study 2); however, a larger volume of plasma was available for testing. Plasma (EDTA) was separated, aliquoted and rapidly frozen on dry ice for storage before extraction. Plasma was thawed on ice and aliquots of 250 μL were precipitated with 1 mL of 80% ACN in water containing 1 ng/mL of bovine insulin as an internal standard (Sigma Aldrich, Poole, UK). A lower ratio of plasma to ACN was used compared to the 50 μL plasma volume extractions so that the mixing is possible in a 2‐mL 96‐well plate. An additional 62 plasma samples from control individuals were extracted in parallel for comparative analysis of identified peptides. Control samples were extracted into a single aliquot whilst the glucagonoma sample was extracted in duplicate as there was sufficient sample available. The sample used for the nano‐LC/MS analysis (Case 1), and an additional aliquot from Case 1 after 4 days of octreotide therapy, were extracted in a single aliquot due to sample volume limitations. Plasma from the subject with insulinoma (Case study 3) was extracted in duplicate as a further comparator. All samples were spun at 3900 *g* for 10 min and the supernatants transferred and evaporated to dryness before the same SPE process was performed as stated above. However, the SPE eluant was not evaporated, but rather diluted by the addition of 75 μL of 0.1% FA in water to reduce the organic content percentage prior to direct injection onto the LC/MS system. A total of 50 μL was injected onto the LC/MS system in a high flow configuration.

### 2.8 Nano‐LC/MS analysis of plasma extracts

Peptide extracts were analysed using a Thermo Fisher Ultimate 3000 nano LC system coupled to a Q Exactive Plus Orbitrap mass spectrometer (ThermoScientific, San Jose, CA, USA). The analysis was performed using nano‐flow‐based separation and positive ion electrospray. The extracts (20 μL) were injected onto a 0.3 × 5 mm peptide trap column (ThermoFisher Scientific) at a flow rate of 30 μL/min and washed for 10 min before switching in line with a 0.075 × 250 mm nano easy column (ThermoFisher Scientific) flowing at 300 nL/min. Both nano and trap column temperatures were set at 45°C during the analysis. The buffers used for nano‐LC separations were (A) 0.1% FA in water (v/v) and (B) 0.1% FA (v/v) in 80:20 ACN/water. Initial starting conditions were 2.5% B (equating to 2% ACN), and held for 15 min. A ramp to 50% B was performed over 90 min, and the column then washed with 90% B for 20 min before returning to starting conditions for 20 min, totalling an entire run time of 130 min. Electrospray analysis was performed using a spray voltage of 1.8 kV, the tune settings for the mass spectrometer used an S‐lens setting of 70 V to target peptides of higher *m/z* values. A full scan range of 400–1600 *m/z* was performed at a resolution of 75,000 before the top 10 ions of each spectrum were selected for MS/MS analysis. Existing ions selected for fragmentation were added to an exclusion list for 30 s.

### 2.9 High flow rate LC/MS and information‐dependent acquisition (IDA)

Extracted plasma from Case 2 was initially analysed using an IDA‐based analysis, to ascertain if a high flow rate analysis could identify similar peptides to those seen in the nano analysis. Sample (50 μL) was injected onto a HSS T3 column (2.1 × 50 mm; Waters) at 7.5% A (0.1% FA in water, v/v) and 92.5% B (0.1% FA in ACN) at a flow rate of 300 μL/min and separated using a 25‐min gradient to 40% B. The column was washed for 2 min at 90% B before returning to initial condition at 27 min for a total run time of 30 min. IDA‐based analysis involved a full scan of *m/z* 500 to 1600 with a resolution of 75,000, AGC of 3e6 and max fill time of 100 ms. The top three ions were selected for MS/MS fragmentation with a resolution of 17,500, an AGC of 5e5 and a max fill time of 200 ms.

### 2.10 High flow rate full scan LC/MS analysis

Extracts (50 μL) from the control cohort were injected onto a HSS T3 column (2.1 × 50 mm; Waters) at 15% A (0.1% FA in water, v/v) and 85% B (0.1% FA in ACN) at a flow rate of 300 μL/min and separated using a 6.5‐min gradient to 40% B. The column was washed for 1.5 min at 90% B before returning to initial conditions at 8 min for a total run time of 10 min. IDA‐based analysis involved a full scan of *m/z* 600 to 1600 with a resolution of 75,000, AGC of 3e6 and max fill time of 200 ms.

### 2.11 Endogenous peptide identification

The nano‐LC/MS files obtained from the two different extracts of Case 1 were combined and searched using Peaks 8.0 software (BSI, Waterloo, Canada) against the human Swissprot database (downloaded on 06 May 2016). A no‐digest setting was used, which enabled peptides of up to 65 amino acids in length to be matched, and precursor and product ion tolerances were set at 10 ppm and 0.05 Da, respectively. The search parameters included variable modifications such as methionine oxidation, N‐terminal pyro‐glutamate, N‐terminal acetylation and C‐terminal amidation. A false discovery rate (FDR) value of 1% was used to filter the results, with a minimum of 1 unique peptide also required. However, the use of a 1% FDR value when using a non‐specific digest approach is potentially questionable, as it could be overly punitive due to much higher chances of matching peptides in the decoy database. Therefore, where peptides from prohormones were identified, additional peptides from that protein with −10LogP values below the 1% FDR rate were included in the search results. The two high flow rate analysis files were searched using the same parameters.

### 2.12 Comparative analysis of endogenous plasma peptides

The 68 LC/MS files from the 10‐min method were interrogated for a subset of peptides that were identified in the nano‐LC/MS analysis of Case 1 (proglucagon‐derived peptides, CHGA, CHGB and SCG2) and for other endogenous peptides (insulin, c‐peptide, insulin‐like growth factors 1 and 2 (IGF‐1 and IGF‐2), intact hepcidin‐25) and the internal standard (bovine insulin). Peptide *m/z* values from the nano‐LC/MS database search were used to identify peptides in the high flow rate data files, and details of peptide *m/z* values and retention times are included in Table 1. The peaks were integrated using the Quan Browser program in Xcalibur version 3.063 (ThermoFisher). The default integration algorithm used was ICIS, with 7 smoothing points, baseline window of 40, area noise factor of 5 and a peak noise factor of 10. Samples were integrated using the automatic functions and peak integration for each peptide was assessed for consistency.

**Table 1:** Peptide *m/z* values and retention times associated with the peptides selected for semi‐quantitative analysis of a large plasma cohort study

Diagnostic plasma peptides				
Peptide	Molecular weight	Charge	*m/z* ions monitored (±0.01)	RT (min)
Glucagon	3480.6	4	871.16, 871.41, 871.66, 871.91	4.96
Proglucagon 72–108	4167.0	6	695.51, 695.67, 695.84, 696.17, 696.01	5.81
CHGA 97–131	3905.7	4	977.45, 977.70, 977.95, 978.20, 978.45	4.17
CHGA 358–390	3768.7	6	629.14, 629.31, 629.48, 629.64, 629.81	2.42
CHGA 413–456	5060.5	6	844.43, 844.60, 844.77, 844.93, 845.10, 845.27	5.46
CHGA 420–456	4229.1	6	705.87, 706.04, 706.20, 706.37, 706.54	5.87
CHGB 600–613	1743.8	2	872.94, 873.44, 873.94	4.72
SCG2 569–610	4982.4	6	831.42, 831.59, 831.76, 831.92, 832.09, 832.26	5.01
Normal plasma peptides				
Peptide	Molecular weight	Charge	*m/z* ions monitored (±0.01)	RT (min)
Insulin	5803.6	5	1161.94, 1162.14, 1162.34, 1162.54, 1162.74	5.36
C‐peptide	3018.5	3	1007.18, 1007.52, 1007.85, 1008.18	5.10
IGF‐1	7644.6	7	1093.52, 1093.67, 1093.81, 1093.95	4.66
IGF‐2	7464.5	7	1067.79, 1067.94, 1068.08, 1068.23	4.36
Hepcidin 25	2787.0	4	697.76, 698.01, 698.26, 698.51	3.47
Bovine insulin	5729.6	5	1147.33, 1147.53, 1147.73, 1147.93	5.21

## 3 RESULTS

### 3.1 Nano‐LC/MS analysis of PNET plasma sample (Case 1)

The nano‐LC/MS analysis of the two extracted plasma samples returned significant numbers of peptide matches (1643) which corresponded to 582 distinct peptides, suggesting that some peptides were selected for fragmentation multiple times. This was due to many peptides having multiple charge states, which were then selected for fragmentation by the mass spectrometer, and therefore generated multiple hits against the same peptide. Furthermore, high‐abundance peptides had significantly broader peaks than other lower‐abundance peptides, and resulted in the same *m/z* precursors being selected for fragmentation after the dynamic exclusion period had elapsed. The 582 distinct peptides corresponded to a total of 119 unique proteins, as significant numbers of peptides were detected from the higher‐abundance plasma proteins. For example, the top two protein hits were fibrinogen alpha‐chain and alpha‐1‐antitrypsin with 49 and 27 peptides, respectively. The identified proteins are presented in Table 1 (supporting information). The search results identified a number of classical plasma peptides, some from the pancreas and others from different tissues in the body.

#### 3.1.1 Proglucagon‐derived peptides

**Figure 1:**
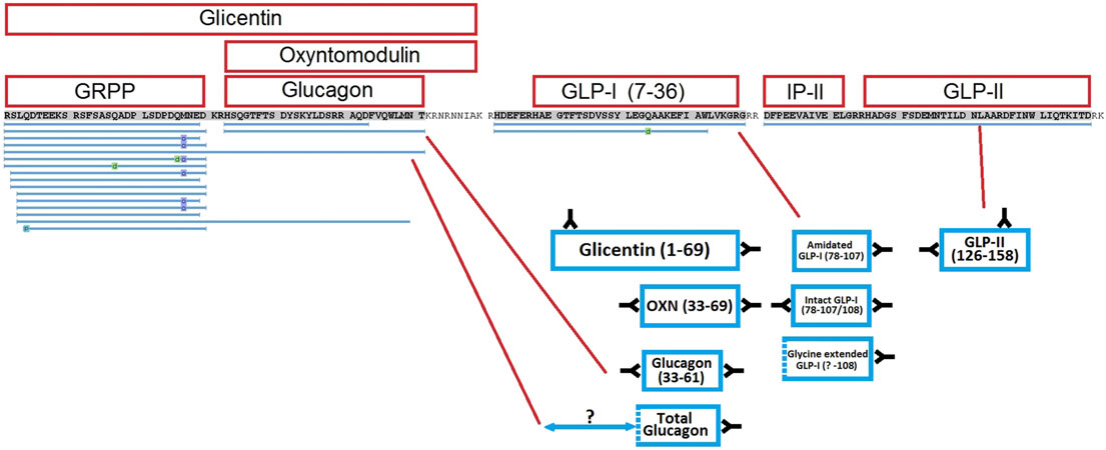
Peptides matched against the glucagon gene using the Peaks software for Case study 1. The matched peptides are compared against the proposed plasma peptide components by Challis et al.,^17^ and showing which peptides identified by LC/MS/MS could potentially have immunoreactivity with the specific antibody reagents [Color figure can be viewed at http://wileyonlinelibrary.com]

Interestingly, multiple peptides derived from proglucagon were detected in the extracts (Table 2, Figure 1). A total of 16 peptides from the proglucagon peptide sequence were detected using the 1% FDR value; however, the Peaks software matched two extra peptides that were below the −10LogP value of 27.2 set by the 1% FDR setting. In total, twelve peptides were detected from the N‐terminal region of proglucagon which contained the GRPP sequence (proglucagon 1–30). These twelve peptides included full‐length GRPP, and GRPP with amino acids missing at both N‐ and C‐terminals, thus matching peptides identified previously by LC/MS to be secreted from human pancreatic islets.^18^ Five of the GRPP peptides were detected with post‐translational modifications such as oxidised methionines, and one had a pyroglutamate N‐terminus after cleavage of the first two amino acids of GRPP. The full‐length GRPP peptide was detected with the highest peak area out of all the proglucagon‐derived peptides, with its breakdown products also having high peak areas. However, without comparing instrument signals for each of the peptides against a synthetically produced standard, relative plasma peptide concentrations of the proglucagon‐derived peptides can only be crudely estimated. The analysis identified the presence of full‐length glucagon (proglucagon 33–61), as well as a degradation product (proglucagon 33–53), that could potentially have been formed by a neprilysin‐type cleavage (N‐terminal side of hydrophobic amino acids). The search also identified peptides that spanned the cleavage site between GRPP and glucagon, based on the long N‐terminal proglucagon (1–61) peptide. Interestingly, glucagon (3–61) was also detected, which could be a DPP4‐cleaved metabolite of proglucagon (1–61). These peptides had low −10LogP values of 5.6 and 6.4, respectively, which may be due to poor peptide fragmentation and their large size making their LC/MS/MS signals lower compared with other shorter peptides in the analysis. The previous study of human islet cell culture supernatants by LC/MS^18^ also identified the presence of proglucagon (1–61), suggesting that this peptide can be produced by pancreatic alpha cells, consistent with its identification in plasma from a patient with a proglucagon‐producing PNET. Furthermore, a recent peptidomic study of human plasma identified this peptide by mass spectrometry, and suggested that it is present in otherwise healthy humans, albeit at low concentrations.^14^

**Table 2:** Peptides identified from the glucagon and chromogranin A genes by nano‐LC/MS/MS analysis from patient 1, showing all peptides matched by Peaks software, and any PTMs that were detected

Proglucagon peptide sequence matches								
Amino acid positions	Peptide sequence	PTM	−10lgP	Mass	*m/z*	z	ppm	Peptide name
1‐30	RSLQDTEEKSRSFSASQADPLSDPDQMNED		83.04	3382.485	846.6284	4	−0.2	GRPP
1‐30	RSLQDTEEKSRSFSASQADPLSDPDQ**M**NED	Oxidation	46.99	3398.481	1133.834	3	0.2	Oxidised GRPP
1‐29	RSLQDTEEKSRSFSASQADPLSDPDQMNE		70.61	3267.459	817.8699	4	−2.4	GRPP 1‐29
1‐29	RSLQDTEEKSRSFSASQADPLSDPDQ**M**NE	Oxidation	68.46	3283.453	821.8708	4	0.3	Oxidised GRPP 1‐29
1‐61	RSLQDTEEKSRSFSASQADPLSDPDQMNEDKRHSQGTFTSDYSKYLDSRRAQDFVQWLMNT		5.62	7129.287	892.1702	8	2.4	Glucagon 1‐61
2‐30	SLQDTEEKSRSFSASQADPLSDPDQMNED		68.27	3226.384	1076.469	3	0	GRPP 2‐30
2‐30	SLQDTEEKSRSFSASQADPLSDPDQ**M**NED	Oxidation	34.33	3242.379	1081.797	3	−3	Oxidised GRPP 2‐30
2‐29	SLQDTEEKSRSFSASQADPLSDPDQMNE		47.49	3111.357	1038.127	3	0.4	GRPP 2‐29
3‐30	LQDTEEKSRSFSASQADPLSDPDQMNED		69.8	3139.352	1047.457	3	−0.7	GRPP 3‐30
3‐30	LQDTEEKSRSFSASQADPLSDPDQ**M**NED	Oxidation	66.04	3155.347	1052.79	3	0.2	Oxidised GRPP 3‐30
3‐29	LQDTEEKSRSFSASQADPLSDPDQMNE		59.21	3024.325	1009.116	3	0.3	GRPP 3‐29
3‐29	LQDTEEKSRSFSASQADPLSDPDQ**M**NE	Oxidation	55.12	3040.32	1014.449	3	1.7	Oxidised GRPP 3‐29
3‐61	LQDTEEKSRSFSASQADPLSDPDQMNEDKRHSQGTFTSDYSKYLDSRRAQDFVQWLMNT		6.41	6886.153	984.7388	7	−4.7	Glucagon 3‐61
4‐30	**Q**DTEEKSRSFSASQADPLSDPDQMNED	Pyro‐glu from Q	52.36	3009.242	1004.091	3	2.8	GRPP 4‐30
33‐53	HSQGTFTSDYSKYLDSRRAQD		64.38	2461.126	616.2883	4	−0.7	Glucagon 1‐21
33‐61	HSQGTFTSDYSKYLDSRRAQDFVQWLMNT		55.81	3480.616	871.1605	4	−0.8	Glucagon
72‐108	HDEFERHAEGTFTSDVSSYLEGQAAKEFIAWLVKGRG		38.99	4166.024	695.3439	6	−1.1	GLP‐I 1‐37
111‐158	RDFPEEVAIVEELGRRHADGSFSDEMNTILDNLAARDFINWLIQTKITD		43.7	5503.699	918.2922	6	1.8	IP‐II and GLP‐II

CHGA peptide sequence matches								
Amino acid positions	Peptide sequence	PTM	−10lgP	Mass	*m/z*	z	ppm	Peptide name
97‐130	HSGFEDELSEVLENQSSQAELKEAVEEPSSKDVM		94.65	3776.721	945.1887	4	1.2	
97‐130	HSGFEDELSEVLENQSSQAELKEAVEEPSSKDV**M**	Oxidation	50.39	3792.716	949.1824	4	−4.1	
97‐131	HSGFEDELSEVLENQSSQAELKEAVEEPSSKDVME		85.6	3905.764	977.4465	4	−1.7	
228‐265	EEEEEEEEEAEAGEEAVPEEEGPTVVLNPHPSLGYKEI		21.65	4221.876	1408.299	3	−0.6	
358‐375	LEGQEEEEDNRDSSMKLS		14.75	2094.901	699.3076	3	0	
358‐376	LEGQEEEEDNRDSSMKLSF		38.49	2241.97	748.3314	3	1.2	LF‐19
358‐390	LEGQEEEEDNRDSSMKLSFRARAYGFRGPGPQL		48.5	3768.791	754.7643	5	−1.7	
413‐456	GYPEEKKEEEGSANRRPEDQELESLSAIEAELEKVAHQLQALR**R**	Amidation	57.99	5060.555	633.5782	8	2.4	GR‐44

Additional identified proglucagon‐derived peptides included proglucagon 72–108 which is also known as GLP‐1_(1–37)_, a proglucagon product without activity at the GLP‐1 receptor,^19^ to which the software initially assigned a C‐terminal amide modification. This modification is believed to have been assigned in error due to the instrument assigning the precursor *m/z* incorrectly, possibly due to the complexity of the plasma extract. Another proglucagon‐derived peptide spanned intermediate peptide 2 (IP‐2) and glucagon‐like peptide 2 (GLP‐2) which corresponds to glucagon (111–158), suggesting that these sequences had not been enzymatically separated. Two major proglucagon‐derived peptides that were not identified in the search were glicentin and oxyntomodulin. Glicentin is a 69 amino acid peptide, and is larger than the 65 amino acid maximum length searched for by the Peaks software using the no‐enzyme digest setting. The theoretical *m/z* values for multiple charge states of glicentin were calculated and the data searched for their presence; however, no peptide could be found that matched its proposed molecular weight. The same process was performed for oxyntomodulin, which has previously been detected in plasma samples using LC/MS approaches by our laboratory (data not shown), but was not found in this case after manually searching the LC/MS datasets.

#### 3.1.2 Pancreatic polypeptide

In Case 1, plasma levels of pancreatic polypeptide (PP) measured by immunoassay were 542 pmol/L, significantly higher than the reference range (NR: 0–300 pmol/L). The LC/MS analysis identified two peptides from the PP prohormone sequence and two additional peptides below the 1% FDR level, one of which corresponded to an N‐terminal extended pancreatic polypeptide that has been detected in previous studies.^18^

#### 3.1.3 Chromogranin A

Chromogranin A (CHGA) is involved in the storage and secretion of regulatory peptides, and is also subject to post‐translational cleavage to form shorter peptides with unclear physiological roles. Chromogranin A is a marker of enteroendocrine cells in tissues and is used clinically as a plasma biomarker of NETs**.** 20 The plasma level of CHGA measured by immunoassay in Case 1 was reported as 146 pmol/L (NR: 0–60 pmol/L). The LC/MS analysis identified a total of nine peptides from the pro‐peptide, three below the 1% FDR value (Table 2), including two previously characterised peptides from CHGA known as LF‐19 and GR‐44. Of the CHGA peptides identified in plasma, all but one were also identified in the previous analysis of peptides secreted from pancreatic islets,^18^ and all peptides were detected in a peptidomics analysis of an islet cell line (QGP‐1).^21^ One detected CHGA peptide was an extended form of LF‐19 with a 14 amino acid C‐terminal extension. Multiple spectra were also obtained from the C‐terminal region of vasostatin‐2, which incorporates residues 97–131 of CHGA. This peptide is not specified in the database as a definitive CHGA peptide, but has been named as vasoconstriction‐inhibiting factor (VIF).^22^ The ability to detect CHGA peptides suggests that LC/MS could potentially be used in the diagnosis and monitoring of NETs.

#### 3.1.4 Secretogranin 1/chromogranin B

The plasma concentration of chromogranin B (CHGB) measured by immunoassay was reported as being higher than the normal range (205 pmol/L, NR: 0–150 pmol/L).^17^ One peptide from this protein was detected above the 1% FDR (residues 90–132), which could potentially be a dipeptidyl‐peptidase cleaved version of peptide 88–132 and was detected in the human islet cell line study.^21^ Looking at lower −10L0gP values, three more peptides from CHGB were detected, one of which has been identified previously in pancreatic islets (residues 600–613).^18^

#### 3.1.5 Glucose‐dependent insulinotropic polypeptide (GIP)

A single peptide was identified from the GIP prohormone (22–51), which corresponds to the first peptide after the signal peptide cleavage position. Swissprot has designated the first peptide in the GIP prohormone sequence as a pro‐peptide, but with a different amino acid sequence (22–50). The peptide detected in plasma has an extra arginine on the C‐terminal. No evidence of mature active GIP (52–93) was detected in this analysis.

#### 3.1.6 Other endocrine peptides from Case study 1

LC/MS analysis of the plasma extracts did not pick up somatostatin, gastrin or vasoactive intestinal peptide (VIP)‐derived peptides, likely because their concentrations were below the sensitivity of the instrument, as their levels measured by immunoassay in this patient were only 74, 10 and 14 pmol/L, respectively.^17^ The peptide YY concentration, according to the previously reported immunoassay data, was 237 pmol/L, which is likely to be in the range of the LC/MS system, since other peptides were identified in the low hundred pmol/L range (e.g. intact glucagon was present at 107 pmol/L).^17^ The reason for PYY not being detected is unknown, but it is possible this peptide may have degraded, or was too low in signal to trigger an MS/MS spectrum.

#### 3.1.7 Hepcidin

Multiple peptide matching sequences from hepcidin were identified; however, as the methodology did not include a reduction and alkylation step, the search result did not identify the 25 amino acid hepcidin. This peptide hormone is produced in the liver and believed to be important in the regulation of iron uptake.^23^ Instead, the peptides that were identified came from the N‐terminal region of the pro‐peptide, and likely represent peptides formed in parallel with hepcidin by furin‐mediated processing.^24^ Hepcidin is a peptide with at most 25 amino acids from the C‐terminal region of the prohormone and contains eight cysteines which form four internal disulphide bonds. These internal bonds confound the PEAKS spectral matching identification software; however, performing reduction and alkylation on plasma samples extracted using the described method results in the identification of hepcidin 25 and some of its catabolites (data not shown).

#### 3.1.8 Thymosin B4

Thymosin B4 is a 4.9‐kDa peptide involved in the organisation of G‐actin fibres within the cytoskeleton of many cells in the body.^25^ The presence of this peptide in plasma could be due to its release from dying cells, or from white blood cells or platelets circulating within the blood plasma. Although its source is unclear, it is present at concentrations high enough to be measured using a high‐throughput LC/MS/MS methodology, should it be of interest to monitor either the peptide itself or its oxidised form.

#### 3.1.9 Neurosecretory protein VGF

Peptides that were detected from VGF were mainly from the N‐terminal region immediately after the signal peptide. A total of eight peptides were identified, four of which were over the 1% FDR setting. This protein is believed to be involved in the secretory process of regulatory peptides^26^ and secreted peptides from VGF were identified in islets.^18^ These peptides have previously been reported in plasma extracts from an exercise study.^10^ None of the peptides that were identified have been classed as bioactive peptides from the VGF pro‐protein.^26^

#### 3.1.10 Secretogranin 2

Secretogranin 2 (SCG2) is a protein involved in neuroendocrine secretory granule processing, and is also believed to be the source of bioactive peptides.^26^ A total of nine peptides were identified above the 1% FDR setting, and four additional peptides were matched. One had a − 10LogP value of 15.77, which corresponded to the peptide manserin (residues 527–566). A potential DPP4‐cleaved manserin peptide was also detected (529–566). The other SCG2 peptides identified were from the C‐terminal region of the protein (569–610), as well as potential DPP4‐cleaved versions. The peptide from the C‐terminal region of SCG2 was previously identified as an islet‐secreted peptide,^18^ further validating its identification in plasma in this study.

#### 3.1.11 Other peptides of interest

A number of interesting peptide matches were also identified in the nano‐LC/MS analysis of Case study 1 that were not related to classical plasma peptides or their related secretory machinery. This list included peptides from acute phase inflammation such as the serum amyloid A proteins and C‐reactive protein. There were a significant number of hits against peptides from the variable regions of kappa and lambda proteins from IgG, which again could point towards an inflammatory state in the patient. These peptides were detected without the use of protease digestion, and were therefore likely to have been present in the circulation at the time of blood sampling.

### 3.2 High flow rate LC/MS and IDA analysis of glucagonoma (Case study 2)

**Figure 2:**
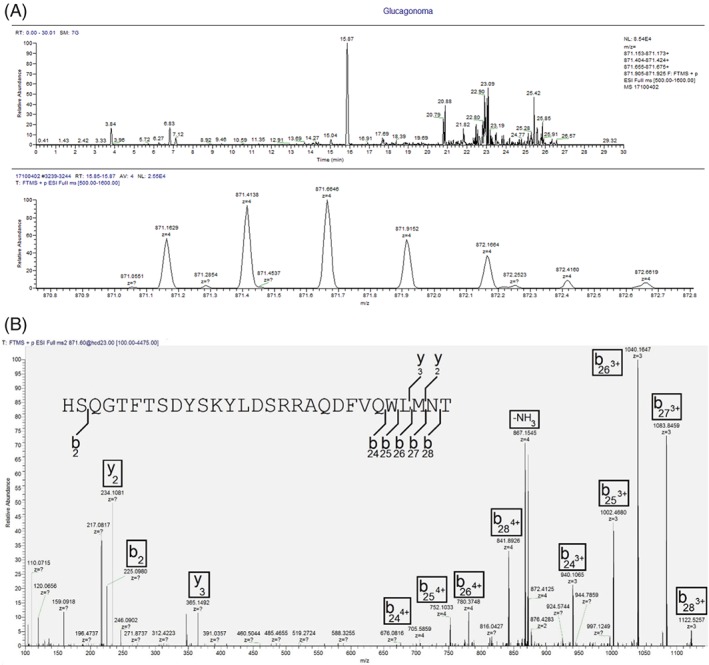
(A) Extracted ion chromatogram for the [M + 4H]^4+^ charge state of glucagon (and the corresponding ^13^C isotope cluster) from the 30‐min analysis of Case study 2. Glucagon has a retention time of 15.87 min. (B) Manual annotation of glucagon MS/MS spectrum, showing multiple b‐ion fragments from the [M + 4H]^4+^ charge state of *m/z* 871.6. The precursor *m/z* of 871.6 was selected to allow more glucagon ^13^C isotopes into the mass spectrometer for fragmentation to boost signal [Color figure can be viewed at http://wileyonlinelibrary.com]

The two aliquots of plasma from Case study 2 were extracted and analysed using a high‐throughput LC/MS and IDA‐based approach, returning significant numbers of peptides for CHGA, which was the top hit. Other proteins that were identified included CHGB, SCG2, neurosecretory protein VGF, PCSK1N and hepcidin (Table S2, supporting information). The majority of peptides that were matched in the high flow rate analysis of plasma from Case study 2 had also been identified in the nano‐LC/MS analysis of Case study 1. A major omission from this analysis was that Peaks did not return a hit for any glucagon gene products, despite the high plasma glucagon levels detected by immunoassay in this patient. Interrogating the raw LC/MS files showed that the system did trigger an MS/MS analysis for the [M + 4H]^4+^ ion of glucagon (*m/z* 871.163) but the spectrum was not of sufficient quality to obtain a positive match. The LC/MS datafile was therefore searched for the presence of glucagon, revealing a peptide that matched the mass of glucagon detected at a retention time of 15.87 min (Figure 2A). This glucagon peptide had a precursor mass of 3481.6279, whilst the expected monoisotopic mass of glucagon is 3481.6230, giving a mass accuracy of 1.4 ppm. A targeted LC/MS/MS analysis of the sample was performed and the product ion spectrum that was obtained for the potential glucagon peptide was manually annotated to demonstrate that the peptide was indeed glucagon (Figure 2B). Additional glucagon gene peptides were similarly investigated (GRPP, proglucagon 72–108 and IP‐2 linked to GLP‐2), and peptides with *m/z* signatures that matched GRPP and proglucagon 72–108 were detected in the high flow rate analysis (data not shown). This indicates that the high flow rate analysis was capable of detecting and confirming the presence of glucagon‐derived peptides and other peptides related to the secretory machinery that are likely co‐secreted with glucagon.

### 3.3 High‐throughput full‐scan peptide screening analysis

**Figure 3:**
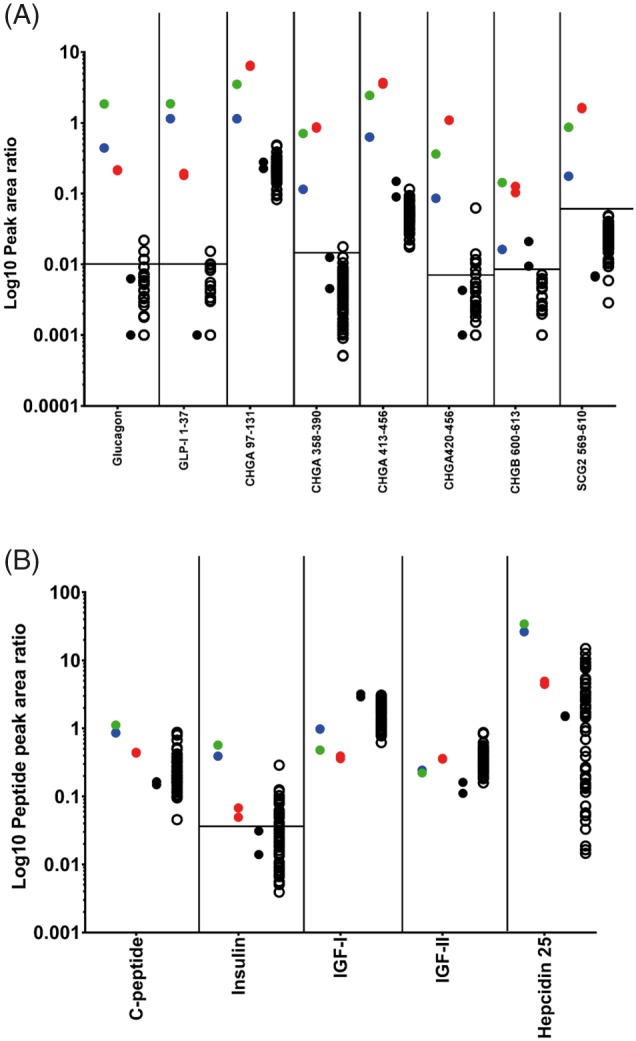
(A) Peak area ratio values of potential diagnostic peptides for glucagonoma – showing glucagon, proglucagon 72–108 and peptides from CHGA, CHGB, SCG2 and VGF. The pretreatment sample from Case study 1 is green, whilst the 4‐day treatment sample is blue, the duplicate extractions from Case study 2 are red, the black filled circles are duplicate extractions of the insulinoma sample, and the open black circles are the remaining 62 control samples. (B) Corresponding peptide peak area ratios for other plasma peptides including insulin, C‐peptide, hepcidin 25, IGF‐I and IGF‐II. In both graphs, the horizontal line for each peptide corresponds to the threshold for peaks having a signal‐to‐noise ratio greater than 3; any data point below this line could be considered as noise [Color figure can be viewed at http://wileyonlinelibrary.com]

The LC/MS data obtained from the two glucagonoma cases (Case 1 pre‐ and post‐treatment, and Case 2), an insulinoma (Case 3) and 62 control samples from healthy individuals were interrogated for glucagon, CHGA, CHGB, SCG2, insulin, c‐peptide, and the internal standard bovine insulin. Example extracted ion chromatograms showing peptides from a control sample and from Case study 2 are shown in Figures S3A–S3D (supporting information). The peak areas of these peptides were quantified using Quan Browser and demonstrated that the extraction method showed good reproducibility, as the peak area for bovine insulin had a percentage coefficient of variance (%CV) of 16.1 over the 68 analyses. The raw peak area of glucagon from the replicate extraction of the glucagonoma sample (Case 2) was similar at 10.4%CV, whilst the glucagon to bovine insulin peak area ratio had a %CV of 1.8. Therefore, all target peptides had their peak areas expressed as a ratio to bovine insulin in subsequent comparisons. This high degree of reproducibility was expected, as the extraction method used for this study is very similar to a well‐characterised glucagon LC/MS method that was used to quantify both glucagon and GLP‐1 in human plasma.^27^ The peak area ratio values of the selected peptides were plotted and peptides that are commonly measured to diagnose glucagonomas displayed clear separation between the values obtained from the glucagonoma sample extracts and the controls, whereas other plasma peptides showed similar levels (Figures 3A and 3B).

The peptide peak area ratios obtained in the high flow rate analysis suggest that this approach could be developed as a diagnostic tool for confirming the presence of a glucagonoma, as the level of glucagon, proglucagon 72–108, CHGA, CHGB and SCG2 peptides were substantially higher than in the controls. Interestingly, analysis of the pre‐ and post‐octreotide treatment samples from Case study 1 showed that treatment with octreotide caused the plasma level of all peptides displayed in Figure 3A to fall after 4 days. Furthermore, inclusion of the insulinoma sample demonstrated that these peptides were high only in the plasma from the two subjects with glucagonoma, but were not elevated in this single case of a tumour deriving from a pancreatic beta‐cell. As CHGA peptides are produced by a variety of endocrine cell types, the combined analysis of peptides derived from proglucagon and CHGA could potentially be used to help distinguish a glucagonoma from other types of neuroendocrine tumour. The levels of insulin, c‐peptide, IGF‐1, IGF‐2 and hepcidin peptides in the glucagonoma samples showed similar levels to the control samples, suggesting that other circulating peptides are within normal ranges, although a proper quantitative assessment would need to be undertaken to adequately assign their concentrations, which is outside the scope of this study, although existing LC/MS methods are available for their analysis.^28^, ^29^ Further validation would be required before this approach can be used as a diagnostic tool; however, our preliminary methodology definitely shows promise. The extraction method could also be improved further, as the recovery of insulin, C‐peptide and glucagon was calculated as 45, 55 and 34%, respectively. As the methodology has been developed as a generic peptide extraction method, further improvements could be made to improve recovery of specific peptides.

## 4 CONCLUSIONS

This plasma peptidomics methodology has demonstrated the ability to detect peptide hormones produced by PNETs as well as peptides from other regulatory prohormones such as GIP and insulin. These peptides were initially identified from 100 μL of plasma using two different extraction solvents. A nano‐LC/MS/MS analysis of the plasma sample from Case study 1 identified a significant number of proglucagon‐derived peptides, corresponding with levels measured previously by immunoassay. Antibody‐based methods, however, are only capable of generating total concentrations of all the peptide species against which the antibody combinations are immunoreactive, whereas the LC/MS approach indicates precisely which peptides are present. For functioning NETs this is particularly relevant as pro‐peptide processing may be incomplete, and the partially processed prohormones may exhibit mixed reactivity against different immunoassays depending on the exact antibody combinations used. The LC/MS system identified a large glucagon peptide (1–61) and its degraded form, which was hypothesised in the previous report to be the cause of a large concentration of “total” glucagon defined using a C‐terminal specific antibody.^17^ By LC/MS, there was no evidence of glicentin or oxyntomodulin in these samples, despite our having detected these peptides in other analyses using the same extraction technique. The production of species containing the C‐terminus of glucagon rather than oxyntomodulin is consistent with the tumour being derived from pancreatic alpha cells rather than the gut, as prohormone convertase 2 is responsible for proglucagon cleavage at the C‐terminus of the glucagon sequence,^30^ and is expressed in normal alpha‐cells but not intestinal L‐cells.

This methodology could potentially be used to study and diagnose other NETs that produce a range of prohormones and their cleavage products at supra‐physiological levels. Given the marked heterogeneity that exists amongst neuroendocrine tumours, identifying novel biomarkers or combinations of biomarkers that enable patient stratification may have prognostic or therapeutic utility. Developing innovate methodologies, such as the one we describe, may provide such an opportunity. In the current study, however, it is notable that the cases of metastatic glucagonoma we describe both had significant disease burden and it remains to be determined how well our methodology will perform in less advanced disease. Significant further validation is required before the methodology would be applicable in a clinical setting, such as developing a quantitative method for glucagon‐gene‐derived peptides and the other secretogranin peptides, which was outside the scope of this study. Multiple synthetic peptides, and their equivalent stable isotope labelled internal standards, would need to be synthesised to develop and validate a quantitative methodology. Validation would also involve the analysis of larger patient sample numbers from this rare disease to demonstrate the selectivity and specificity of a mass spectrometric assay compared to the existing immunoassay methodology. However, the described organic solvent precipitation method followed by SPE has demonstrated its capability of enriching for multiple peptide species, therefore making it an ideal pre‐analytical technique. Eventually, a multiplexed and high‐throughput method that targets multiple prohormone‐derived peptides and vesicle‐associated proteins could be developed to diagnose NETs and, importantly, monitor response to treatment more effectively than current radiology‐based surveillance protocols. This would enable a single LC/MS analysis to be used rather than multiple immunoassays for each target peptide/protein. It would not only make the diagnosis pathway simpler, cheaper and require less plasma, but would also indicate the exact peptide sequences produced rather than relying on their varying cross‐reactivity against a panel of antibodies.

## DATA AVAILABILITY

The mass spectrometry peptidomics data have been deposited to the ProteomeXchange Consortium via the PRIDE partner repository with the dataset identifier PXD008465 (Username reviewer07425@ebi.ac.uk, Password 6LSgqFom). The full scan data from the 68 plasma samples is also included in the same submission.

## Supporting information



Data S1. Supporting information
