# Supplementary material for: Peptidomic analysis of endogenous plasma peptides from patients with pancreatic neuroendocrine tumours
Source: Rapid Commun Mass Spectrom. 2018 Jul 17;32(16):1414–24. doi: 10.1002/rcm.8183 (PMC6099210; doi:10.1002/rcm.8183)

Supplementary Figure 1 A. Radiological investigations from Case study 2 - Computed tomography of the abdomen demonstrating extensive liver metastases and pancreatic tumour (arrowhead).


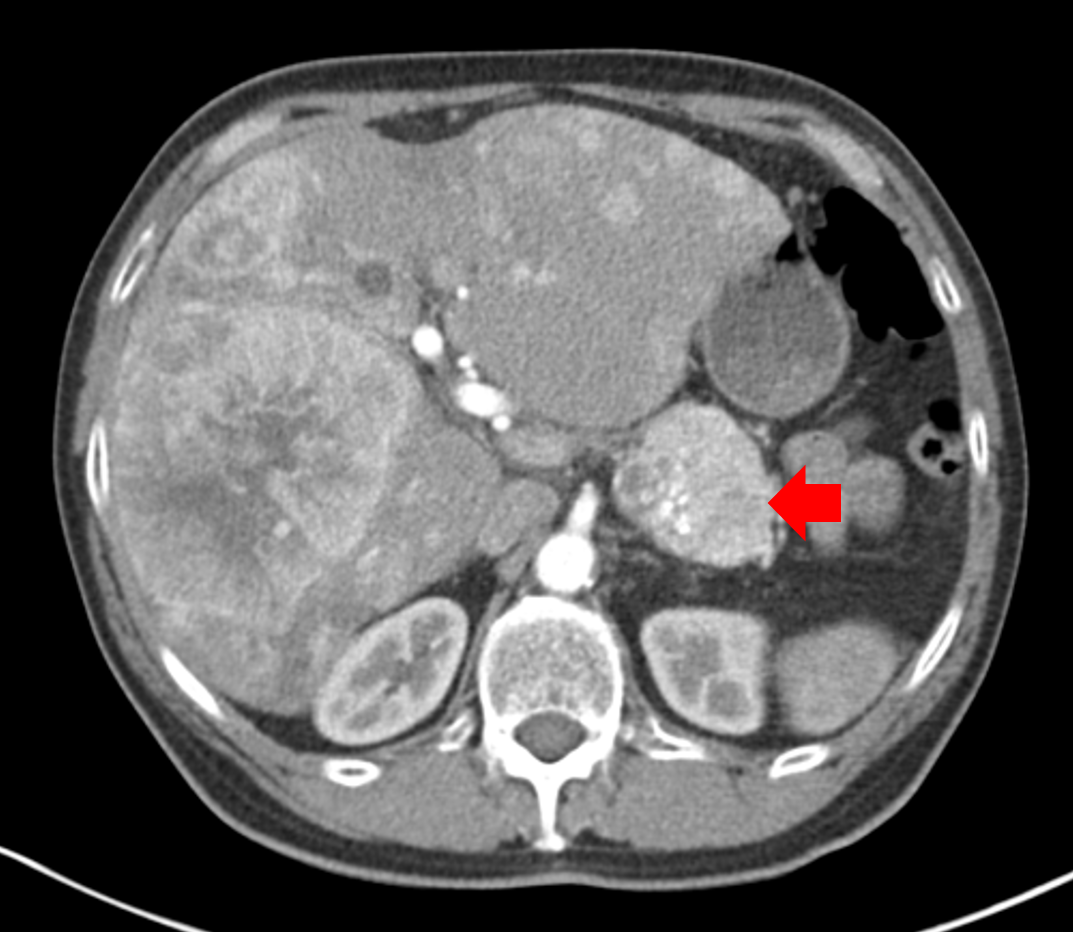


Supplementary Figure 1B. Radiological investigations from Case study 2 - Somatostatin receptor scintigraphy demonstrating hepatic metastases and pancreatic tumour.


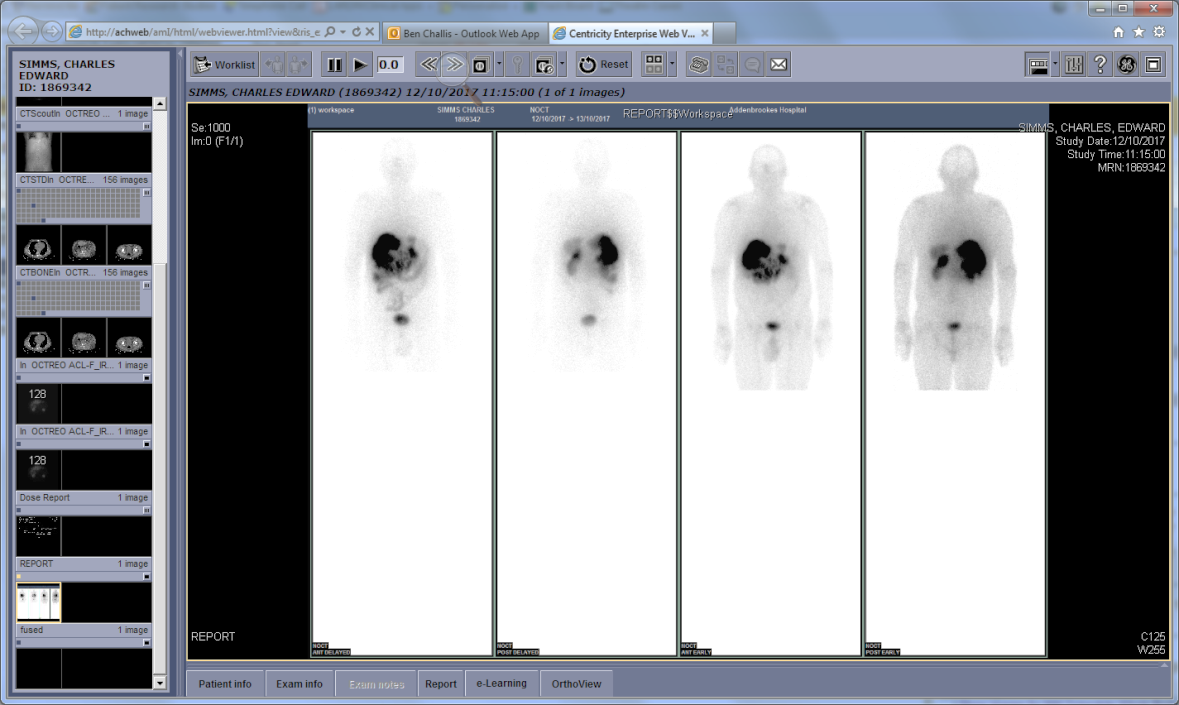


Supplementary figure 2. Representative endoscopic ultrasound image demonstrating the presence of a 44 x 40mm pancreatic insulinoma from case study 3.


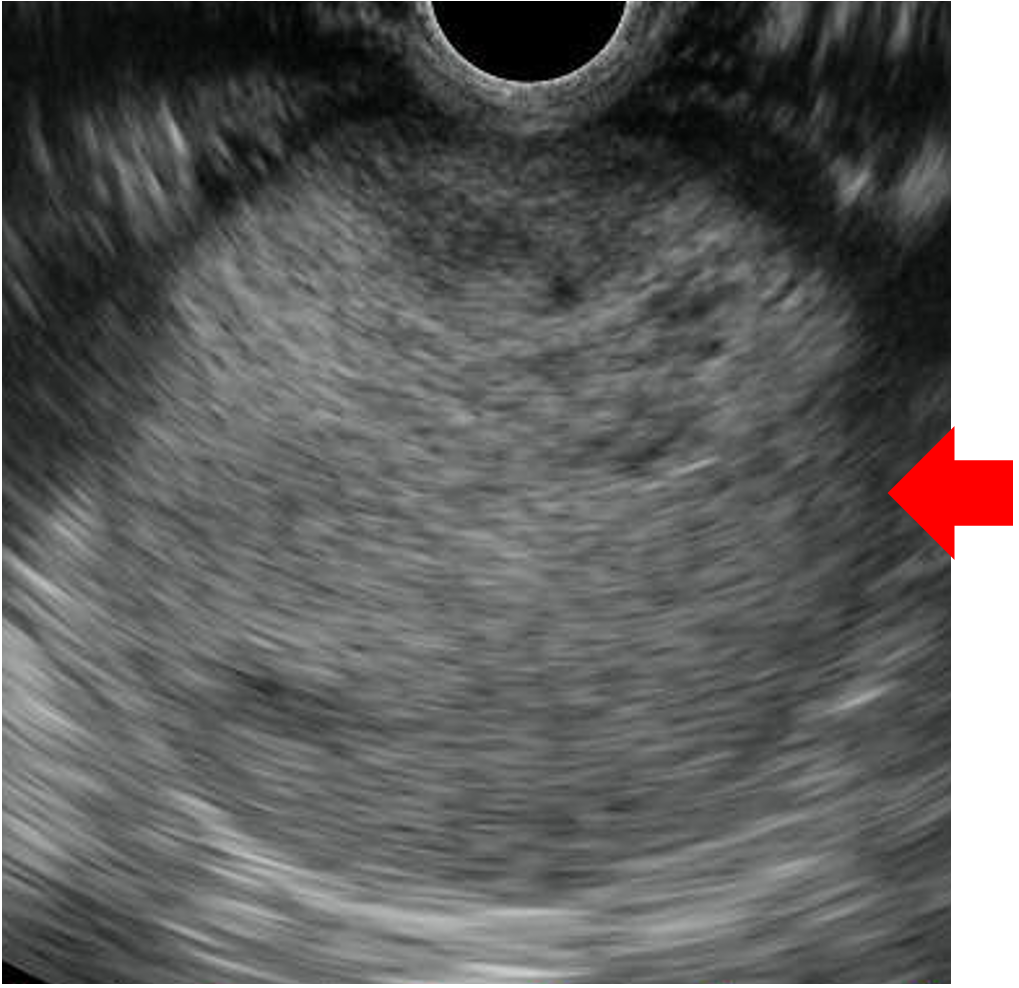


Supplementary Figure 3A and B, showing extracted ion chromatograms of peptides from a control subject plasma extract. Monoisotopic peaks corresponding to the selected peptide (where detected) is labelled with an asterisk. Peptides displayed are outlined in Table 1.

A


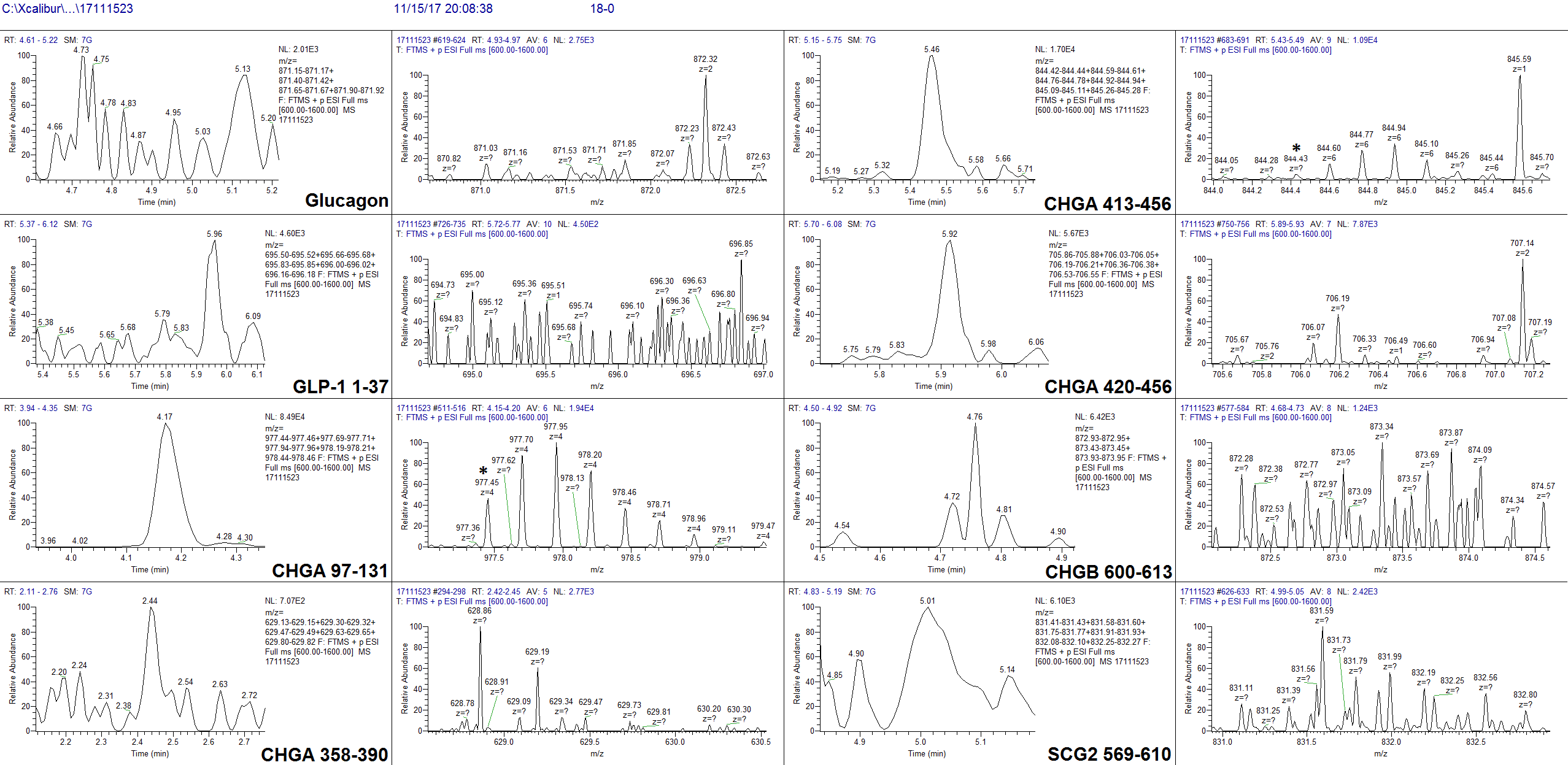


B


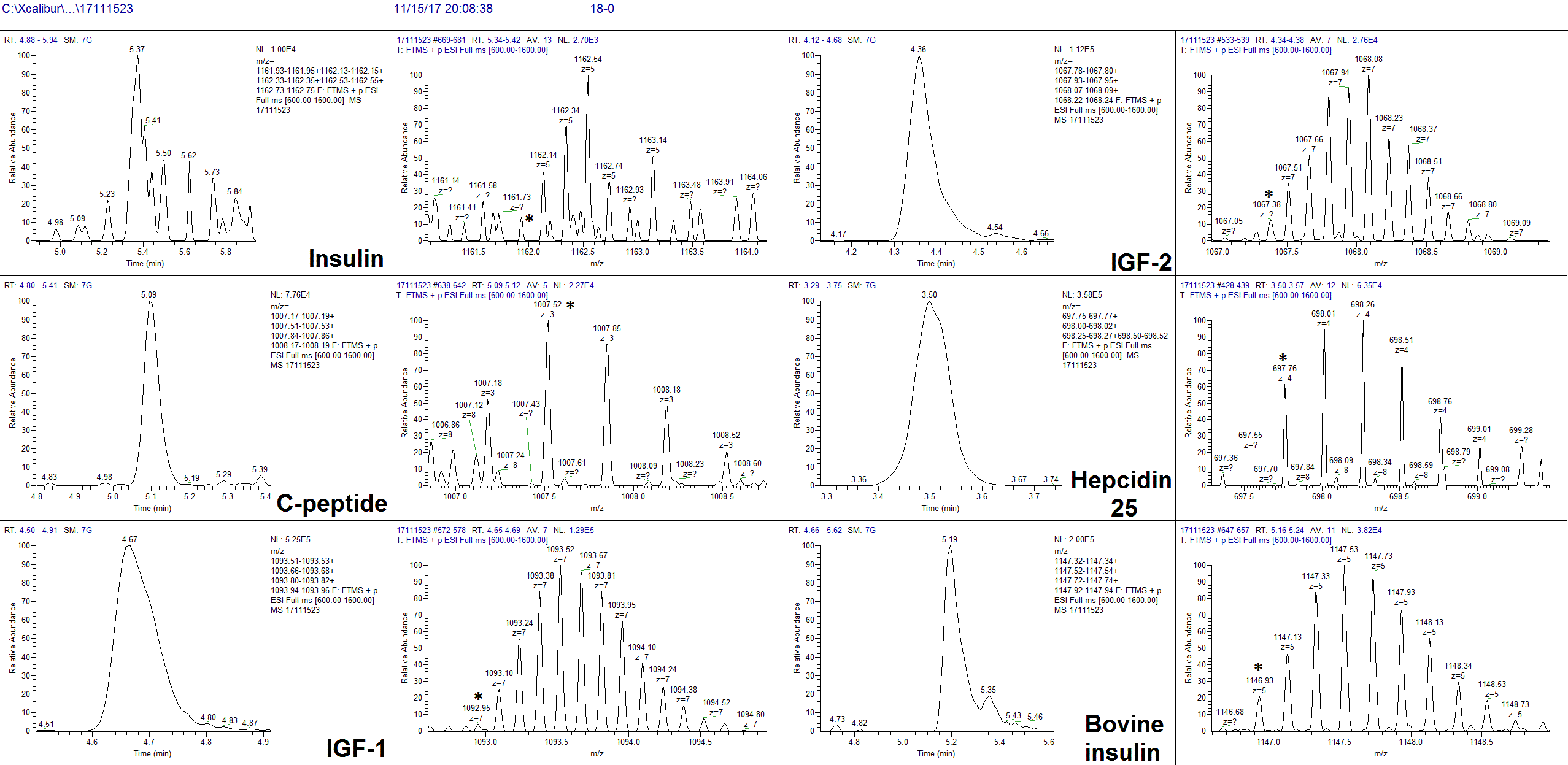


Supplementary Figure 3C and D, showing extracted ion chromatograms of peptides from case study 2 plasma extract. Monoisotopic peaks corresponding to the selected peptide (where detected) is labelled with an asterisk. Peptides displayed are outlined in Table 1.

C


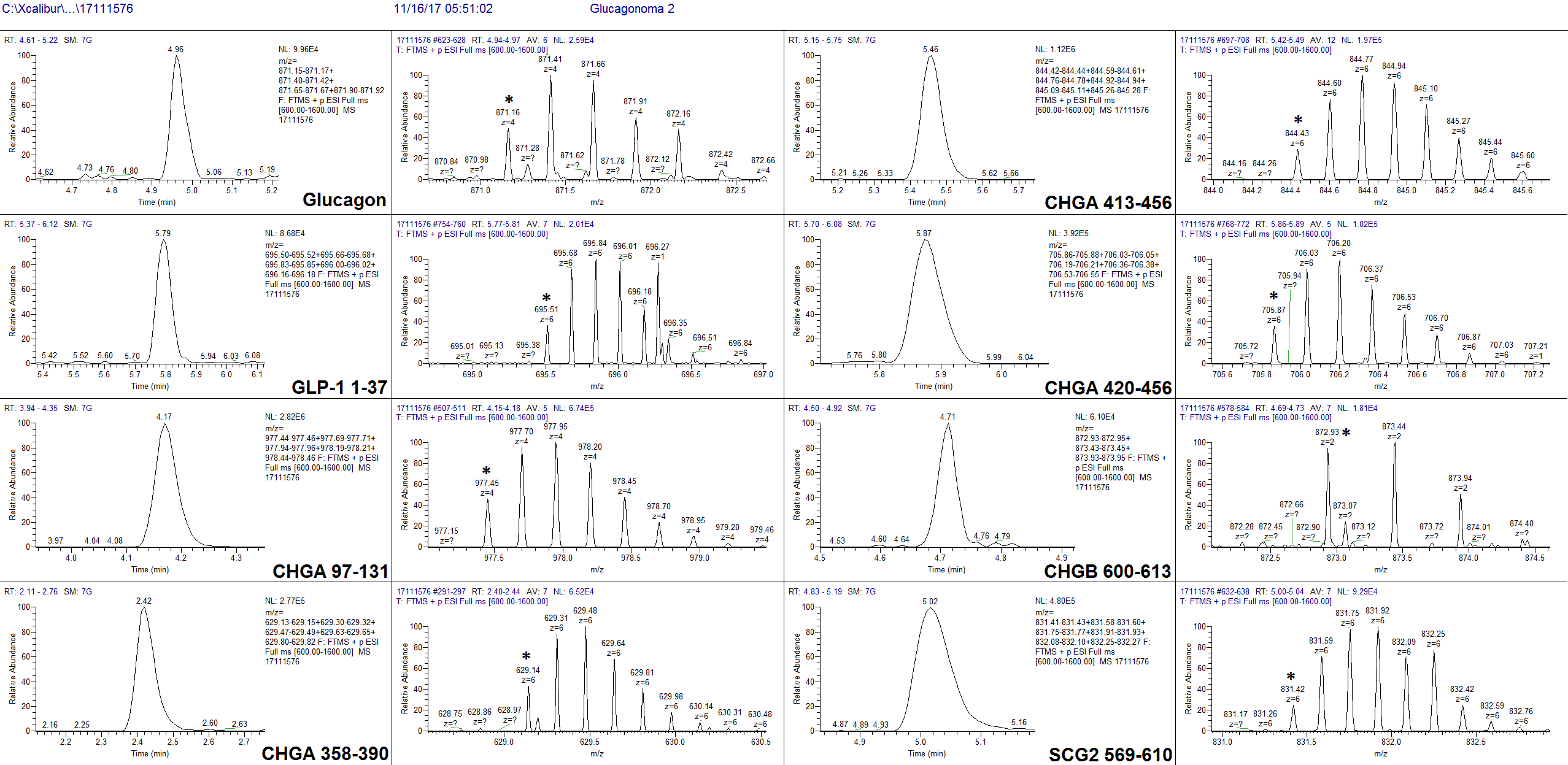


D


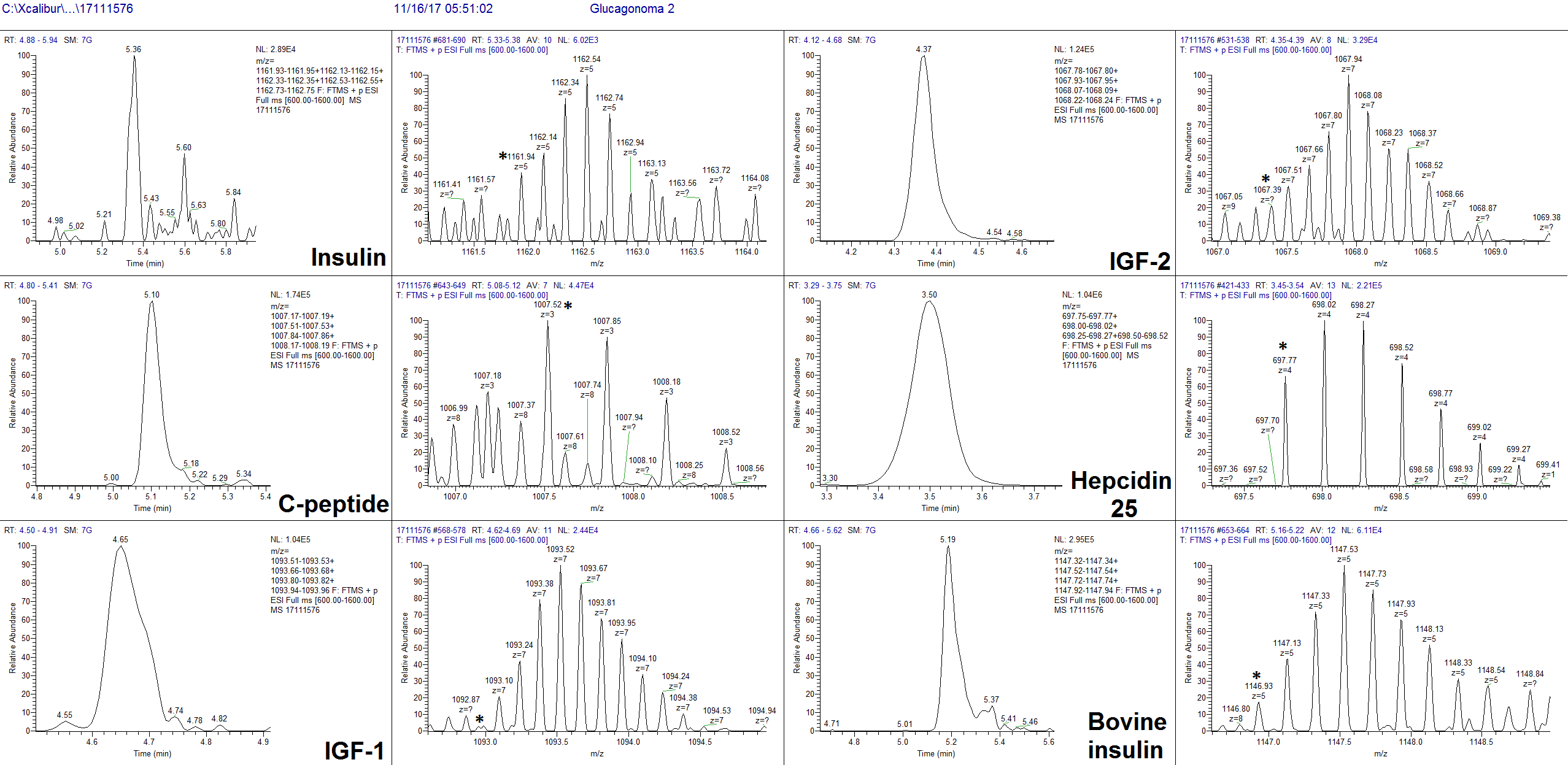

Supplement: Supplementary file 1 — Data S1. Supporting information [file RCM-32-1414-s001.zip › supplementary material_updated.docx]
