# Supplementary material for: Peptidomic analysis of endogenous plasma peptides from patients with pancreatic neuroendocrine tumours
Source: Rapid Commun Mass Spectrom. 2018 Jul 17;32(16):1414–24. doi: 10.1002/rcm.8183 (PMC6099210; doi:10.1002/rcm.8183)
Supplement: Supplementary file 1 — Data S1. Supporting information [file RCM-32-1414-s001.zip › Supplementary table 1 new.pdf]

Protein Gr	Protein ID	Protein Accession	Peptide	Unique	-10lgP	Mass	Length	ppm	m/z	z	RT	Area	Fraction	Scan	Source File	#Spec	Start	End	PTM	AScore
1	1	P02671|FIBA_HUMAN	K.SSSYSKQFTSSTSYNRGDSTFESKS.Y	Y	89.01	2767.221	25	0.8	692.813	4	31.84	7.08E+05	6	5625	17041104.	3	576	600		
1	1	P02671|FIBA_HUMAN	K.SSSYSKQFTSSTSYNRGDSTFESKSY.K	Y	87.29	2930.284	26	-1	733.5776	4	34.8	4.53E+06	6	6165	17041104.	4	576	601		
1	1	P02671|FIBA_HUMAN	R.HRHPDEAAFFDTASTGKTFPGFFSPMLGEFVSETESRGSESGIFTNTKESSSHHPGIAEFPSRG.K	Y	83.62	6929.211	64	0.8	867.1594	8	58.48	6.29E+06	6	10729	17041104.	6	511	574		
1	1	P02671|FIBA_HUMAN	K.SSSYSKQFTSSTSYNRGDSTFESKSYKM.A	Y	77.4	3189.42	28	0	638.8912	5	35.12	8.23E+05	6	6223	17041104.	2	576	603		
1	1	P02671|FIBA_HUMAN	T.ADSGEGDFLAEGGGVR.G	Y	76.96	1535.685	16	-0.6	768.8494	2	41.08		6	7345	17041104.	75	20	35		
1	1	P02671|FIBA_HUMAN	A.DSGEGDFLAEGGGVR.G	Y	75.77	1464.648	15	-0.4	733.331	2	44.06		6	7907	17041104.	60	21	35		
1	1	P02671|FIBA_HUMAN	A.DSGEGDFLAEGGGV.R	Y	73.74	1308.547	14	0.6	655.2811	2	52.42	2.99E+07	6	9618	17041104.	5	21	34		
1	1	P02671|FIBA_HUMAN	K.DSHSLTTNIMEILRGDFSSANNR.D	Y	73.33	2577.224	23	2.7	645.3151	4	62.31	8.03E+05	6	11447	17041104.	1	101	123		
1	1	P02671|FIBA_HUMAN	D.SGEGDFLAEGGGVR.G	Y	70.7	1349.621	14	0.3	675.818	2	38.38	6.64E+07	6	6826	17041104.	9	22	35		
1	1	P02671|FIBA_HUMAN	K.SSSYSKQFTSSTSYNRGDSTFESKSYKMA.D	Y	70.55	3260.457	29	0.3	653.0988	5	35.16	1.51E+06	6	6230	17041104.	4	576	604		
1	1	P02671|FIBA_HUMAN	T.ADSGEGDFLAEGGGV.R	Y	70.39	1379.584	15	0.4	690.7996	2	49.81	7.83E+07	6	9121	17041104.	8	20	34		
1	1	P02671|FIBA_HUMAN	A.D(+42.01)SGEGDFLAEGGGVR.G	Y	67.09	1506.659	15	0.1	754.3366	2	48.39	1.14E+06	5	8750	17041103.	4	21	35	Acetylatioı	D1:Acetylation (N-term):1000.00
1	1	P02671|FIBA_HUMAN	T.A(+42.01)DSGEGDFLAEGGGVR.G	Y	66.16	1577.696	16	-0.6	789.8547	2	49.66	1.72E+06	5	8984	17041103.	2	20	35	Acetylatioı	A1:Acetylation (N-term):1000.00
1	1	P02671|FIBA_HUMAN	K.SQLQKVPPEWKALTDMPQMRME.L	Y	65.66	2642.302	22	3	661.5847	4	51.09	3.48E+05	5	9252	17041103.	1	239	260		
1	1	P02671|FIBA_HUMAN	S.GEGDFLAEGGGVR.G	Y	65.25	1262.589	13	0.2	632.3019	2	38.66	8.74E+07	6	6880	17041104.	7	23	35		
1	1	P02671|FIBA_HUMAN	P.GSTGNRNPGSSGTGGTATWKPGSSGP.G	Y	64.42	2374.09	26	0.3	792.3708	3	27.87	2.10E+05	5	4917	17041103.	1	303	328		
1	1	P02671|FIBA_HUMAN	G.EGDFLAEGGGVR.G	Y	63.83	1205.568	12	-0.1	603.791	2	36.84	1.80E+07	6	6539	17041104.	7	24	35		
1	1	P02671|FIBA_HUMAN	K.SSSYSKQFTSSTSYNRGDSTFESKSYKM(+15.99)A.D	Y	60.38	3276.452	29	-0.8	820.1196	4	32.34	3.71E+05	6	5717	17041104.	2	576	604	Oxidation	M28:Oxidation (M):1000.00
1	1	P02671|FIBA_HUMAN	K.SSSYSKQFTSSTSYNRGDSTFES.K	Y	60.14	2552.094	23	3.1	851.7079	3	34.99	3.19E+06	6	6200	17041104.	1	576	598		
1	1	P02671|FIBA_HUMAN	T.ADSGEGDFLAEGGGVRGPRVV.E	Y	57.32	2043.997	21	0.8	682.3403	3	43.24	9.70E+05	5	7794	17041103.	1	20	40		
1	1	P02671|FIBA_HUMAN	S.Q(-17.03)LQKVPPEWK.A	Y	57.25	1234.671	10	0.7	618.3432	2	40.96	5.89E+05	6	7323	17041104.	3	240	249	Pyro-glu fr	Q1:Pyro-glu from Q:1000.00
1	1	P02671|FIBA_HUMAN	K.SSSYSKQFTSSTSYNRGDSTFESKSYKM(+15.99).A	Y	56.61	3205.415	28	-1.4	642.0893	5	32.1	2.34E+05	6	5673	17041104.	2	576	603	Oxidation	M28:Oxidation (M):1000.00
1	1	P02671|FIBA_HUMAN	P.VPDLVPGNFK.S	Y	56.24	1084.592	10	0.2	543.3032	2	43.71	1.58E+05	6	7844	17041104.	1	229	238		
1	1	P02671|FIBA_HUMAN	G.DFLAEGGGVR.G	Y	51.72	1019.504	10	0.3	510.7592	2	37.03	2.18E+06	6	6572	17041104.	5	26	35		
1	1	P02671|FIBA_HUMAN	T.ADSGEGDFLAEGGGVRGPR.V	Y	51.11	1845.861	19	-0.7	616.2936	3	36.84	3.91E+05	5	6591	17041103.	2	20	38		
1	1	P02671|FIBA_HUMAN	K.TFPGFFSPMLGEFVSETESRGSESGIFTNTKESSSHHPGIAEFPSRG.K	Y	50.44	5061.352	47	-1.6	844.5645	6	61.09	1.97E+06	6	11222	17041104.	1	528	574		
1	1	P02671|FIBA_HUMAN	K.MKPVPDLVPGNFK.S	Y	49.22	1440.78	13	-3.8	481.2654	3	43.16	6.22E+06	5	7779	17041103.	4	226	238		
1	1	P02671|FIBA_HUMAN	K.MKPVPDLVPGNFKSQLQ.K	Y	49.22	1897.013	17	0.3	633.3452	3	46.65	4.16E+06	5	8423	17041103.	2	226	242		
1	1	P02671|FIBA_HUMAN	N.SGSSGPGSTGNRNPGSSGTGGTATWKPGSSGP.G	Y	48.66	2846.282	32	2	949.7697	3	29.42	8.49E+04	6	5181	17041104.	1	297	328		
1	1	P02671|FIBA_HUMAN	K.MKPVPDLVPGNFKSQLQKVPPEWKALTDMPQMRME.L	Y	48.52	4065.071	35	-1.4	814.0203	5	57.67	7.63E+05	5	10486	17041103.	1	226	260		
1	1	P02671|FIBA_HUMAN	V.SETESRGSESGIFTNTKESSSHHPGIAEFPSRG.K	Y	47.95	3504.614	33	1	701.9308	5	33.62	4.10E+05	6	5950	17041104.	1	542	574		
1	1	P02671|FIBA_HUMAN	A.DSGEGDFLAEGGGVRGPRVV.E	Y	47.76	1972.96	20	-0.8	658.6602	3	45.11	7.53E+04	5	8134	17041103.	1	21	40		
1	1	P02671|FIBA_HUMAN	K.DSHSLTTNIMEILRGDFSSANN.R	Y	47.18	2421.123	22	-1.2	808.0473	3	68.43	2.54E+05	5	12495	17041103.	1	101	122		
1	1	P02671|FIBA_HUMAN	T.ADSGEGDFLAEGGGVRGPRVVE.R	Y	46.83	2173.04	22	3.5	725.3564	3	42.61	7.78E+05	5	7676	17041103.	1	20	41		
1	1	P02671|FIBA_HUMAN	K.MKPVPDLVPGNFKSQLQKVPPEWKALT.D	Y	46.12	3046.668	27	0.7	762.6747	4	53.16	1.21E+06	5	9638	17041103.	2	226	252		
1	1	P02671|FIBA_HUMAN	H.SESGSFRPDSPGSGNARPNNPDWGTF.E	Y	45.54	2735.196	26	-0.6	912.7388	3	42.8	2.12E+05	6	7677	17041104.	1	388	413		
1	1	P02671|FIBA_HUMAN	K.M(+15.99)KPVPDLVPGNFK.S	Y	45.12	1456.775	13	0.7	486.5992	3	39.27	1.34E+06	5	7046	17041103.	1	226	238	Oxidation	M1:Oxidation (M):1000.00
1	1	P02671|FIBA_HUMAN	P.VPDLVPGNF.K	Y	44.48	956.4967	9	0.9	479.2561	2	55.97	6.42E+05	6	10268	17041104.	3	229	237		
1	1	P02671|FIBA_HUMAN	E.LERPGGNEITRGGSTSYGTGSETESPRN.P	Y	44.43	2908.355	28	-0.7	728.0955	4	29.45	4.64E+05	5	5213	17041103.	2	261	288		
1	1	P02671|FIBA_HUMAN	P.DLVPGNF.K	Y	43.27	760.3755	7	-2.4	761.381	1	52.67	8.84E+05	6	9664	17041104.	1	231	237		
1	1	P02671|FIBA_HUMAN	K.M(+15.99)KPVPDLVPGNFKSQLQ.K	Y	41.75	1913.008	17	0	638.6766	3	43.94	8.22E+05	5	7922	17041103.	1	226	242	Oxidation	M1:Oxidation (M):1000.00
1	1	P02671|FIBA_HUMAN	K.MKPVPDLVPGNFKSQLQKVPPEWKALTDMPQMR.M	Y	41.7	3804.988	33	3.1	635.1739	6	55.73	3.92E+05	5	10120	17041103.	1	226	258		
1	1	P02671|FIBA_HUMAN	T.ADSGEGDFLAEGGGVRGPRVVERHQ.S	Y	41.57	2594.259	25	0.3	649.5721	4	34.65	9.21E+04	6	6138	17041104.	1	20	44		
1	1	P02671|FIBA_HUMAN	K.SQLQKVPPEWKALT.D	Y	40.95	1623.898	14	0.6	542.3071	3	41.38	5.60E+05	5	7441	17041103.	2	239	252		
1	1	P02671|FIBA_HUMAN	S.SSYSKQFTSSTSYNRGDSTFES.K	Y	39.11	2465.062	22	2.7	822.6967	3	34.97	9.42E+04	6	6197	17041104.	1	577	598		
1	1	P02671|FIBA_HUMAN	E.FVSETESRGSESGIFTNTKESSSHHPGIAEFPSRG.K	Y	38.76	3750.751	35	0.7	751.158	5	36.69	4.19E+05	6	6510	17041104.	1	540	574		
1	1	P02671|FIBA_HUMAN	E.LERPGGNEITRGGSTSYGTGSETESPRNPSSAGSWNSGSSGPGSTGNRN.P	Y	38.27	4853.186	49	1.2	971.6455	5	33.84	3.56E+05	5	6035	17041103.	1	261	309		
1	1	P02671|FIBA_HUMAN	K.QFTSSTSYNRGDSTFESKSY.K	Y	38.24	2290.998	20	0.3	764.6735	3	34.44	1.23E+05	6	6100	17041104.	1	582	601		
1	1	P02671|FIBA_HUMAN	V.SGSTGQWHSESGSFRPDSPGSGNARPNNPDWGTF.E	Y	37.76	3575.547	34	-0.9	894.8933	4	42.64	3.02E+05	6	7648	17041104.	1	380	413		
1	1	P02671|FIBA_HUMAN	R.HRHPDEAAFFDTASTGKTFPGFFSPM(+15.99)LGEFVSETESRGSESGIFTNTKESSSHHPGIAEFPSRG.K	Y	36.8	6945.207	64	0.3	772.6971	9	58.29	1.42E+06	6	10694	17041104.	1	511	574	Oxidation	M26:Oxidation (M):1000.00
1	1	P02671|FIBA_HUMAN	K.MKPVPDLVPGNFKSQLQKVPPEWKALTDMPQMRMELE.R	Y	35.56	4307.198	37	2.4	862.4489	5	59.98	2.13E+05	5	10921	17041103.	1	226	262		
1	1	P02671|FIBA_HUMAN	F.EEVSGNVSPGTRREYHTEKLVTS.K	Y	33.09	2574.267	23	0.4	644.5743	4	28.44	1.16E+05	6	5003	17041104.	1	414	436		
1	1	P02671|FIBA_HUMAN	K.M(+15.99)KPVPDLVPGNFKSQLQKVPPEWKALT.D	Y	31.89	3062.663	27	-1.6	766.6717	4	51.68	2.41E+05	5	9364	17041103.	1	226	252	Oxidation	M1:Oxidation (M):1000.00
1	1	P02671|FIBA_HUMAN	E.LERPGGNEITRGGSTSYGTGSETESPRNPSSAGSWN.S	Y	31.34	3694.684	36	0.1	924.6785	4	35.26	2.20E+05	5	6299	17041103.	1	261	296		
1	1	P02671|FIBA_HUMAN	F.FDTASTGKTFPG.F	Y	30.61	1227.577	12	-0.3	614.7957	2	34.47	1.35E+05	6	6105	17041104.	1	520	531		
1	1	P02671|FIBA_HUMAN	K.SSSYSKQFTSSTSYNRGDSTFESKSYKMADEAGSEADHEGTHSTKRGHA.K	Y	29.21	5333.346	49	-1.3	667.6747	8	33.71	9.84E+05	6	5967	17041104.	1	576	624		
1	1	P02671|FIBA_HUMAN	K.MKPVPDLVPGNF.K	Y	29	1312.685	12	1.4	657.3506	2	51.85	8.77E+04	5	9395	17041103.	1	226	237		
2	13	P01009|A1AT_HUMAN	G.DAAQKTDTSHHDQDHPTFNKITPNLAEFAFSLYRQLAHQ.S	Y	95.37	4492.158	39	1.2	749.7012	6	62.44	9.24E+06	6	11473	17041104.	8	30	68		
2	13	P01009|A1AT_HUMAN	L.MIEQNTKSPLFMGKVVNPTQK	Y	93.47	2389.25	21	0.3	797.4241	3	39.26	5.35E+06	5	7044	17041103.	6	398	418		
2	13	P01009|A1AT_HUMAN	L.M(+15.99)IEQNTKSPLFMGKVVNPTQK	Y	88.65	2405.245	21	-0.2	802.7553	3	37.34	1.62E+06	5	6682	17041103.	3	398	418	Oxidation	M1:Oxidation (M):215.95
2	13	P01009|A1AT_HUMAN	E.AIPMSIPPEVKFNKPFVFLMIEQNTKSPLFMGKVVNPTQK	Y	86.65	4544.44	40	0.8	758.4146	6	63.43	3.45E+07	5	11563	17041103.	9	379	418		
2	13	P01009|A1AT_HUMAN	S.IPPEVKFNKPFVFLMIEQNTKSPLFMGKVVNPTQK	Y	83.22	4045.194	35	1.2	675.207	6	58.97	9.78E+06	5	10729	17041103.	9	384	418		
2	13	P01009|A1AT_HUMAN	A.AGAMFLEAIPMSIPPEVKFNKPFVFLMIEQNTKSPLFMGKVVNPTQK	Y	82.71	5263.772	47	1.1	878.3035	6	68.83	1.11E+06	6	13004	17041104.	4	372	418		
2	13	P01009|A1AT_HUMAN	M.SIPPEVKFNKPFVFLMIEQNTKSPLFMGKVVNPTQK	Y	82.52	4132.226	36	2.3	689.7131	6	58.97	3.29E+06	6	10821	17041104.	8	383	418		
2	13	P01009|A1AT_HUMAN	F.LMIEQNTKSPLFMGKVVNPTQK	Y	81.17	2502.334	22	1	835.1194	3	43.99	2.60E+06	5	7932	17041103.	5	397	418		
2	13	P01009|A1AT_HUMAN	E.AIPM(+15.99)SIPPEVKFNKPFVFLMIEQNTKSPLFMGKVVNPTQK	Y	80.44	4560.435	40	0.2	761.08	6	60.53	1.52E+07	5	11023	17041103.	3	379	418	Oxidation	M4:Oxidation (M):106.42
2	13	P01009|A1AT_HUMAN	F.LM(+15.99)IEQNTKSPLFMGKVVNPTQK	Y	80.03	2518.329	22	1	630.5901	4	42.16	9.02E+06	5	7593	17041103.	4	397	418	Oxidation	M2:Oxidation (M):187.15
2	13	P01009|A1AT_HUMAN	K.FNKPFVFLMIEQNTKSPLFMGKVVNPTQK	Y	79.1	3381.798	29	-2.1	677.3655	5	58.64	1.18E+06	5	10666	17041103.	2	390	418		
2	13	P01009|A1AT_HUMAN	F.LEAIPMSIPPEVKFNKPFVFLMIEQNTKSPLFMGKVVNPTQK	Y	76.75	4786.567	42	1	798.7692	6	64.88	3.64E+07	5	11837	17041103.	8	377	418		
2	13	P01009|A1AT_HUMAN	A.EDPQGDAAQKTDTSHHDQDHPTFNKITPNLAEFAFSLYRQLAHQ.S	Y	75.77	5018.361	44	1.1	1004.681	5	62.34	3.13E+07	6	11454	17041104.	12	25	68		
2	13	P01009|A1AT_HUMAN	E.AIPMSIPPEVKFNKPFVFLM(+15.99)IEQNTKSPLFMGKVVNPTQK	Y	75.53	4560.435	40	4.6	761.0833	6	59.97	1.51E+05	5	10918	17041103.	4	379	418	Oxidation	M20:Oxidation (M):75.68
2	13	P01009|A1AT_HUMAN	M.IEQNTKSPLFMGKVVNPTQK	Y	73.89	2258.209	20	2.2	565.5609	4	37.14	1.59E+06	5	6645	17041103.	3	399	418		
2	13	P01009|A1AT_HUMAN	A.MFLEAIPMSIPPEVKFNKPFVFLMIEQNTKSPLFMGKVVNPTQK	Y	71.42	5064.676	44	1.8	845.1214	6	68.66	1.89E+07	5	12539	17041103.	7	375	418		
2	13	P01009|A1AT_HUMAN	L.MIEQNTKSPLFM(+15.99)GKVVNPTQK	Y	71.31	2405.245	21	0.9	602.319	4	35.79	4.06E+06	5	6397	17041103.	4	398	418	Oxidation	M12:Oxidation (M):122.88
2	13	P01009|A1AT_HUMAN	P.MSIPPEVKFNKPFVFLMIEQNTKSPLFMGKVVNPTQK	Y	69.6	4263.266	37	-4.5	711.5485	6	61.57	1.34E+07	5	11216	17041103.	8	382	418		
2	13	P01009|A1AT_HUMAN	F.LEAIPM(+15.99)SIPPEVKFNKPFVFLMIEQNTKSPLFMGKVVNPTQK	Y	69.41	4802.562	42	-1.1	801.4333	6	62.68	8.60E+06	5	11422	17041103.	4	377	418	Oxidation	M6:Oxidation (M):75.68
2	13	P01009|A1AT_HUMAN	M.FLEAIPMSIPPEVKFNKPFVFLMIEQNTKSPLFMGKVVNPTQK	Y	68.25	4933.635	43	-0.9	823.2791	6	67.04	5.96E+06	5	12237	17041103.	4	376	418		
2	13	P01009|A1AT_HUMAN	G.DAAQKTDTSHHDQDHPTFNKITPNLAEFAFSLYRQLAHQS.N	Y	67.17	4579.19	40	-2.2	764.204	6	61.93	2.60E+06	6	11378	17041104.	5	30	69		
2	13	P01009|A1AT_HUMAN	F.LMIEQNTKSPLFM(+15.99)GKVVNPTQK	Y	66.75	2518.329	22	-0.4	630.5892	4	40.12	2.76E+06	5	7207	17041103.	3	397	418	Oxidation	M13:Oxidation (M):99.02
2	13	P01009|A1AT_HUMAN	G.TEAAGAMFLEAIPMSIPPEVKFNKPFVFLMIEQNTKSPLFMGKVVNPTQK	Y	63.75	5564.898	50	-2.4	928.4881	6	69.96	5.83E+07	6	13277	17041104.	7	369	418		
2	13	P01009|A1AT_HUMAN	S.IPPEVKFNKPFVFLMIEQNTKSPLFM(+15.99)GKVVNPTQK	Y	60.95	4061.189	35	-1.5	677.871	6	57.05	2.06E+06	5	10373	17041103.	2	384	418	Oxidation	M26:Oxidation (M):55.94
2	13	P01009|A1AT_HUMAN	T.EAAGAMFLEAIPMSIPPEVKFNKPFVFLMIEQNTKSPLFMGKVVNPTQK	Y	60.29	5463.851	49	1	1093.779	5	70.28		6	13353	17041104.	2	370	418		
2	13	P01009|A1AT_HUMAN	L.EAIPMSIPPEVKFNKPFVFLMIEQNTKSPLFMGKVVNPTQK	Y	59.4	4673.482	41	1.6	779.9222	6	63.57	4.07E+06	5	11591	17041103.	5	378	418		
2	13	P01009|A1AT_HUMAN	A.EDPQGDAAQKTDTSHHDQDHPTFNKITPNLAEFAFSLYRQLAHQS.N	Y	58.82	5105.393	45	3.1	851.9087	6	62.33	2.24E+07	6	11451	17041104.	8	25	69		
2	13	P01009|A1AT_HUMAN	A.EDPQGDAAQKTDTSHHDQDHPTFNKITPNLAEFAFSLYRQLAH.Q	Y	58.13	4890.302	43	-0.1	816.0576	6	61.49	1.43E+07	6	11297	17041104.	8	25	67		

2	13	P01009|A1AT_HUMAN	E.AAGAMFLEAIPMSIPPEVKFNKPFVFLMIEQNTKSPLFMGKVVNPTQK	Y	57.95	5334.809	48	-2.6	890.1398	6	69.89	4.81E+06	5	12765	17041103.	3	371	418		
2	13	P01009|A1AT_HUMAN	L.M(+15.99)IEQNTKSPLFM(+15.99)GKVVNPTQK	Y	56.88	2421.24	21	0.5	606.3174	4	33.7	2.13E+06	5	6009	17041103.	2	398	418	Oxidation	M1:Oxidation (M):1000.00;M12:Oxidation (M):1000.00
2	13	P01009|A1AT_HUMAN	E.AIPMSIPPEVKFNKPFVFLMIEQNTKSPLFM(+15.99)GKVVNPTQK	Y	56.67	4560.435	40	-0.6	761.0793	6	61.06	1.52E+07	5	11120	17041103.	3	379	418	Oxidation	M31:Oxidation (M):26.12
2	13	P01009|A1AT_HUMAN	G.DAAQKTDTSHHDQDHPTFNKITPNLAEFAFSLYRQLAH.Q	Y	55.32	4364.1	38	-1.4	873.826	5	61.23	7.70E+05	6	11247	17041104.	2	30	67		
2	13	P01009|A1AT_HUMAN	A.GAMFLEAIPMSIPPEVKFNKPFVFLMIEQNTKSPLFMGKVVNPTQK	Y	54.44	5192.734	46	-2.1	866.4612	6	69.46	5.26E+06	5	12687	17041103.	4	373	418		
2	13	P01009|A1AT_HUMAN	A.MFLEAIPM(+15.99)SIPPEVKFNKPFVFLMIEQNTKSPLFMGKVVNPTQK	Y	52.54	5080.67	44	-6.5	847.7802	6	66.16	4.15E+06	5	12075	17041103.	5	375	418	Oxidation	M8:Oxidation (M):61.40
2	13	P01009|A1AT_HUMAN	F.LEAIPMSIPPEVKFNKPFVFLM(+15.99)IEQNTKSPLFMGKVVNPTQK	Y	51.34	4802.562	42	5.4	801.4385	6	61.55	8.60E+06	5	11212	17041103.	2	377	418	Oxidation	M22:Oxidation (M):23.99
2	13	P01009|A1AT_HUMAN	G.TEAAGAM(+15.99)FLEAIPMSIPPEVKFNKPFVFLMIEQNTKSPLFMGKVVNPTQK	Y	48.87	5580.894	50	-3	931.1534	6	68.92	7.13E+06	5	12588	17041103.	1	369	418	Oxidation	M7:Oxidation (M):0.00
2	13	P01009|A1AT_HUMAN	P.M(+15.99)SIPPEVKFNKPFVFLMIEQNTKSPLFMGKVVNPTQK	Y	47.51	4279.261	37	0.9	714.2181	6	59.15	4.22E+06	5	10764	17041103.	2	382	418	Oxidation	M1:Oxidation (M):52.34
2	13	P01009|A1AT_HUMAN	K.GTEAAGAMFLEAIPMSIPPEVKFNKPFVFLMIEQNTKSPLFMGKVVNPTQK	Y	46.16	5621.92	51	0.2	937.9941	6	70.47	2.07E+06	5	12874	17041103.	1	368	418		
2	13	P01009|A1AT_HUMAN	F.LEAIPMSIPPEVKFNKPFVFLMIEQNTKSPLFM(+15.99)GKVVNPTQK	Y	45.16	4802.562	42	-0.8	801.4335	6	63.78	1.23E+07	5	11630	17041103.	3	377	418	Oxidation	M33:Oxidation (M):20.33
2	13	P01009|A1AT_HUMAN	S.IPPEVKFNKPFVFLM(+15.99)IEQNTKSPLFMGKVVNPTQK	Y	43.26	4061.189	35	1.9	677.8733	6	55.05	1.26E+06	5	9995	17041103.	2	384	418	Oxidation	M15:Oxidation (M):56.41
2	13	P01009|A1AT_HUMAN	G.TEAAGAMFLEAIPMSIPPEVKFNKPFVFLM(+15.99)IEQNTKSPLFMGKVVNPTQK	Y	42.6	5580.894	50	1.1	931.1572	6	69.47	7.13E+06	5	12689	17041103.	3	369	418	Oxidation	M30:Oxidation (M):0.00
2	13	P01009|A1AT_HUMAN	G.AMFLEAIPMSIPPEVKFNKPFVFLMIEQNTKSPLFMGKVVNPTQK	Y	42.28	5135.713	45	0.5	856.9598	6	68.8		5	12565	17041103.	1	374	418		
2	13	P01009|A1AT_HUMAN	A.GAM(+15.99)FLEAIPMSIPPEVKFNKPFVFLMIEQNTKSPLFMGKVVNPTQK	Y	42.04	5208.729	46	1.6	869.1301	6	67.72	2.32E+06	5	12365	17041103.	1	373	418	Oxidation	M3:Oxidation (M):67.12
2	13	P01009|A1AT_HUMAN	A.GAMFLEAIPM(+15.99)SIPPEVKFNKPFVFLMIEQNTKSPLFMGKVVNPTQK	Y	42.04	5208.729	46	4	869.1323	6	67.19	9.15E+04	5	12266	17041103.	1	373	418	Oxidation	M10:Oxidation (M):23.99
2	13	P01009|A1AT_HUMAN	G.TEAAGAMFLEAIPM(+15.99)SIPPEVKFNKPFVFLMIEQNTKSPLFMGKVVNPTQK	Y	41.56	5580.894	50	6.8	1117.194	5	69.15	2.69E+06	5	12630	17041103.	2	369	418	Oxidation	M14:Oxidation (M):0.00
2	13	P01009|A1AT_HUMAN	L.EAIPMSIPPEVKFNKPFVFLM(+15.99)IEQNTKSPLFMGKVVNPTQK	Y	39.19	4689.478	41	-1.1	782.586	6	60.86	1.32E+06	5	11083	17041103.	1	378	418	Oxidation	M21:Oxidation (M):2.25
2	13	P01009|A1AT_HUMAN	E.AIPM(+15.99)SIPPEVKFNKPFVFLMIEQNTKSPLFM(+15.99)GKVVNPTQK	Y	39.13	4576.43	40	0.1	763.7457	6	58.95	2.08E+06	5	10725	17041103.	1	379	418	Oxidation	M4:Oxidation (M):47.94;M31:Oxidation (M):10.23
2	13	P01009|A1AT_HUMAN	F.LEAIPM(+15.99)SIPPEVKFNKPFVFLMIEQNTKSPLFM(+15.99)GKVVNPTQK	Y	38.16	4818.557	42	-1.2	804.0991	6	60.69	2.99E+06	5	11052	17041103.	1	377	418	Oxidation	M6:Oxidation (M):13.91;M33:Oxidation (M):71.87
2	13	P01009|A1AT_HUMAN	A.M(+15.99)FLEAIPMSIPPEVKFNKPFVFLMIEQNTKSPLFMGKVVNPTQK	Y	35.95	5080.67	44	-4.4	847.782	6	67.23	8.08E+06	5	12274	17041103.	2	375	418	Oxidation	M1:Oxidation (M):0.00
2	13	P01009|A1AT_HUMAN	M.FLEAIPMSIPPEVKFNKPFVFLM(+15.99)IEQNTKSPLFMGKVVNPTQK	Y	33.73	4949.63	43	-1.2	825.9446	6	64.06	1.14E+06	5	11682	17041103.	1	376	418	Oxidation	M23:Oxidation (M):28.74
2	13	P01009|A1AT_HUMAN	F.LM(+15.99)IEQNTKSPLFM(+15.99)GKVVNPTQK	Y	33.35	2534.324	22	-1.1	845.7809	3	38.59	6.87E+04	5	6917	17041103.	1	397	418	Oxidation	M2:Oxidation (M):1000.00;M13:Oxidation (M):1000.00
2	13	P01009|A1AT_HUMAN	A.EDPQGDAAQKTDTSHHDQDHPTFNKITPNLAEFAFSLYRQLA.H	Y	32.12	4753.243	42	2.2	793.2162	6	63.21	5.64E+06	6	11620	17041104.	1	25	66		
2	13	P01009|A1AT_HUMAN	A.AGAM(+15.99)FLEAIPM(+15.99)SIPPEVKFNKPFVFLMIEQNTKSPLFMGKVVNPTQK	Y	31.64	5295.761	47	1.1	883.6351	6	65.96		5	12038	17041103.	1	372	418	Oxidation	M4:Oxidation (M):28.90;M11:Oxidation (M):5.61
2	13	P01009|A1AT_HUMAN	K.GTEAAGAMFLEAIPM(+15.99)SIPPEVKFNKPFVFLMIEQNTKSPLFMGKVVNPTQK	Y	31.64	5637.915	51	0.7	940.6604	6	69	6.29E+05	5	12602	17041103.	1	368	418	Oxidation	M15:Oxidation (M):20.76
2	13	P01009|A1AT_HUMAN	D.PQGDAAQKTDTSHHDQDHPTFNKITPNLAEFAFSLYRQLAHQ.S	Y	30.9	4774.291	42	3.3	796.7251	6	61.04		6	11212	17041104.	1	27	68		
2	13	P01009|A1AT_HUMAN	L.EAIPM(+15.99)SIPPEVKFNKPFVFLMIEQNTKSPLFMGKVVNPTQK	Y	30.73	4689.478	41	-1	782.5861	6	61.44	1.36E+06	5	11192	17041103.	1	378	418	Oxidation	M5:Oxidation (M):35.41
2	13	P01009|A1AT_HUMAN	G.TEAAGAM(+15.99)FLEAIPMSIPPEVKFNKPFVFLM(+15.99)IEQNTKSPLFMGKVVNPTQK	Y	28.66	5596.889	50	-2.6	933.8196	6	67.25		5	12277	17041103.	1	369	418	Oxidation	M7:Oxidation (M):50.34;M30:Oxidation (M):0.00
4	9	P0C0L5|CO4B_HUMAN	Q.KPRLLLFSPSVVHLGVPLSVGVQLQDVPRGQVVK.G	Y	91.56	3661.156	34	-0.5	733.238	5	56.37	1.70E+06	5	10243	17041103.	2	20	53		
4	9	P0C0L5|CO4B_HUMAN	Q.KPRLLLFSPSVVHLGVPLSVGVQLQDVPRG.Q	Y	86.07	3206.866	30	1	802.7244	4	58.53	1.20E+06	6	10738	17041104.	4	20	49		
4	9	P0C0L5|CO4B_HUMAN	Q.KPRLLLFSPSVVHLGVPLSVGVQLQDVPRGQVVKGSVFLRNPSRN.N	Y	82.32	4888.803	45	0.3	699.408	7	55.14	5.57E+06	5	10011	17041103.	10	20	64		
4	9	P0C0L5|CO4B_HUMAN	Q.KPRLLLFSPSVVHLGVPLSVGVQLQDVPRGQVVKGSVFLRNPSRNN.V	Y	78.3	5002.846	46	-1.2	715.6987	7	55.36	1.13E+06	6	10157	17041104.	3	20	65		
4	9	P0C0L5|CO4B_HUMAN	Q.KPRLLLFSPSVVHLGVPLS.V	Y	78.19	2058.235	19	0.5	687.0861	3	55.91	8.16E+06	5	10154	17041103.	5	20	38		
4	9	P0C0L5|CO4B_HUMAN	Q.KPRLLLFSPSVVHLGVPLSVGVQLQDVPRGQVVKGSVF.L	Y	78.17	4051.346	38	8	811.283	5	59.7	3.32E+06	5	10866	17041103.	4	20	57		
4	9	P0C0L5|CO4B_HUMAN	Q.KPRLLLFSPSVVHLGVPLSVGVQLQ.D	Y	77.93	2682.595	25	1.2	671.6567	4	61.88	5.23E+06	5	11274	17041103.	3	20	44		
4	9	P0C0L5|CO4B_HUMAN	Q.KPRLLLFSPSVVHLGVPLSVGVQ.L	Y	73.78	2441.452	23	0.9	611.3708	4	59.02	1.68E+07	5	10740	17041103.	5	20	42		
4	9	P0C0L5|CO4B_HUMAN	R.NGFKSHALQLNNRQI.R	Y	71.82	1738.923	15	0.3	580.6483	3	31.49	9.23E+06	6	5559	17041104.	10	1337	1351		
4	9	P0C0L5|CO4B_HUMAN	Q.KPRLLLFSPSVVHLG.V	Y	70.76	1661.998	15	0.5	555.0069	3	48.63	2.61E+06	5	8794	17041103.	2	20	34		
4	9	P0C0L5|CO4B_HUMAN	R.NGFKSHALQLNNRQIR.G	Y	65.43	1895.024	16	-0.1	474.7632	4	27.02	1.30E+06	6	4745	17041104.	3	1337	1352		
4	9	P0C0L5|CO4B_HUMAN	K.DDPDAPLQPVTPLQLFEGRRN.R	Y	63.79	2377.203	21	0.6	793.4086	3	59.82	1.65E+08	6	10979	17041104.	12	1429	1449		
4	9	P0C0L5|CO4B_HUMAN	Q.KPRLLLFSPSVVH.L	Y	62.57	1491.893	13	0.3	498.305	3	42.17	5.42E+06	5	7595	17041103.	3	20	32		
4	9	P0C0L5|CO4B_HUMAN	R.GLEEELQFSLGSKINVK.V	Y	62.07	1890.01	17	0.9	631.0111	3	52.66	1.27E+06	6	9662	17041104.	1	1353	1369		
4	9	P0C0L5|CO4B_HUMAN	D.DPDAPLQPVTP.L	Y	61.43	1148.571	11	0.9	575.2935	2	46.98	8.20E+05	6	8461	17041104.	1	1430	1440		
4	9	P0C0L5|CO4B_HUMAN	K.DDPDAPLQPVTP.L	Y	61.15	1263.598	12	-0.9	632.8058	2	47.6	1.98E+06	6	8601	17041104.	2	1429	1440		
4	9	P0C0L5|CO4B_HUMAN	N.GFKSHALQLNNRQI.R	Y	61.02	1624.88	14	-0.9	542.6334	3	30.67	3.96E+05	6	5409	17041104.	4	1338	1351		
4	9	P0C0L5|CO4B_HUMAN	K.DDPDAPLQPVTPLQ.L	Y	59.68	1504.741	14	0.7	753.3782	2	54.1	6.21E+04	6	9926	17041104.	4	1429	1442		
4	9	P0C0L5|CO4B_HUMAN	Q.KPRLLLFSPSVVHLGVPLSVGVQLQDVPRGQVVKGSVFLR.N	Y	59.47	4320.531	40	-1.3	721.0949	6	58.07		5	10561	17041103.	1	20	59		
4	9	P0C0L5|CO4B_HUMAN	K.DDPDAPLQPVTPLQL.F	Y	58.92	1617.825	15	1	809.9206	2	66.78	3.71E+05	5	12190	17041103.	1	1429	1443		
4	9	P0C0L5|CO4B_HUMAN	R.GLEEELQFSLGSK.I	Y	54.21	1435.72	13	0.3	718.8672	2	52.85	5.70E+05	6	9695	17041104.	1	1353	1365		
4	9	P0C0L5|CO4B_HUMAN	R.TLEIPGNSDPNMIPDGDFNSYVRVT.A	Y	53.85	2750.286	25	4	1376.156	2	60.16	2.10E+05	5	10954	17041103.	3	957	981		
4	9	P0C0L5|CO4B_HUMAN	L.LFSPSVVHLGVPLSVGVQ.L	Y	53.73	1834.035	18	-2.2	918.0229	2	61.5	3.34E+05	5	11203	17041103.	1	25	42		
4	9	P0C0L5|CO4B_HUMAN	R.TLEIPGNSDPNM(+15.99)IPDGDFNSYVRVT.A	Y	53.44	2766.281	25	5.2	923.1056	3	56.97	1.80E+05	5	10357	17041103.	1	957	981	Oxidation	M12:Oxidation (M):1000.00
4	9	P0C0L5|CO4B_HUMAN	Q.LSVSAGSPHPAIARLT.V	Y	49.65	1575.873	16	2.5	526.2997	3	37.03	8.15E+05	5	6625	17041103.	1	446	461		
4	9	P0C0L5|CO4B_HUMAN	T.VAAPPSGGPGFLSIERPDSRPPRVG.D	Y	45.41	2515.329	25	0.5	629.8399	4	42.8	1.39E+07	5	7713	17041103.	2	462	486		
4	9	P0C0L5|CO4B_HUMAN	L.LFSPSVVHLGVPLS.V	Y	44.84	1450.818	14	1.4	726.4175	2	57.53	2.81E+05	5	10461	17041103.	1	25	38		
4	9	P0C0L5|CO4B_HUMAN	D.PDAPLQPVTPLQLFEGRRN.R	Y	41.52	2147.149	19	1.2	716.7244	3	55.84	6.69E+05	5	10140	17041103.	3	1431	1449		
4	9	P0C0L5|CO4B_HUMAN	D.DPDAPLQPVTPLQLFEGRRN.R	Y	40.91	2262.176	20	0.7	755.0663	3	59.31	7.32E+04	5	10793	17041103.	1	1430	1449		
4	9	P0C0L5|CO4B_HUMAN	L.FSPSVVHLGVPLS.V	Y	39.23	1337.734	13	0.5	669.8748	2	52.87	1.66E+05	5	9584	17041103.	1	26	38		
4	9	P0C0L5|CO4B_HUMAN	Y.DELPAKDDPDAPLQPVTPLQLFEGRRN.R	Y	32.62	3030.541	27	1.8	758.6439	4	55.82	1.42E+05	6	10240	17041104.	1	1423	1449		
4	9	P0C0L5|CO4B_HUMAN	L.LFSPSVVHLGVPLSVGVQLQDVPRGQVVK.G	Y	28.73	3053.739	29	-0.1	764.442	4	56.75	6.62E+04	5	10315	17041103.	1	25	53		
4	9	P0C0L5|CO4B_HUMAN	L.LFSPSVVHLG.V	Y	28.31	1054.581	10	0.7	528.2982	2	47.6	5.29E+04	5	8603	17041103.	1	25	34		
4	9	P0C0L5|CO4B_HUMAN	A.APPSGGPGFLSIERPDSRPPRVG.D	Y	27.21	2345.224	23	0.7	587.3137	4	40.79	2.44E+05	5	7333	17041103.	1	464	486		
4	10	P0C0L4|CO4A_HUMAN	Q.KPRLLLFSPSVVHLGVPLSVGVQLQDVPRGQVVK.G	Y	91.56	3661.156	34	-0.5	733.238	5	56.37	1.70E+06	5	10243	17041103.	2	20	53		
4	10	P0C0L4|CO4A_HUMAN	Q.KPRLLLFSPSVVHLGVPLSVGVQLQDVPRG.Q	Y	86.07	3206.866	30	1	802.7244	4	58.53	1.20E+06	6	10738	17041104.	4	20	49		
4	10	P0C0L4|CO4A_HUMAN	Q.KPRLLLFSPSVVHLGVPLSVGVQLQDVPRGQVVKGSVFLRNPSRN.N	Y	82.32	4888.803	45	0.3	699.408	7	55.14	5.57E+06	5	10011	17041103.	10	20	64		
4	10	P0C0L4|CO4A_HUMAN	Q.KPRLLLFSPSVVHLGVPLSVGVQLQDVPRGQVVKGSVFLRNPSRNN.V	Y	78.3	5002.846	46	-1.2	715.6987	7	55.36	1.13E+06	6	10157	17041104.	3	20	65		
4	10	P0C0L4|CO4A_HUMAN	Q.KPRLLLFSPSVVHLGVPLS.V	Y	78.19	2058.235	19	0.5	687.0861	3	55.91	8.16E+06	5	10154	17041103.	5	20	38		
4	10	P0C0L4|CO4A_HUMAN	Q.KPRLLLFSPSVVHLGVPLSVGVQLQDVPRGQVVKGSVF.L	Y	78.17	4051.346	38	8	811.283	5	59.7	3.32E+06	5	10866	17041103.	4	20	57		
4	10	P0C0L4|CO4A_HUMAN	Q.KPRLLLFSPSVVHLGVPLSVGVQLQ.D	Y	77.93	2682.595	25	1.2	671.6567	4	61.88	5.23E+06	5	11274	17041103.	3	20	44		
4	10	P0C0L4|CO4A_HUMAN	Q.KPRLLLFSPSVVHLGVPLSVGVQ.L	Y	73.78	2441.452	23	0.9	611.3708	4	59.02	1.68E+07	5	10740	17041103.	5	20	42		
4	10	P0C0L4|CO4A_HUMAN	R.NGFKSHALQLNNRQI.R	Y	71.82	1738.923	15	0.3	580.6483	3	31.49	9.23E+06	6	5559	17041104.	10	1337	1351		
4	10	P0C0L4|CO4A_HUMAN	Q.KPRLLLFSPSVVHLG.V	Y	70.76	1661.998	15	0.5	555.0069	3	48.63	2.61E+06	5	8794	17041103.	2	20	34		
4	10	P0C0L4|CO4A_HUMAN	R.NGFKSHALQLNNRQIR.G	Y	65.43	1895.024	16	-0.1	474.7632	4	27.02	1.30E+06	6	4745	17041104.	3	1337	1352		
4	10	P0C0L4|CO4A_HUMAN	K.DDPDAPLQPVTPLQLFEGRRN.R	Y	63.79	2377.203	21	0.6	793.4086	3	59.82	1.65E+08	6	10979	17041104.	12	1429	1449		
4	10	P0C0L4|CO4A_HUMAN	Q.KPRLLLFSPSVVH.L	Y	62.57	1491.893	13	0.3	498.305	3	42.17	5.42E+06	5	7595	17041103.	3	20	32		
4	10	P0C0L4|CO4A_HUMAN	R.GLEEELQFSLGSKINVK.V	Y	62.07	1890.01	17	0.9	631.0111	3	52.66	1.27E+06	6	9662	17041104.	1	1353	1369		
4	10	P0C0L4|CO4A_HUMAN	D.DPDAPLQPVTP.L	Y	61.43	1148.571	11	0.9	575.2935	2	46.98	8.20E+05	6	8461	17041104.	1	1430	1440		
4	10	P0C0L4|CO4A_HUMAN	K.DDPDAPLQPVTP.L	Y	61.15	1263.598	12	-0.9	632.8058	2	47.6	1.98E+06	6	8601	17041104.	2	1429	1440		
4	10	P0C0L4|CO4A_HUMAN	N.GFKSHALQLNNRQI.R	Y	61.02	1624.88	14	-0.9	542.6334	3	30.67	3.96E+05	6	5409	17041104.	4	1338	1351		
4	10	P0C0L4|CO4A_HUMAN	K.DDPDAPLQPVTPLQ.L	Y	59.68	1504.741	14	0.7	753.3782	2	54.1	6.21E+04	6	9926	17041104.	4	1429	1442		
4	10	P0C0L4|CO4A_HUMAN	Q.KPRLLLFSPSVVHLGVPLSVGVQLQDVPRGQVVKGSVFLR.N	Y	59.47	4320.531	40	-1.3	721.0949	6	58.07		5	10561	17041103.	1	20	59		
4	10	P0C0L4|CO4A_HUMAN	K.DDPDAPLQPVTPLQL.F	Y	58.92	1617.825	15	1	809.9206	2	66.78	3.71E+05	5	12190	17041103.	1	1429	1443		
4	10	P0C0L4|CO4A_HUMAN	R.GLEEELQFSLGSK.I	Y	54.21	1435.72	13	0.3	718.8672	2	52.85	5.70E+05	6	9695	17041104.	1	1353	1365		
4	10	P0C0L4|CO4A_HUMAN	R.TLEIPGNSDPNMIPDGDFNSYVRVT.A	Y	53.85	2750.286	25	4	1376.156	2	60.16	2.10E+05	5	10954	17041103.	3	957	981		
4	10	P0C0L4|CO4A_HUMAN	L.LFSPSVVHLGVPLSVGVQ.L	Y	53.73	1834.035	18	-2.2	918.0229	2	61.5	3.34E+05	5	11203	17041103.	1	25	42		

4	10	P0C0L4|CO4A_HUMAN	R.TLEIPGNSDPNM(+15.99)IPDGDFNSYVRVT.A	Y	53.44	2766.281	25	5.2	923.1056	3	56.97	1.80E+05	5	10357	17041103.	1	957	981	Oxidation	M12:Oxidation (M):1000.00
4	10	P0C0L4|CO4A_HUMAN	Q.LSVSAGSPHPAIARLT.V	Y	49.65	1575.873	16	2.5	526.2997	3	37.03	8.15E+05	5	6625	17041103.	1	446	461		
4	10	P0C0L4|CO4A_HUMAN	T.VAAPPSGGPGFLSIERPDSRPPRVG.D	Y	45.41	2515.329	25	0.5	629.8399	4	42.8	1.39E+07	5	7713	17041103.	2	462	486		
4	10	P0C0L4|CO4A_HUMAN	L.LFSPSVVHLGVPLS.V	Y	44.84	1450.818	14	1.4	726.4175	2	57.53	2.81E+05	5	10461	17041103.	1	25	38		
4	10	P0C0L4|CO4A_HUMAN	D.PDAPLQPVTPLQLFEGRRN.R	Y	41.52	2147.149	19	1.2	716.7244	3	55.84	6.69E+05	5	10140	17041103.	3	1431	1449		
4	10	P0C0L4|CO4A_HUMAN	D.DPDAPLQPVTPLQLFEGRRN.R	Y	40.91	2262.176	20	0.7	755.0663	3	59.31	7.32E+04	5	10793	17041103.	1	1430	1449		
4	10	P0C0L4|CO4A_HUMAN	L.FSPSVVHLGVPLS.V	Y	39.23	1337.734	13	0.5	669.8748	2	52.87	1.66E+05	5	9584	17041103.	1	26	38		
4	10	P0C0L4|CO4A_HUMAN	Y.DELPAKDDPDAPLQPVTPLQLFEGRRN.R	Y	32.62	3030.541	27	1.8	758.6439	4	55.82	1.42E+05	6	10240	17041104.	1	1423	1449		
4	10	P0C0L4|CO4A_HUMAN	L.LFSPSVVHLGVPLSVGVQLQDVPRGQVVK.G	Y	28.73	3053.739	29	-0.1	764.442	4	56.75	6.62E+04	5	10315	17041103.	1	25	53		
4	10	P0C0L4|CO4A_HUMAN	L.LFSPSVVHLG.V	Y	28.31	1054.581	10	0.7	528.2982	2	47.6	5.29E+04	5	8603	17041103.	1	25	34		
4	10	P0C0L4|CO4A_HUMAN	A.APPSGGPGFLSIERPDSRPPRVG.D	Y	27.21	2345.224	23	0.7	587.3137	4	40.79	2.44E+05	5	7333	17041103.	1	464	486		
6	123	P02675|FIBB_HUMAN	R.EEAPSLRPAPPPISGGGY.R	Y	86.32	1793.895	18	0.1	897.9548	2	44.37	1.98E+06	6	7962	17041104.	4	54	71		
6	123	P02675|FIBB_HUMAN	E.EAPSLRPAPPPISGGGY.R	Y	67.22	1664.852	17	0.2	833.4335	2	43.31	3.88E+05	6	7771	17041104.	1	55	71		
6	123	P02675|FIBB_HUMAN	K.REEAPSLRPAPPPISGGGY.R	Y	66.32	1949.996	19	0.9	651.0065	3	39.27	5.77E+05	6	6994	17041104.	1	53	71		
6	123	P02675|FIBB_HUMAN	S.Q(-17.03)GVNDNEEGFFSARGHRPLDKKREEAPSLRPAPPPISGGGY.R	Y	62.64	4415.179	41	1.1	884.0441	5	42.29	2.06E+07	6	7582	17041104.	7	31	71	Pyro-glu fr	Q1:Pyro-glu from Q:1000.00
6	123	P02675|FIBB_HUMAN	S.Q(-17.03)GVNDNEEGFFSAR.G	Y	62.4	1551.659	14	0.5	776.8371	2	49.87	3.82E+06	6	9134	17041104.	3	31	44	Pyro-glu fr	Q1:Pyro-glu from Q:1000.00
6	123	P02675|FIBB_HUMAN	S.Q(-17.03)GVNDNEEGFFSARGH.R	Y	62	1745.739	16	0.2	873.8771	2	43.17	9.14E+04	6	7746	17041104.	1	31	46	Pyro-glu fr	Q1:Pyro-glu from Q:1000.00
6	123	P02675|FIBB_HUMAN	S.Q(-17.03)GVNDNEEGFFSA.R	Y	58.58	1395.558	13	-0.2	698.7861	2	63.55	6.76E+07	5	11588	17041103.	13	31	43	Pyro-glu fr	Q1:Pyro-glu from Q:1000.00
6	123	P02675|FIBB_HUMAN	S.Q(-17.03)GVNDNEEGFFSARGHRPLDKKRE.E	Y	58.45	2768.338	24	0.5	554.6751	5	31.89	2.23E+05	5	5669	17041103.	2	31	54	Pyro-glu fr	Q1:Pyro-glu from Q:1000.00
6	123	P02675|FIBB_HUMAN	K.KREEAPSLRPAPPPISGGGY.R	Y	57.68	2078.091	20	1.8	693.7054	3	35.47	1.41E+05	6	6286	17041104.	1	52	71		
6	123	P02675|FIBB_HUMAN	S.Q(-17.03)GVNDNEEGFFSARGHRPLDKKREEAPSLRPAPPPISGGGYR.A	Y	57.63	4571.28	42	1.5	762.8885	6	40.21	2.88E+07	5	7224	17041103.	6	31	72	Pyro-glu fr	Q1:Pyro-glu from Q:1000.00
6	123	P02675|FIBB_HUMAN	S.Q(-17.03)GVNDNEEGFFSARGHRPLD.K	Y	56.24	2227.004	20	-0.3	743.3417	3	43.03	1.58E+07	6	7720	17041104.	4	31	50	Pyro-glu fr	Q1:Pyro-glu from Q:1000.00
6	123	P02675|FIBB_HUMAN	L.DKKREEAPSLRPAPPPISGGGY.R	Y	53.31	2321.213	22	0.6	581.3108	4	34.68	4.21E+05	6	6143	17041104.	1	50	71		
6	123	P02675|FIBB_HUMAN	S.Q(-17.03)GVNDNEEGFFSARGHRPLDKKR.E	Y	49.46	2639.295	23	1	528.8668	5	31.53	3.43E+05	5	5601	17041103.	1	31	53	Pyro-glu fr	Q1:Pyro-glu from Q:1000.00
6	123	P02675|FIBB_HUMAN	V.NDNEEGFFSA.R	Y	49.32	1128.436	10	0.1	565.2253	2	47.48	5.75E+06	5	8581	17041103.	7	34	43		
6	123	P02675|FIBB_HUMAN	S.Q(-17.03)GVNDNEEGFFSARGHRPLDKKREEAPSLRPAPPPISGGGYRARPAK.A	Y	48.41	5094.604	47	2.2	728.8094	7	36.15		5	6463	17041103.	1	31	77	Pyro-glu fr	Q1:Pyro-glu from Q:1000.00
6	123	P02675|FIBB_HUMAN	S.Q(-17.03)GVNDNEEGFFS.A	Y	48.18	1324.521	12	-1.3	663.2668	2	61.01	6.13E+05	5	11111	17041103.	2	31	42	Pyro-glu fr	Q1:Pyro-glu from Q:1000.00
6	123	P02675|FIBB_HUMAN	G.VNDNEEGFFSARGHRPLD.K	Y	47.66	2058.951	18	0.7	515.7453	4	36.14	3.10E+05	5	6462	17041103.	5	33	50		
6	123	P02675|FIBB_HUMAN	G.VNDNEEGFFSARGHRPLDKKREEAPSLRPAPPPISGGGYR.A	Y	47.43	4403.227	40	0	734.8785	6	36.57	1.19E+06	5	6541	17041103.	1	33	72		
6	123	P02675|FIBB_HUMAN	G.VNDNEEGFFSARGHRPLDK.K	Y	47.29	2187.046	19	0.6	547.769	4	31.79	1.08E+05	6	5616	17041104.	1	33	51		
6	123	P02675|FIBB_HUMAN	S.Q(-17.03)GVNDNEEGFFSARGHRPLDKKREEAPSL.R	Y	47.05	3265.586	29	4.8	817.4078	4	38.05	2.31E+05	5	6816	17041103.	1	31	59	Pyro-glu fr	Q1:Pyro-glu from Q:1000.00
6	123	P02675|FIBB_HUMAN	S.Q(-17.03)GVNDNEEGFFSARGHRPLDKKREEAPS.L	Y	46.97	3152.502	28	0.4	631.508	5	33.51	8.76E+05	5	5973	17041103.	3	31	58	Pyro-glu fr	Q1:Pyro-glu from Q:1000.00
6	123	P02675|FIBB_HUMAN	S.Q(-17.03)GVNDNEEGFFSARGHRPLDKK.R	Y	46.83	2483.194	22	1.3	621.8066	4	33.8	2.18E+05	6	5984	17041104.	1	31	52	Pyro-glu fr	Q1:Pyro-glu from Q:1000.00
6	123	P02675|FIBB_HUMAN	G.VNDNEEGFFSA.R	Y	45.06	1227.504	11	0	614.7595	2	48.11	5.05E+06	5	8698	17041103.	4	33	43		
6	123	P02675|FIBB_HUMAN	K.KREEAPSLRPAPPPISGGGYR.A	Y	44.72	2234.192	21	1.7	559.5562	4	31.3	1.06E+05	6	5523	17041104.	1	52	72		
6	123	P02675|FIBB_HUMAN	R.GHRPLDKKREEAPSLRPAPPPISGGGY.R	Y	44.56	2881.531	27	2.4	577.3149	5	31.45	1.10E+06	6	5551	17041104.	1	45	71		
6	123	P02675|FIBB_HUMAN	V.NDNEEGFFSARGHRPLD.K	Y	44.41	1959.882	17	-0.6	490.9775	4	35.22	1.81E+05	6	6241	17041104.	1	34	50		
6	123	P02675|FIBB_HUMAN	S.Q(-17.03)GVNDNEEGFFSARGHRPLDKKREEAPSLRPAPPPISGGGYRARP.A	Y	43.52	4895.472	45	0.7	700.3608	7	37.95	1.56E+06	6	6745	17041104.	3	31	75	Pyro-glu fr	Q1:Pyro-glu from Q:1000.00
6	123	P02675|FIBB_HUMAN	S.Q(-17.03)GVNDNEEGFFSARGHRPLDKKREEAPSLRPAPPPISGGGYRARPAKAA.A	Y	43.33	5236.678	49	-0.1	749.104	7	36.4	1.10E+05	5	6509	17041103.	1	31	79	Pyro-glu fr	Q1:Pyro-glu from Q:1000.00
6	123	P02675|FIBB_HUMAN	K.IRPFFPQQ	Y	41.81	1031.555	8	1.2	516.7855	2	41.49	7.56E+05	5	7462	17041103.	2	484	491		
6	123	P02675|FIBB_HUMAN	N.DNEEGFFSA.R	Y	39.5	1014.393	9	0.3	508.2039	2	50.32	7.88E+05	6	9222	17041104.	2	35	43		
6	123	P02675|FIBB_HUMAN	S.Q(-17.03)GVNDNEEGFFSARGHRPLDKKREEAPSLRPAPPPIS.G	Y	38.75	4081.052	37	-0.3	817.2173	5	40.18	1.19E+06	5	7219	17041103.	1	31	67	Pyro-glu fr	Q1:Pyro-glu from Q:1000.00
6	123	P02675|FIBB_HUMAN	S.Q(-17.03)GVNDNEEGFFSARGHRPLDKKREE.A	Y	38.13	2897.38	25	0.4	580.4836	5	32.34	2.23E+05	5	5756	17041103.	1	31	55	Pyro-glu fr	Q1:Pyro-glu from Q:1000.00
6	123	P02675|FIBB_HUMAN	S.Q(-17.03)GVNDNEEGFFSARGHRPLDKKREEAPSLRPAPPPISGGGYRARPA.K	Y	31.53	4966.509	46	-2.9	621.8191	8	38.19		5	6843	17041103.	1	31	76	Pyro-glu fr	Q1:Pyro-glu from Q:1000.00
5	13997	P0DJI8|SAA1_HUMAN	R.FFGHGAEDSLADQAANEWGRSGKDPNHFRPAGLPEKY	N	70.85	4070.905	37	-1.5	815.187	5	46.47	6.07E+06	6	8362	17041104.	6	86	122		
5	13997	P0DJI8|SAA1_HUMAN	A.ISDARENIQRFFGHGAEDSLADQAANEWGRSGKDPNHFRPAGLPEKY	Y	63.45	5253.515	47	-0.4	751.5092	7	49.91	9.58E+06	6	9141	17041104.	9	76	122		
5	13997	P0DJI8|SAA1_HUMAN	D.SLADQAANEWGRSGKDPNHFRPAGLPEKY	N	63.28	3210.559	29	2	643.1204	5	41.89	2.14E+07	6	7508	17041104.	4	94	122		
5	13997	P0DJI8|SAA1_HUMAN	F.FGHGAEDSLADQAANEWGRSGKDPNHFRPAGLPEKY	N	62.12	3923.836	36	-0.2	785.7744	5	43.5	1.56E+06	6	7804	17041104.	7	87	122		
5	13997	P0DJI8|SAA1_HUMAN	S.RSFFSFLGEAFD.G	N	61.51	1421.662	12	0	711.838	2	68.13	5.73E+06	6	12836	17041104.	1	19	30		
5	13997	P0DJI8|SAA1_HUMAN	D.PNHFRPAGLPEKY	N	61.5	1524.784	13	1.4	509.2692	3	30.11	2.14E+06	6	5307	17041104.	4	110	122		
5	13997	P0DJI8|SAA1_HUMAN	A.AEAISDARENIQRFFGHGAEDSLADQAANEWGRSGKDPNHFRPAGLPEKY	Y	61.07	5524.632	50	0.6	790.2409	7	52.27	2.44E+06	6	9588	17041104.	3	73	122		
5	13997	P0DJI8|SAA1_HUMAN	A.ANEWGRSGKDPNHFRPAGLPEKY	N	60.1	2625.284	23	-1.1	657.3275	4	69.38	1.90E+06	6	13138	17041104.	3	100	122		
5	13997	P0DJI8|SAA1_HUMAN	F.DGARDMWRAYSDMREANYIGSDKYFHARGNYDAAKRGP.G	N	59.47	4410.02	38	-2.5	736.0087	6	44.62	1.22E+06	6	8009	17041104.	4	30	67		
5	13997	P0DJI8|SAA1_HUMAN	D.Q(-17.03)AANEWGRSGKDPNHFRPAGLPEKY	N	56.62	2807.353	25	0.1	562.4779	5	37.64	7.85E+05	6	6688	17041104.	2	98	122	Pyro-glu fr	Q1:Pyro-glu from Q:1000.00
5	13997	P0DJI8|SAA1_HUMAN	D.ARENIQRFFGHGAEDSLADQAANEWGRSGKDPNHFRPAGLPEKY	N	56.14	4938.372	44	-0.1	706.489	7	46.07	2.07E+06	6	8283	17041104.	3	79	122		
5	13997	P0DJI8|SAA1_HUMAN	R.SFFSFLGEAFD.G	N	55.75	1265.56	11	0	633.7875	2	79.66	1.21E+06	6	15513	17041104.	1	20	30		
5	13997	P0DJI8|SAA1_HUMAN	A.DQAANEWGRSGKDPNHFRPAGLPEKY	N	54.27	2939.406	26	0	735.8588	4	35.95	9.18E+05	6	6374	17041104.	4	97	122		
5	13997	P0DJI8|SAA1_HUMAN	S.FLGEAFDGARDMWRAYSDMREANYIGSDKYFHARGNYDAAKRGP.G	N	54.22	5074.341	44	-0.6	635.2996	8	54.1	2.86E+06	6	9927	17041104.	3	24	67		
5	13997	P0DJI8|SAA1_HUMAN	D.QAANEWGRSGKDPNHFRPAGLPEKY	N	54.11	2824.379	25	1.9	565.8842	5	34.18	4.67E+06	6	6053	17041104.	5	98	122		
5	13997	P0DJI8|SAA1_HUMAN	Q.RFFGHGAEDSLADQAANEWGRSGKDPNHFRPAGLPEKY	N	53.08	4227.006	38	-0.3	705.5081	6	44.52	2.91E+05	6	7989	17041104.	2	85	122		
5	13997	P0DJI8|SAA1_HUMAN	R.FFGHGAEDSLADQAANEWGRSGKDPNHFRPAGLPEK.Y	N	52.54	3907.841	36	0.4	652.3145	6	44.7	3.25E+05	6	8022	17041104.	2	86	121		
5	13997	P0DJI8|SAA1_HUMAN	H.GAEDSLADQAANEWGRSGKDPNHFRPAGLPEKY	N	51.33	3582.688	33	0.2	717.5449	5	44.51	8.14E+05	6	7987	17041104.	2	90	122		
5	13997	P0DJI8|SAA1_HUMAN	K.DPNHFRPAGLPEKY	N	50.78	1639.811	14	0.2	547.611	3	34.26	1.21E+05	6	6067	17041104.	1	109	122		
5	13997	P0DJI8|SAA1_HUMAN	G.HGAEDSLADQAANEWGRSGKDPNHFRPAGLPEKY	N	49.9	3719.746	34	0.5	620.9653	6	42.03	7.85E+05	6	7534	17041104.	2	89	122		
5	13997	P0DJI8|SAA1_HUMAN	R.SGKDPNHFRPAGLPEKY	N	47.78	1911.959	17	-0.5	478.9968	4	29.99	6.34E+05	6	5284	17041104.	2	106	122		
5	13997	P0DJI8|SAA1_HUMAN	A.NEWGRSGKDPNHFRPAGLPEKY	N	47.65	2554.247	22	0.1	639.569	4	33.26	1.11E+05	6	5885	17041104.	3	101	122		
5	13997	P0DJI8|SAA1_HUMAN	S.DARENIQRFFGHGAEDSLADQAANEWGRSGKDPNHFRPAGLPEKY	N	46.39	5053.399	45	1.6	722.9225	7	50.35	2.20E+05	5	9112	17041103.	1	78	122		
5	13997	P0DJI8|SAA1_HUMAN	F.SFLGEAFDGARDMWRAYSDMREANYIGSDKYFHARGNYDAAKRGP.G	N	44.25	5161.374	45	-1.6	646.1779	8	59.18	6.12E+05	6	10859	17041104.	1	23	67		
5	13997	P0DJI8|SAA1_HUMAN	A.DQAANEWGRSGKDPNHFRPA.G	N	44.15	2252.047	20	0.5	564.0193	4	28.75	2.75E+05	6	5059	17041104.	1	97	116		
5	13997	P0DJI8|SAA1_HUMAN	D.ARENIQRFFGHGAED.S	N	41.07	1745.823	15	0.5	437.4633	4	32.45	4.46E+05	6	5736	17041104.	2	79	93		
5	13997	P0DJI8|SAA1_HUMAN	L.ADQAANEWGRSGKDPNHFRPAGLPEKY	N	40.39	3010.443	27	-0.3	603.0958	5	35.23	4.66E+05	6	6244	17041104.	1	96	122		
5	13997	P0DJI8|SAA1_HUMAN	I.SDARENIQRFFGHGAEDSLADQAANEWGRSGKDPNHFRPAGLPEKY	Y	39.34	5140.431	46	1.1	857.7467	6	49	7.96E+05	6	8948	17041104.	1	77	122		
5	13997	P0DJI8|SAA1_HUMAN	L.GEAFDGARDMWRAYSDMREANYIGSDKYFHARGNYDAAKRGP.G	N	30.79	4814.189	42	0.8	602.7814	8	49.33	2.63E+05	5	8921	17041103.	1	26	67		
5	13997	P0DJI8|SAA1_HUMAN	R.GNYDAAKRGPGGVWAAEAISDARENIQRFFGHGAEDSLADQAANEWGRSGKDPNHFRPAGLPEKY	Y	28.02	7024.359	65	1.7	703.4443	10	59	1.14E+06	6	10826	17041104.	1	58	122		
5	13997	P0DJI8|SAA1_HUMAN	S.RSFFSFL.G	N	27.41	902.465	7	-3.1	452.2384	2	58.64	1.31E+05	6	10759	17041104.	1	19	25		
5	13997	P0DJI8|SAA1_HUMAN	P.GGVWAAEAISDARENIQRFFGHGAEDSLADQAANEWGRSGKDPNHFRPAGLPEKY	Y	27.36	5994.86	55	0.5	750.3651	8	60.36	7.58E+05	6	11081	17041104.	1	68	122		
18	96	P02766|TTHY_HUMAN	N.DSGPRRYTIAALLSPYSYSTTAVVTNPKE	Y	87.54	3156.609	29	-1.1	790.1587	4	55.32	7.58E+06	6	10150	17041104.	6	119	147		
18	96	P02766|TTHY_HUMAN	S.GPRRYTIAALLSPYSYSTTAVVTNPKE	Y	85.9	2954.55	27	0.9	739.6454	4	52.19	1.39E+06	6	9574	17041104.	2	121	147		
18	96	P02766|TTHY_HUMAN	S.GPRRYTIAALLSPYSYSTTAVVTNPK.E	Y	83.53	2825.508	26	-2.5	707.3824	4	51.81	1.08E+06	6	9503	17041104.	3	121	146		
18	96	P02766|TTHY_HUMAN	D.SGPRRYTIAALLSPYSYSTTAVVTNPKE	Y	78.42	3041.582	28	-0.3	761.4026	4	52.3	5.81E+05	5	9478	17041103.	2	120	147		
18	96	P02766|TTHY_HUMAN	G.PRRYTIAALLSPYSYSTTAVVTNPKE	Y	76.64	2897.529	26	1	725.3901	4	52.07	1.37E+06	6	9553	17041104.	2	122	147		
18	96	P02766|TTHY_HUMAN	L.SPYSYSTTAVVTNPKE	Y	72.22	1742.836	16	0.3	872.4257	2	37.27	1.77E+06	5	6669	17041103.	3	132	147		
18	96	P02766|TTHY_HUMAN	A.ALLSPYSYSTTAVVTNPKE	Y	60.45	2040.042	19	-1.4	1021.027	2	48.54	5.70E+04	5	8777	17041103.	1	129	147		
18	96	P02766|TTHY_HUMAN	T.IAALLSPYSYSTTAVVTNPKE	Y	58.57	2224.163	21	-0.3	1113.088	2	53.32	4.41E+05	5	9668	17041103.	1	127	147		
18	96	P02766|TTHY_HUMAN	F.TANDSGPRRYTIAALLSPYSYSTTAVVTNPKE	Y	35.91	3442.737	32	-0.6	861.691	4	53.93	6.81E+04	5	9784	17041103.	1	116	147		
18	96	P02766|TTHY_HUMAN	G.ISPFHEHAEVVFTANDSGPRRYTIAALLSPYSYSTTAVVTNPKE	Y	34.86	4835.419	44	1.8	806.912	6	55.66		5	10107	17041103.	1	104	147		

15	60	P08697|A2AP_HUMAN	A.MEPLGRQLTSGPNQEQVSPLTLLKLGNQEPGGQTALK.S	Y	93.04	3929.068	37	0.2	983.2744	4	55.21	4.56E+06	5	10025	17041103.	2	28	64	
15	60	P08697|A2AP_HUMAN	A.MEPLGRQLTSGPNQEQVSPLTLLK.L	Y	76.41	2635.4	24	0.7	879.4747	3	54.01	2.91E+06	5	9799	17041103.	2	28	51	
15	60	P08697|A2AP_HUMAN	A.M(+15.99)EPLGRQLTSGPNQEQVSPLTLLKLGNQEPGGQTALK.S	Y	73.18	3945.063	37	1.9	987.2749	4	53.78	8.32E+05	5	9789	17041103.	1	28	64	Oxidation M1:Oxidation (M):1000.00
15	60	P08697|A2AP_HUMAN	P.NQEQVSPLTLLKLGNQEPGGQTALK.S	Y	70.34	2662.429	25	-0.8	888.4829	3	53.52	7.20E+06	5	9708	17041103.	3	40	64	
15	60	P08697|A2AP_HUMAN	K.LVPPMEEDYPQFGSPK	Y	69.24	1832.866	16	-0.8	917.4393	2	53.03	1.12E+06	5	9614	17041103.	2	476	491	
15	60	P08697|A2AP_HUMAN	A.M(+15.99)EPLGRQLTSGPNQEQVSPLTLLK.L	Y	68.53	2651.395	24	-0.3	884.8054	3	51.08	6.48E+05	5	9250	17041103.	1	28	51	Oxidation M1:Oxidation (M):1000.00
15	60	P08697|A2AP_HUMAN	T.SGPNQEQVSPLTLLK.L	Y	62.08	1609.868	15	-0.7	805.9405	2	49.51	3.14E+04	5	8954	17041103.	1	37	51	
15	60	P08697|A2AP_HUMAN	K.LVPPM(+15.99)EEDYPQFGSPK	Y	61.41	1848.86	16	2.2	925.4395	2	46.96	2.84E+05	5	8482	17041103.	1	476	491	Oxidation M5:Oxidation (M):1000.00
15	60	P08697|A2AP_HUMAN	A.MEPLGRQLTSGP.N	Y	59.31	1284.65	12	-0.2	643.332	2	38.5	4.37E+06	6	6848	17041104.	2	28	39	
15	60	P08697|A2AP_HUMAN	M.EPLGRQLTSGP.N	Y	55.75	1153.609	11	0.4	577.8121	2	32.57	1.51E+06	6	5758	17041104.	1	29	39	
15	60	P08697|A2AP_HUMAN	A.M(+15.99)EPLGRQLTSGP.N	Y	53.44	1300.645	12	0	651.3295	2	34.08	7.16E+05	6	6034	17041104.	3	28	39	Oxidation M1:Oxidation (M):1000.00
15	60	P08697|A2AP_HUMAN	P.NQEQVSPLTLLK.L	Y	52.68	1368.761	12	0.6	685.3883	2	49.1	4.07E+06	5	8880	17041103.	2	40	51	
15	60	P08697|A2AP_HUMAN	P.NQEQVSPLTLLKLG.N	Y	49.21	1538.867	14	2.5	770.4426	2	59.63	3.20E+05	5	10854	17041103.	1	40	53	
15	60	P08697|A2AP_HUMAN	R.MSLSSFSVNRPFLFFIFEDTTGLPLFVGSVRNPNPSAPRELKEQQ.D	Y	48.72	5098.602	45	-2.7	1020.725	5	72.43	8.85E+04	5	13244	17041103.	1	404	448	
15	60	P08697|A2AP_HUMAN	A.MEPLGRQLTSGPNQEQVSPLTL.L	Y	35.28	2394.221	22	-2.3	799.0792	3	54.48	6.08E+04	5	9888	17041103.	1	28	49	
19	2	P02647|APOA1_HUMAN	V.LESFKVSFLSALEEYTKKLNTQ	Y	86.92	2574.358	22	0.9	644.5974	4	62.99	3.94E+05	6	11577	17041104.	1	246	267	
19	2	P02647|APOA1_HUMAN	L.ESFKVSFLSALEEYTKKLNTQ	Y	68.73	2461.274	21	-2.8	821.4296	3	61.93	1.83E+05	5	11283	17041103.	2	247	267	
19	2	P02647|APOA1_HUMAN	D.LATVYVDVLKDSGRDYVSQFEGSALGKQL.N	Y	63.81	3157.629	29	-0.3	790.4144	4	60.18	7.36E+05	6	11045	17041104.	1	38	66	
19	2	P02647|APOA1_HUMAN	Q.DEPPQSPWDRVKDLATVYVDVLK.D	Y	61.88	2669.37	23	0.2	890.7975	3	68.31	2.40E+06	5	12473	17041103.	4	25	47	
19	2	P02647|APOA1_HUMAN	E.SFKVSFLSALEEYTKKLNTQ	Y	59.94	2332.231	20	0.4	584.0654	4	60.45	2.82E+05	5	11008	17041103.	2	248	267	
19	2	P02647|APOA1_HUMAN	K.VSFLSALEEYTKKLNTQ	Y	57.41	1970.036	17	0.6	657.6863	3	62.23	7.55E+05	5	11339	17041103.	1	251	267	
19	2	P02647|APOA1_HUMAN	D.NLEKETEGLRQEM.S	Y	52.36	1575.756	13	0.6	526.2596	3	36.47	1.05E+05	6	6469	17041104.	1	98	110	
19	2	P02647|APOA1_HUMAN	L.SALEEYTKKLNTQ	Y	49.85	1523.783	13	0.6	508.9353	3	35.59	2.35E+05	6	6309	17041104.	1	255	267	
19	2	P02647|APOA1_HUMAN	V.SFLSALEEYTKKLNTQ	Y	46.15	1870.968	16	4.4	624.6659	3	60.6	3.40E+05	6	11127	17041104.	1	252	267	
19	2	P02647|APOA1_HUMAN	Q.DEPPQSPWDRVKD.L	Y	43.02	1567.727	13	0.2	523.5829	3	35.28	7.83E+05	6	6252	17041104.	1	25	37	
19	2	P02647|APOA1_HUMAN	R.AELQEGARQKLHELQ.E	Y	36.61	1748.917	15	0.6	583.9799	3	29.8	2.32E+05	5	5277	17041103.	1	148	162	
19	2	P02647|APOA1_HUMAN	L.NLKLLDNWD.S	Y	34.43	1129.577	9	1.1	565.7963	2	50.64	3.02E+05	6	9282	17041104.	1	67	75	
19	2	P02647|APOA1_HUMAN	R.AELQEGARQKLHELQEKLSPLG.E	Y	30.13	2473.329	22	9	619.345	4	47.01		5	8491	17041103.	1	148	169	
19	2	P02647|APOA1_HUMAN	Q.DEPPQSPWDRVKDLATVY.V	Y	28.13	2115.027	18	0.3	706.0166	3	58.68	4.75E+06	6	10767	17041104.	1	25	42	
12	86	P01011|AACT_HUMAN	A.LVETRTIVRFNRPFLMIIVPTDTQNIFFMSKVTNPKQA	Y	88.4	4464.418	38	-4.9	893.8864	5	60.84	9.81E+06	6	11173	17041104.	10	386	423	
12	86	P01011|AACT_HUMAN	L.SALVETRTIVRFNRPFLMIIVPTDTQNIFFMSKVTNPKQA	Y	81.03	4622.487	40	-1.3	771.4207	6	62.15	1.91E+06	5	11325	17041103.	6	384	423	
12	86	P01011|AACT_HUMAN	L.VETRTIVRFNRPFLMIIVPTDTQNIFFMSKVTNPKQA	Y	77.45	4351.334	37	5.9	871.2791	5	60.21	8.04E+05	6	11051	17041104.	3	387	423	
12	86	P01011|AACT_HUMAN	E.TRTIVRFNRPFLMIIVPTDTQNIFFMSKVTNPKQA	Y	70.81	4123.223	35	-1.3	825.6508	5	59.68	6.51E+05	5	10863	17041103.	1	389	423	
12	86	P01011|AACT_HUMAN	D.TQNIFFMSKVTNPKQA	Y	62.78	1852.951	16	0.3	618.6577	3	42.27	2.94E+05	6	7580	17041104.	1	408	423	
12	86	P01011|AACT_HUMAN	S.ALVETRTIVRFNRPFLMIIVPTDTQNIFFMSKVTNPKQA	Y	60.96	4535.455	39	0.6	756.9168	6	62.17	4.61E+05	5	11328	17041103.	3	385	423	
12	86	P01011|AACT_HUMAN	A.LVETRTIVRFNRPFLM(+15.99)IIVPTDTQNIFFMSKVTNPKQA	Y	60.04	4480.413	38	-0.2	747.7426	6	59.38	2.41E+05	5	10806	17041103.	1	386	423	Oxidation M16:Oxidation (M):93.03
12	86	P01011|AACT_HUMAN	P.NSPLDEENLT.Q	Y	50.92	1130.509	10	1.3	566.2626	2	41.08	4.10E+05	5	7387	17041103.	2	26	35	
12	86	P01011|AACT_HUMAN	A.LVETRTIVRFNRPFLMIIVPTDTQNIFFM(+15.99)SKVTNPKQA	Y	46.83	4480.413	38	1.9	747.7441	6	58.82		6	10793	17041104.	3	386	423	Oxidation M29:Oxidation (M):82.05
12	86	P01011|AACT_HUMAN	M.IIVPTDTQNIFFMSKVTNPKQA	Y	39.41	2491.315	22	0.7	831.446	3	54.96	1.07E+06	5	9978	17041103.	2	402	423	
12	86	P01011|AACT_HUMAN	C.HPNSPLDEENLTQENQDRGTHVDLGLA.S	Y	32.89	2998.402	27	-0.4	750.6074	4	43.75	9.89E+04	5	7886	17041103.	1	24	50	
14	1182	P01275|GLUC_HUMAN	Q.RSLQDTEEKSRSFSASQADPLSDPDQMNED.K	Y	83.04	3382.485	30	-0.2	846.6284	4	39.36	2.31E+07	6	7009	17041104.	3	21	50	
14	1182	P01275|GLUC_HUMAN	Q.RSLQDTEEKSRSFSASQADPLSDPDQMNE.D	Y	70.61	3267.459	29	-2.4	817.8699	4	39.1	3.67E+06	6	6960	17041104.	3	21	49	
14	1182	P01275|GLUC_HUMAN	S.LQDTEEKSRSFSASQADPLSDPDQMNED.K	Y	69.8	3139.352	28	-0.7	1047.457	3	40.69	7.40E+06	6	7272	17041104.	3	23	50	
14	1182	P01275|GLUC_HUMAN	Q.RSLQDTEEKSRSFSASQADPLSDPDQM(+15.99)NE.D	Y	68.46	3283.453	29	0.3	821.8708	4	36.98	6.27E+05	6	6564	17041104.	1	21	49	Oxidation M27:Oxidation (M):1000.00
14	1182	P01275|GLUC_HUMAN	R.SLQDTEEKSRSFSASQADPLSDPDQMNED.K	Y	68.27	3226.384	29	0	1076.469	3	43.12	1.28E+06	6	7736	17041104.	1	22	50	
14	1182	P01275|GLUC_HUMAN	S.LQDTEEKSRSFSASQADPLSDPDQM(+15.99)NED.K	Y	66.04	3155.347	28	0.2	1052.79	3	37.99	1.40E+06	6	6753	17041104.	1	23	50	Oxidation M25:Oxidation (M):1000.00
14	1182	P01275|GLUC_HUMAN	R.HSQGTFTSDYSKYLDSRRAQD.F	Y	64.38	2461.126	21	-0.7	616.2883	4	35.48	4.00E+05	6	6288	17041104.	2	53	73	
14	1182	P01275|GLUC_HUMAN	S.LQDTEEKSRSFSASQADPLSDPDQMNE.D	Y	59.21	3024.325	27	0.3	1009.116	3	40.51	3.37E+06	6	7237	17041104.	1	23	49	
14	1182	P01275|GLUC_HUMAN	R.HSQGTFTSDYSKYLDSRRAQDFVQWLMNT.K	Y	55.81	3480.616	29	-0.8	871.1605	4	62.7	2.62E+06	6	11520	17041104.	1	53	81	
14	1182	P01275|GLUC_HUMAN	S.LQDTEEKSRSFSASQADPLSDPDQM(+15.99)NE.D	Y	55.12	3040.32	27	1.7	1014.449	3	37.68	5.74E+05	6	6695	17041104.	1	23	49	Oxidation M25:Oxidation (M):1000.00
14	1182	P01275|GLUC_HUMAN	L.Q(-17.03)DTEEKSRSFSASQADPLSDPDQMNED.K	Y	52.36	3009.242	27	2.8	1004.091	3	44.66	1.47E+05	6	8015	17041104.	1	24	50	Pyro-glu fr Q1:Pyro-glu from Q:1000.00
14	1182	P01275|GLUC_HUMAN	R.SLQDTEEKSRSFSASQADPLSDPDQMNE.D	Y	47.49	3111.357	28	0.4	1038.127	3	43.02	3.33E+05	6	7718	17041104.	1	22	49	
14	1182	P01275|GLUC_HUMAN	Q.RSLQDTEEKSRSFSASQADPLSDPDQM(+15.99)NED.K	Y	46.99	3398.481	30	0.2	1133.834	3	37.17	1.03E+05	6	6599	17041104.	1	21	50	Oxidation M27:Oxidation (M):1000.00
14	1182	P01275|GLUC_HUMAN	R.DFPEEVAIVEELGRRHADGSFSDEMNTILDNLAARDFINWLIQTKITD.R	Y	43.7	5503.699	48	1.8	918.2922	6	81.32	9.88E+06	6	16054	17041104.	1	131	178	
14	1182	P01275|GLUC_HUMAN	R.HDEFERHAEGTFTSDVSSYLEGQAAKEFIAWLVKGRG(-.98).R	Y	38.99	4166.024	37	-1.1	695.3439	6	66.26	6.52E+05	6	12336	17041104.	1	92	128	Amidation G37:Amidation:1000.00
14	1182	P01275|GLUC_HUMAN	R.SLQDTEEKSRSFSASQADPLSDPDQM(+15.99)NED.K	Y	34.33	3242.379	29	-3	1081.797	3	40.77	1.98E+05	6	7286	17041104.	1	22	50	Oxidation M26:Oxidation (M):1000.00
9	238	P81172|HEPC_HUMAN	G.SVFPQQTGQLAELQPQDRAGARASWMPMFQ.R	Y	73.93	3374.629	30	1.7	1125.886	3	59.33	1.48E+07	6	10886	17041104.	7	25	54	
9	238	P81172|HEPC_HUMAN	G.SVFPQQTGQLAELQPQDRAGARASWMPM.F	Y	67.74	3099.502	28	0.6	1034.175	3	54.75	2.50E+07	6	10046	17041104.	6	25	52	
9	238	P81172|HEPC_HUMAN	G.SVFPQQTGQLAELQPQDRAGARASWMP.M	Y	67.17	2968.461	27	1.1	990.4955	3	53.32	1.01E+07	6	9783	17041104.	4	25	51	
9	238	P81172|HEPC_HUMAN	G.SVFPQQTGQLAELQPQDRAGARA.S	Y	63.04	2467.257	23	0.8	823.4269	3	42.53	3.16E+06	6	7628	17041104.	4	25	47	
9	238	P81172|HEPC_HUMAN	G.SVFPQQTGQLAELQPQDRAGARAS.W	Y	62.18	2554.289	24	0.4	852.4372	3	42.79	2.92E+07	6	7676	17041104.	5	25	48	
9	238	P81172|HEPC_HUMAN	G.SVFPQQTGQLAELQPQDRAGA.R	Y	55.79	2240.118	21	2.6	747.7153	3	47.48	3.99E+05	5	8579	17041103.	1	25	45	
9	238	P81172|HEPC_HUMAN	P.QQTGQLAELQPQDRAGARAS.W	Y	55.5	2124.067	20	-0.1	709.0296	3	30.86	1.07E+05	6	5443	17041104.	1	29	48	
9	238	P81172|HEPC_HUMAN	G.SVFPQQTGQLAELQPQDRAGARASWM(+15.99)P.M	Y	53.92	2984.456	27	4.1	995.8301	3	49.28	1.04E+06	6	9011	17041104.	2	25	51	Oxidation M26:Oxidation (M):1000.00
9	238	P81172|HEPC_HUMAN	S.VFPQQTGQLAELQPQDRAGARAS.W	Y	52.66	2467.257	23	2.8	823.4285	3	40.04	1.19E+06	6	7150	17041104.	3	26	48	
9	238	P81172|HEPC_HUMAN	P.Q(-17.03)QTGQLAELQPQDRAGA.R	Y	52.16	1792.87	17	0.4	897.4428	2	43.84	5.31E+04	6	7866	17041104.	1	29	45	Pyro-glu fr Q1:Pyro-glu from Q:1000.00
9	238	P81172|HEPC_HUMAN	G.SVFPQQTGQLAE.L	Y	47.19	1303.641	12	-0.6	652.8273	2	44.13	8.90E+05	6	7920	17041104.	2	25	36	
9	238	P81172|HEPC_HUMAN	G.SVFPQQTGQLAELQPQDRAGARASWMPM(+15.99).F	Y	46.79	3115.497	28	0.4	1039.507	3	52.22	1.73E+06	5	9463	17041103.	2	25	52	Oxidation M28:Oxidation (M):26.31
9	238	P81172|HEPC_HUMAN	G.SVFPQQTGQLAELQPQDRAGARASWMPM(+15.99)FQ.R	Y	42.78	3390.624	30	2.1	848.665	4	56.14	4.03E+05	6	10299	17041104.	2	25	54	Oxidation M28:Oxidation (M):14.04
9	238	P81172|HEPC_HUMAN	G.SVFPQQTGQLAELQPQDRAGAR.A	Y	38.08	2396.22	22	2.1	799.7488	3	42.79	5.23E+04	5	7710	17041103.	1	25	46	
9	238	P81172|HEPC_HUMAN	G.SVFPQQTGQLAELQP.Q	Y	36.87	1641.836	15	-5.6	821.9208	2	55.6	6.06E+04	6	10199	17041104.	1	25	39	
9	238	P81172|HEPC_HUMAN	G.SVFPQQTGQLAELQPQDRAGARASWM(+15.99)PM.F	Y	35.65	3115.497	28	0.5	779.8819	4	51.32	5.63E+04	5	9295	17041103.	1	25	52	Oxidation M26:Oxidation (M):14.04
9	238	P81172|HEPC_HUMAN	G.SVFPQQTGQLAELQPQDRAGARASWM(+15.99)PMFQ.R	Y	31.98	3390.624	30	4.9	1131.221	3	54.8	1.04E+06	6	10054	17041104.	1	25	54	Oxidation M26:Oxidation (M):14.04
8	13993	P0DJI9|SAA2_HUMAN	S.RSFFSFLGEAFD.G	N	61.51	1421.662	12	0	711.838	2	68.13	5.73E+06	6	12836	17041104.	1	19	30	
8	13993	P0DJI9|SAA2_HUMAN	D.PNHFRPAGLPEKY	N	61.5	1524.784	13	1.4	509.2692	3	30.11	2.14E+06	6	5307	17041104.	4	110	122	
8	13993	P0DJI9|SAA2_HUMAN	F.DGARDMWRAYSDMREANYIGSDKYFHARGNYDAAKRGP.G	N	59.47	4410.02	38	-2.5	736.0087	6	44.62	1.22E+06	6	8009	17041104.	4	30	67	
8	13993	P0DJI9|SAA2_HUMAN	R.SFFSFLGEAFD.G	N	55.75	1265.56	11	0	633.7875	2	79.66	1.21E+06	6	15513	17041104.	1	20	30	
8	13993	P0DJI9|SAA2_HUMAN	R.LTGRGAEDSLADQAANKWGRSGRDPNHFRPAGLPEKY	Y	54.74	4037	37	-0.6	673.8403	6	39.35	4.31E+06	6	7008	17041104.	4	86	122	
8	13993	P0DJI9|SAA2_HUMAN	S.FLGEAFDGARDMWRAYSDMREANYIGSDKYFHARGNYDAAKRGP.G	N	54.22	5074.341	44	-0.6	635.2996	8	54.1	2.86E+06	6	9927	17041104.	3	24	67	
8	13993	P0DJI9|SAA2_HUMAN	L.TGRGAEDSLADQAANKWGRSGRDPNHFRPAGLPEKY	Y	54.03	3923.916	36	0.5	561.567	7	38.28	3.05E+06	6	6806	17041104.	6	87	122	
8	13993	P0DJI9|SAA2_HUMAN	R.DPNHFRPAGLPEKY	N	50.78	1639.811	14	0.2	547.611	3	34.26	1.21E+05	6	6067	17041104.	1	109	122	
8	13993	P0DJI9|SAA2_HUMAN	T.GRGAEDSLADQAANKWGRSGRDPNHFRPAGLPEKY	Y	50.61	3822.869	35	0.6	765.5815	5	38.24	7.47E+05	6	6798	17041104.	2	88	122	
8	13993	P0DJI9|SAA2_HUMAN	L.TGRGAEDSLADQAANKWGRSGRDPNH.F	Y	50.26	2765.298	26	0.6	554.0672	5	29.77	4.26E+05	6	5245	17041104.	2	87	112	
8	13993	P0DJI9|SAA2_HUMAN	R.LTGRGAEDSLADQAANKWGRSGRDPNH.F	Y	49.72	2878.382	27	1.1	576.6843	5	32.33	6.39E+05	6	5714	17041104.	4	86	112	
8	13993	P0DJI9|SAA2_HUMAN	D.SLADQAANKWGRSGRDPNHFRPAGLPEKY	Y	49.04	3237.618	29	-1.2	648.5301	5	37.88	1.19E+06	6	6733	17041104.	2	94	122	
8	13993	P0DJI9|SAA2_HUMAN	F.SFLGEAFDGARDMWRAYSDMREANYIGSDKYFHARGNYDAAKRGP.G	N	44.25	5161.374	45	-1.6	646.1779	8	59.18	6.12E+05	6	10859	17041104.	1	23	67	

8	13993	P0DJI9|SAA2_HUMAN	G.RGAEDSLADQAANKWGRSGRDPNHFRPAGLPEKY	Y	37.43	3765.847	34	3.5	628.6507	6	38.42	2.82E+05	6	6833	17041104.	1	89	122		
8	13993	P0DJI9|SAA2_HUMAN	G.AEDSLADQAANKWGRSGRDPNH.F	Y	32.22	2394.106	22	0.2	479.8286	5	31.36	5.25E+04	6	5534	17041104.	1	91	112		
8	13993	P0DJI9|SAA2_HUMAN	R.GPGGAWAAEVI.S	N	30.82	1026.513	11	1.2	514.2646	2	54.41	1.68E+05	6	9982	17041104.	1	66	76		
8	13993	P0DJI9|SAA2_HUMAN	L.GEAFDGARDMWRAYSDMREANYIGSDKYFHARGNYDAAKRGP.G	N	30.79	4814.189	42	0.8	602.7814	8	49.33	2.63E+05	5	8921	17041103.	1	26	67		
8	13993	P0DJI9|SAA2_HUMAN	A.AEVISNARENIQRLTGRGAEDSLADQAANKWGRSGRDPNHFRPAGLPEKY	Y	30.19	5517.775	50	-0.9	614.0928	9	45.23	1.69E+06	6	8120	17041104.	1	73	122		
8	13993	P0DJI9|SAA2_HUMAN	A.DQAANKWGRSGRDPNHFRPAGLPEKY	Y	29.13	2966.465	26	0	495.4181	6	32.52	4.27E+05	6	5749	17041104.	1	97	122		
8	13993	P0DJI9|SAA2_HUMAN	A.EVISNARENIQRLTGRGAEDSLADQAANKWGRSGRDPNHFRPAGLPEKY	Y	28.6	5446.738	49	-0.9	681.8489	8	45.01		6	8081	17041104.	1	74	122		
8	13993	P0DJI9|SAA2_HUMAN	S.RSFFSFL.G	N	27.41	902.465	7	-3.1	452.2384	2	58.64	1.31E+05	6	10759	17041104.	1	19	25		
10	198	P62328|TYB4_HUMAN	M.S(+42.01)DKPDMAEIEKFDKSKL.K	Y	83.39	2021.998	17	0.8	675.0071	3	42.93	5.02E+05	6	7701	17041104.	4	2	18	Acetylatioı	S1:Acetylation (N-term):1000.00
10	198	P62328|TYB4_HUMAN	M.S(+42.01)DKPDMAEIEKFDKSKLKKTE.T	Y	79.71	2508.278	21	0.8	628.0773	4	34.92	2.27E+05	6	6187	17041104.	1	2	22	Acetylatioı	S1:Acetylation (N-term):1000.00
10	198	P62328|TYB4_HUMAN	T.Q(-17.03)EKNPLPSKETIEQEKQAGES	Y	74.17	2352.145	21	1	785.0562	3	32.15	2.41E+06	6	5681	17041104.	3	24	44	Pyro-glu fr	Q1:Pyro-glu from Q:1000.00
10	198	P62328|TYB4_HUMAN	M.S(+42.01)DKPDMAEIEKFDKSKLKKTETQEKNPLPSKETIEQEKQAGES	Y	73.74	4960.486	43	0.2	993.1047	5	37.39	2.79E+07	6	6642	17041104.	15	2	44	Acetylatioı	S1:Acetylation (N-term):1000.00
10	198	P62328|TYB4_HUMAN	M.S(+42.01)DKPDM(+15.99)AEIEKFDKSKLKKTETQEKNPLPSKETIEQEKQAGES	Y	48.89	4976.481	43	2.2	830.4226	6	34.97	4.92E+06	5	6247	17041103.	5	2	44	Acetylatioı	S1:Acetylation (N-term):1000.00;M6:Oxidation (M):1000.00
10	198	P62328|TYB4_HUMAN	M.S(+42.01)DKPDMAEIEKFDKSKLKKTETQE.K	Y	44.55	2866.427	24	0	574.2927	5	35.31	5.98E+05	6	6258	17041104.	1	2	25	Acetylatioı	S1:Acetylation (N-term):1000.00
10	198	P62328|TYB4_HUMAN	E.TQEKNPLPSKETIEQEKQAGES	Y	42.97	2470.219	22	-1.1	618.5613	4	28.77	1.07E+06	6	5064	17041104.	1	23	44		
10	198	P62328|TYB4_HUMAN	E.KNPLPSKETIEQEKQAGES	Y	33.95	2112.07	19	-0.7	705.0301	3	26.95	1.32E+05	6	4731	17041104.	1	26	44		
10	198	P62328|TYB4_HUMAN	K.TETQEKNPLPSKETIEQEKQAGES	Y	33.61	2700.309	24	1.2	676.0853	4	29.21	8.55E+05	6	5143	17041104.	1	21	44		
10	198	P62328|TYB4_HUMAN	Q.EKNPLPSKETIEQEKQAGES	Y	31.1	2241.112	20	0	561.2853	4	28.19	2.20E+05	6	4958	17041104.	1	25	44		
10	198	P62328|TYB4_HUMAN	K.NPLPSKETIEQEKQAGES	Y	28.05	1983.975	18	0	662.3322	3	29.63	2.31E+05	6	5219	17041104.	1	27	44		
24	31	P00738|HPT_HUMAN	R.ILGGHLDAKGSFPWQAKMVSH.H	Y	83.99	2278.168	21	-0.3	456.6407	5	42.76	8.67E+05	6	7669	17041104.	4	162	182		
24	31	P00738|HPT_HUMAN	R.ILGGHLDAKGSFPWQAKMVSHH.N	Y	78.09	2415.227	22	1.1	604.8147	4	39.36	5.92E+05	6	7010	17041104.	1	162	183		
24	31	P00738|HPT_HUMAN	V.KVTSIQDWVQKTIAEN	Y	68.37	1858.979	16	0.5	620.6672	3	51.72	1.48E+06	6	9487	17041104.	2	391	406		
24	31	P00738|HPT_HUMAN	R.ILGGHLDAKGSFPWQAKMV.S	Y	65.6	2054.077	19	1	514.5271	4	47.83	1.20E+06	6	8661	17041104.	1	162	180		
24	31	P00738|HPT_HUMAN	R.ILGGHLDAKGSFPWQ.A	Y	59.56	1624.836	15	0.7	542.6197	3	48.26	2.46E+06	5	8727	17041103.	3	162	176		
24	31	P00738|HPT_HUMAN	G.AVIALLLWGQ.L	Y	48.17	1082.649	10	0.2	542.3318	2	74.68	1.91E+06	6	14290	17041104.	1	6	15		
24	31	P00738|HPT_HUMAN	L.GAVIALLLWGQ.L	Y	46.79	1139.67	11	0.9	570.843	2	76.59	6.15E+05	5	14087	17041103.	3	5	15		
16	14011	P69905|HBA_HUMAN	L.VTLAAHLPAEFTPAVHASLDKFLASVSTVLTSKYR	Y	77.82	3740.03	35	3	749.0155	5	67.69	1.33E+06	5	12359	17041103.	3	108	142		
16	14011	P69905|HBA_HUMAN	T.LAAHLPAEFTPAVHASLDKFLASVSTVLTSKYR	Y	70.58	3539.914	33	-0.5	708.9897	5	66.35	4.12E+05	5	12109	17041103.	1	110	142		
16	14011	P69905|HBA_HUMAN	M.VLSPADKTNVKAAWGKVGAHAGEYGAEALERM.F	Y	65.96	3325.688	32	1.4	832.4304	4	46.75	6.77E+05	5	8440	17041103.	2	2	33		
16	14011	P69905|HBA_HUMAN	M.VLSPADKTNVKAAWGKVGAHAGEYGAEALERMF.L	Y	63.87	3472.756	33	1.3	695.5594	5	53.51	1.77E+06	5	9705	17041103.	6	2	34		
16	14011	P69905|HBA_HUMAN	D.KFLASVSTVLTSKYR	Y	62.73	1698.967	15	5.5	567.3326	3	41.68	7.42E+04	5	7497	17041103.	1	128	142		
16	14011	P69905|HBA_HUMAN	L.AAHLPAEFTPAVHASLDKFLASVSTVLTSKYR	Y	59.36	3426.83	32	-0.5	686.3729	5	65.09	1.24E+06	5	11878	17041103.	1	111	142		
16	14011	P69905|HBA_HUMAN	M.VLSPADKTNVKAAWGKVGAHAGEYGAEALERMFLSF.P	Y	39.6	3819.94	36	-3.2	764.9929	5	62.76	1.85E+05	5	11435	17041103.	1	2	37		
16	14011	P69905|HBA_HUMAN	M.VLSPADKTNVKAAWGKVG.A	Y	31.57	1840.021	18	1.5	614.3484	3	34.74	2.97E+05	5	6202	17041103.	1	2	19		
26	14014	O95810|SDPR_HUMAN	R.YEGSYALTSEEAERSDGDPVQPAVLQVHQTS	Y	87.88	3362.554	31	-0.8	1121.858	3	46.23	2.61E+06	6	8314	17041104.	1	395	425		
26	14014	O95810|SDPR_HUMAN	M.G(+42.01)EDAAQAEKFQHPGSDMR.Q	Y	76.9	2014.88	18	0.6	672.6345	3	33.24	3.94E+05	6	5881	17041104.	2	2	19	Acetylatioı	G1:Acetylation (N-term):1000.00
26	14014	O95810|SDPR_HUMAN	Y.ALTSEEAERSDGDPVQPAVLQVHQTS	Y	75.47	2763.331	26	0.4	922.118	3	40.33	1.49E+06	6	7204	17041104.	2	400	425		
26	14014	O95810|SDPR_HUMAN	M.G(+42.01)EDAAQAEKFQHPG.S	Y	70.1	1525.68	14	-0.4	763.8468	2	32.87	5.75E+05	6	5814	17041104.	3	2	15	Acetylatioı	G1:Acetylation (N-term):1000.00
26	14014	O95810|SDPR_HUMAN	M.G(+42.01)EDAAQAEKFQHPGSDM.R	Y	59.92	1858.779	17	-2.2	930.3948	2	38.06	2.04E+05	6	6765	17041104.	1	2	18	Acetylatioı	G1:Acetylation (N-term):1000.00
26	14014	O95810|SDPR_HUMAN	G.SYALTSEEAERSDGDPVQPAVLQVHQTS	Y	53.4	3013.427	28	1.2	1005.484	3	44.03	6.06E+05	6	7900	17041104.	1	398	425		
26	14014	O95810|SDPR_HUMAN	M.G(+42.01)EDAAQAEKFQHPGSDM(+15.99)R.Q	Y	32.04	2030.875	18	1.9	677.9669	3	30.32	7.27E+04	6	5346	17041104.	1	2	19	Acetylatioı	G1:Acetylation (N-term):1000.00;M17:Oxidation (M):1000.00
11	15	P02765|FETUA_HUMAN	G.APGLPPAGSPPDSHVLL.A	Y	75.29	1623.862	17	-0.3	812.938	2	48.5	7.19E+06	5	8770	17041103.	3	285	301		
11	15	P02765|FETUA_HUMAN	R.TVVQPSVGAAAGPVVPPCPGRIRHFKV	Y	68.75	2738.517	27	0.6	548.7109	5	42.45	6.03E+06	6	7613	17041104.	6	341	367		
11	15	P02765|FETUA_HUMAN	G.APGLPPAGSPPDSHVLLA.A	Y	65.68	1694.899	18	0.6	848.4573	2	48.2	7.44E+05	5	8715	17041103.	2	285	302		
11	15	P02765|FETUA_HUMAN	L.AAPPGHQLHRAHYDLRHTFMGVVSLGSPSGEVSHPRKT.R	Y	59.12	4129.093	38	1.4	590.8785	7	38.72	1.93E+07	5	6942	17041103.	8	302	339		
11	15	P02765|FETUA_HUMAN	G.APGLPPAGSPPDSHVLLAAPPGHQLHRAHYDLRHTFMGVVSLGSPSGEVSHPRKT.R	Y	55.69	5734.944	55	0.4	820.2853	7	46.13	6.71E+06	5	8325	17041103.	9	285	339		
11	15	P02765|FETUA_HUMAN	A.APPGHQLHRAHYDLRHTFMGVVSLGSPSGEVSHPRKT.R	Y	53.5	4058.056	37	0.4	508.2644	8	38.56	1.36E+06	5	6913	17041103.	5	303	339		
11	15	P02765|FETUA_HUMAN	G.APGLPPAGSPPDSHVL.L	Y	42.02	1510.778	16	2.4	756.3981	2	42.91	4.91E+05	5	7732	17041103.	2	285	300		
11	15	P02765|FETUA_HUMAN	L.AAPPGHQLHRAHYDLRHTFM(+15.99)GVVSLGSPSGEVSHPRKT.R	Y	41.63	4145.088	38	-1.9	691.8539	6	36.21	9.67E+05	5	6475	17041103.	1	302	339	Oxidation	M20:Oxidation (M):1000.00
11	15	P02765|FETUA_HUMAN	G.APGLPPAGSPPDSHVLLAAPPGHQLHRAHYDLRHTFM(+15.99)GVVSLGSPSGEVSHPRKT.R	Y	36.01	5750.939	55	3.2	719.877	8	44.72	1.90E+06	5	8064	17041103.	1	285	339	Oxidation	M37:Oxidation (M):1000.00
11	15	P02765|FETUA_HUMAN	L.AAPPGHQLHRAHYDLRHTFMGVVSLGSPSGEVSHPR.K	Y	27.99	3899.95	36	2.9	558.1447	7	40.82	7.75E+04	5	7339	17041103.	1	302	337		
22	13999	P10645|CMGA_HUMAN	K.HSGFEDELSEVLENQSSQAELKEAVEEPSSKDVM.E	Y	94.65	3776.721	34	1.2	945.1887	4	58.51	2.95E+06	6	10736	17041104.	1	97	130		
22	13999	P10645|CMGA_HUMAN	K.HSGFEDELSEVLENQSSQAELKEAVEEPSSKDVME.K	Y	85.6	3905.764	35	-1.7	977.4465	4	58.62	1.33E+07	6	10756	17041104.	4	97	131		
22	13999	P10645|CMGA_HUMAN	R.GYPEEKKEEEGSANRRPEDQELESLSAIEAELEKVAHQLQALRR(-.98).G	Y	57.99	5060.555	44	2.4	633.5782	8	60.53	4.88E+06	6	11114	17041104.	4	413	456	Amidation	R44:Amidation:1000.00
22	13999	P10645|CMGA_HUMAN	K.HSGFEDELSEVLENQSSQAELKEAVEEPSSKDVM(+15.99).E	Y	50.39	3792.716	34	-4.1	949.1824	4	56.39	4.07E+05	6	10345	17041104.	1	97	130	Oxidation	M34:Oxidation (M):1000.00
22	13999	P10645|CMGA_HUMAN	R.LEGQEEEEDNRDSSMKLSFRARAYGFRGPGPQL.R	Y	48.5	3768.791	33	-1.7	754.7643	5	42.74	1.04E+06	6	7667	17041104.	2	358	390		
22	13999	P10645|CMGA_HUMAN	R.LEGQEEEEDNRDSSMKLSF.R	Y	38.49	2241.97	19	1.2	748.3314	3	40.88	1.98E+05	6	7307	17041104.	1	358	376		
20	111	tr|A0A087WXL8|A0A087WXL8_HUMAN	T.VSWNSGALTSGVHTFPAVLQ.S	Y	81.37	2070.054	20	0.8	1036.035	2	58.5	6.29E+06	5	10641	17041103.	4	179	198		
20	111	tr|A0A087WXL8|A0A087WXL8_HUMAN	N.SGALTSGVHTFPAVLQ.S	Y	55.93	1583.831	16	0.9	792.9233	2	49.12	2.96E+05	5	8883	17041103.	1	183	198		
20	111	tr|A0A087WXL8|A0A087WXL8_HUMAN	T.VSWNSGALTSGVHTFPAVL.Q	Y	54.56	1941.995	19	0.2	972.0049	2	60.1	3.63E+05	5	10942	17041103.	1	179	197		
20	111	tr|A0A087WXL8|A0A087WXL8_HUMAN	T.SGVHTFPAVLQ.S	Y	52.22	1154.608	11	0.1	578.3115	2	42.63	1.31E+07	5	7681	17041103.	3	188	198		
20	111	tr|A0A087WXL8|A0A087WXL8_HUMAN	S.Q(-17.03)VQLVQSGAEVK.K	Y	49.58	1267.677	12	0.7	634.8463	2	44.43	5.24E+04	5	8012	17041103.	1	20	31	Pyro-glu fr	Q1:Pyro-glu from Q:1000.00
20	111	tr|A0A087WXL8|A0A087WXL8_HUMAN	G.GPSVFLFPPKPKDTLMIS.R	Y	48.1	1973.07	18	-0.5	658.6968	3	55.11	7.62E+05	5	10007	17041103.	3	307	324		
20	111	tr|A0A087WXL8|A0A087WXL8_HUMAN	G.GPSVFLFPPKPKDTLM(+15.99).I	Y	42.02	1788.948	16	1.7	597.3244	3	48.21	1.75E+05	5	8717	17041103.	1	307	322	Oxidation	M16:Oxidation (M):1000.00
20	111	tr|A0A087WXL8|A0A087WXL8_HUMAN	T.SGVHTFPAVL.Q	Y	41.36	1026.55	10	1.4	514.2829	2	44.45	6.85E+05	5	8015	17041103.	2	188	197		
20	111	tr|A0A087WXL8|A0A087WXL8_HUMAN	T.SGVHTFPAVLQSSGLYS.L	Y	40.83	1748.873	17	2.3	875.4459	2	51.88	1.10E+05	5	9401	17041103.	1	188	204		
20	111	tr|A0A087WXL8|A0A087WXL8_HUMAN	G.GPSVFL.F	Y	30.92	618.3377	6	1.7	619.346	1	47.56	7.80E+05	5	8595	17041103.	1	307	312		
13	27	P01024|CO3_HUMAN	R.SSKITHRIHWESASLLR.S	Y	78.41	2020.097	17	0.6	506.0317	4	32.92	6.57E+06	6	5822	17041104.	7	1304	1320		
13	27	P01024|CO3_HUMAN	R.SSKITHRIHWESASLL.R	Y	61.12	1863.996	16	0.5	467.0064	4	36.2	5.75E+06	6	6420	17041104.	5	1304	1319		
13	27	P01024|CO3_HUMAN	I.THRIHWESASLL.R	Y	58.48	1448.752	12	1	483.9253	3	37.39	2.51E+06	6	6643	17041104.	2	1308	1319		
13	27	P01024|CO3_HUMAN	S.KITHRIHWESASLL.R	Y	57.7	1689.931	14	0.1	423.4902	4	36.14	8.96E+05	6	6409	17041104.	2	1306	1319		
13	27	P01024|CO3_HUMAN	G.SPMYSIITPNILRLESEETMVLEAHDAQ.G	Y	54.74	3186.558	28	2.8	1063.196	3	65.96	5.66E+05	5	12037	17041103.	1	23	50		
13	27	P01024|CO3_HUMAN	H.RIHWESASLL.R	Y	51.18	1210.646	10	0.4	606.3304	2	42.81	2.66E+05	6	7679	17041104.	1	1310	1319		
13	27	P01024|CO3_HUMAN	T.HRIHWESASLL.R	Y	46.43	1347.705	11	-0.7	450.2419	3	37.93	7.32E+05	6	6741	17041104.	2	1309	1319		
13	27	P01024|CO3_HUMAN	I.HWESASLL.R	Y	42.97	941.4607	8	0.7	471.7379	2	42.02	3.86E+07	6	7533	17041104.	10	1312	1319		
13	27	P01024|CO3_HUMAN	R.IHWESASLL.R	Y	29.71	1054.545	9	2.1	528.2807	2	49.21	8.79E+04	5	8899	17041103.	1	1311	1319		
23	14003	P04004|VTNC_HUMAN	R.TSAGTRQPQFISRDWHGVPGQVDAAMAGRIYISGMAPRPSLA.K	Y	84.48	4452.233	42	-9.9	891.4451	5	52.56		6	9643	17041104.	3	325	366		
23	14003	P04004|VTNC_HUMAN	G.TRQPQFISRDWHGVPGQVDAAMAGRIYIS.G	Y	84	3255.636	29	-0.8	652.134	5	49.96	1.26E+06	6	9151	17041104.	4	329	357		
23	14003	P04004|VTNC_HUMAN	D.LQAQSKGNPEQTPVLKPEEEAPAPEVGASKPEG.I	Y	62.33	3411.716	33	1.9	853.9378	4	35.3	3.98E+05	6	6256	17041104.	2	102	134		
23	14003	P04004|VTNC_HUMAN	L.FWGRTSAGTRQPQFISRDWHGVPGQVDAAMAGRIYISGMAPRPSLA.K	Y	48.1	4998.503	46	5.1	834.0955	6	53.8	2.78E+05	5	9759	17041103.	2	321	366		
23	14003	P04004|VTNC_HUMAN	G.TRQPQFISRDWHGVPGQVDAAMAGRIYISGMAPRP.S	Y	45.52	3864.942	35	0.8	645.1647	6	51.11		5	9256	17041103.	1	329	363		
23	14003	P04004|VTNC_HUMAN	R.TSAGTRQPQFISRDWHGVPGQVDAAMAGRIYISGM(+15.99)APRPSLA.K	Y	43.4	4468.228	42	0.6	745.7124	6	50.52	9.75E+04	5	9143	17041103.	2	325	366	Oxidation	M35:Oxidation (M):9.99
23	14003	P04004|VTNC_HUMAN	L.FWGRTSAGTRQPQFISRDWHGVPGQVDAAMAGRIYISGMAPRPS.L	Y	38.62	4814.382	44	-3.5	803.4015	6	51.93	1.54E+05	5	9410	17041103.	1	321	364		
17	28	tr|Q9BS19|Q9BS19_HUMAN	N.SVFLIKGDKVWVYPPEKKEKGYPKLLQ.D	Y	78.88	3188.8	27	-0.4	638.767	5	44.48	2.46E+06	5	8021	17041103.	3	107	133		
17	28	tr|Q9BS19|Q9BS19_HUMAN	N.SVFLIKGDKVWVYPPEK.K	Y	75.19	2004.108	17	0.2	669.0435	3	46.68	7.30E+06	6	8402	17041104.	4	107	123		
17	28	tr|Q9BS19|Q9BS19_HUMAN	N.SVFLIKGDKVWVYPPEKKE.K	Y	71.13	2261.246	19	0.9	566.3193	4	42.38	3.15E+06	6	7599	17041104.	10	107	125		

17	28	tr|Q9BS19|Q9BS19_HUMAN	N.FPSPVDAAFRQGHN.S	Y	50.74	1541.737	14	1.4	514.9205	3	40.52	5.85E+05	6	7239	17041104.	3	93	106		
17	28	tr|Q9BS19|Q9BS19_HUMAN	N.SVFLIKGDKVWVYPPE.K	Y	47.31	1876.013	16	0.1	626.3452	3	52.71	1.46E+06	6	9671	17041104.	3	107	122		
17	28	tr|Q9BS19|Q9BS19_HUMAN	W.VYPPEKKEKGYPKLLQ.D	Y	29.74	1916.077	16	2.5	480.0277	4	29.21	1.93E+05	5	5167	17041103.	1	118	133		
17	29	P02790|HEMO_HUMAN	N.SVFLIKGDKVWVYPPEKKEKGYPKLLQ.D	Y	78.88	3188.8	27	-0.4	638.767	5	44.48	2.46E+06	5	8021	17041103.	3	107	133		
17	29	P02790|HEMO_HUMAN	N.SVFLIKGDKVWVYPPEK.K	Y	75.19	2004.108	17	0.2	669.0435	3	46.68	7.30E+06	6	8402	17041104.	4	107	123		
17	29	P02790|HEMO_HUMAN	N.SVFLIKGDKVWVYPPEKKE.K	Y	71.13	2261.246	19	0.9	566.3193	4	42.38	3.15E+06	6	7599	17041104.	10	107	125		
17	29	P02790|HEMO_HUMAN	N.FPSPVDAAFRQGHN.S	Y	50.74	1541.737	14	1.4	514.9205	3	40.52	5.85E+05	6	7239	17041104.	3	93	106		
17	29	P02790|HEMO_HUMAN	N.SVFLIKGDKVWVYPPE.K	Y	47.31	1876.013	16	0.1	626.3452	3	52.71	1.46E+06	6	9671	17041104.	3	107	122		
17	29	P02790|HEMO_HUMAN	W.VYPPEKKEKGYPKLLQ.D	Y	29.74	1916.077	16	2.5	480.0277	4	29.21	1.93E+05	5	5167	17041103.	1	118	133		
29	13996	Q15942|ZYX_HUMAN	F.HVQPQPQPKPQVQLHVQSQT.Q	Y	81.23	2303.213	20	0.4	768.7454	3	28.72	3.90E+05	6	5054	17041104.	2	223	242		
29	13996	Q15942|ZYX_HUMAN	S.LANTQPRGPPASSPAPAPKFSPVTPKFTPVAS.K	Y	70.72	3214.714	32	-0.6	804.6852	4	42.53	8.30E+05	6	7627	17041104.	1	247	278		
29	13996	Q15942|ZYX_HUMAN	F.HVQPQPQPKPQVQLHVQ.S	Y	55.42	1987.075	17	1.1	663.3664	3	28.58	8.16E+04	6	5028	17041104.	2	223	239		
29	13996	Q15942|ZYX_HUMAN	M.A(+42.01)APRPSPAISVSVSAPAF.Y	Y	54.29	1765.936	18	3.3	883.9783	2	58.41	7.54E+05	6	10717	17041104.	2	2	19	Acetylatioı	A1:Acetylation (N-term):1000.00
29	13996	Q15942|ZYX_HUMAN	F.HVQPQPQPKPQVQLHVQSQTQPVS.L	Y	34.19	2714.425	24	-1.2	679.6128	4	31.35	8.07E+04	6	5533	17041104.	1	223	246		
29	13996	Q15942|ZYX_HUMAN	K.VNPFRPGDSEPPPAPGAQRAQ.M	Y	31.44	2187.082	21	0.7	730.0351	3	33.99	8.52E+04	6	6017	17041104.	1	36	56		
27	25	P02656|APOC3_HUMAN	A.SEAEDASLLSFMQGYMKHATKTAKD.A	Y	83.49	2758.294	25	1	690.5815	4	52.72	2.20E+06	6	9673	17041104.	3	21	45		
27	25	P02656|APOC3_HUMAN	A.SEAEDASLLSFMQGYMKHATKTA.K	Y	60.95	2515.172	23	1	629.801	4	56.83	2.76E+05	6	10425	17041104.	1	21	43		
27	25	P02656|APOC3_HUMAN	F.WDLDPEVRPTSAVAA	Y	56.6	1625.805	15	-0.5	813.9093	2	49.64	5.81E+05	6	9085	17041104.	1	85	99		
27	25	P02656|APOC3_HUMAN	A.SEAEDASLLSFMQGYMKHATKTAKDALSSVQESQVAQQA.R	Y	40.56	4185	39	-1.9	1047.255	4	63.84		6	11757	17041104.	1	21	59		
27	25	P02656|APOC3_HUMAN	D.PEVRPTSAVAA	Y	40.41	1096.588	11	1.3	549.3018	2	27.92	2.27E+06	6	4909	17041104.	3	89	99		
25	14012	P13521|SCG2_HUMAN	P.VGPPKNDDTPNRQYWDEDLLMKVLEYLNQEKAEKGREHIA.K	Y	101.25	4738.345	40	1.5	790.7326	6	57.85	7.80E+06	6	10614	17041104.	7	571	610		
25	14012	P13521|SCG2_HUMAN	R.FPVGPPKNDDTPNRQYWDEDLLMKVLEYLNQEKAEKGREHIA.K	Y	51.7	4982.466	42	-2.8	712.7861	7	59.22	1.51E+06	6	10866	17041104.	1	569	610		
25	14012	P13521|SCG2_HUMAN	P.VGPPKNDDTPNRQYWDEDLLMKVLEYLNQEKAEKGREH.I	Y	51.17	4554.224	38	2.4	760.0464	6	57.52	4.85E+05	6	10552	17041104.	1	571	608		
25	14012	P13521|SCG2_HUMAN	P.VGPPKNDDTPNRQYWDEDLLM(+15.99)KVLEYLNQEKAEKGREHIA.K	Y	34.14	4754.34	40	-3.1	680.1966	7	56.53	4.58E+05	6	10371	17041104.	1	571	610	Oxidation	M21:Oxidation (M):1000.00
25	14012	P13521|SCG2_HUMAN	P.GQGSSEDDLQEEEQIEQAIKEHLNQGSSQETDKLAPVS.K	Y	29.88	4152.92	38	-1.1	1039.236	4	55.5	4.45E+06	6	10182	17041104.	2	529	566		
25	14012	P13521|SCG2_HUMAN	R.VPGQGSSEDDLQEEEQIEQAIKEHLNQGSSQETDKLAPVS.K	Y	15.77	4349.042	40	0.8	1088.269	4	56.26	7.61E+05	6	10321	17041104.	1	527	566		
25	14012	P13521|SCG2_HUMAN	P.G(+42.01)RAGTEALPDGLS.V	Y	9.32	1284.631	13	-9.2	643.3168	2	66.48		5	12132	17041103.	1	420	432	Acetylatioı	G1:Acetylation (N-term):1000.00
25	14012	P13521|SCG2_HUMAN	L.KHMQFPPMYE.E	Y	5.13	1306.584	10	-6.3	1307.583	1	82.53		5	15184	17041103.	1	162	171		
39	99	P01019|ANGT_HUMAN	N.KPEVLEVTLNRPFLFAVYDQSATALHFLGRVANPLSTA	Y	88.66	4184.242	38	3.1	1047.071	4	71.87	1.94E+07	6	13690	17041104.	6	448	485		
39	99	P01019|ANGT_HUMAN	E.STQQLNKPEVLEVTLNRPFLFAVYDQSATALHFLGRVANPLSTA	Y	76.43	4855.566	44	2	972.1224	5	72.53	7.01E+05	6	13840	17041104.	1	442	485		
39	99	P01019|ANGT_HUMAN	T.Q(-17.03)QLNKPEVLEVTLNRPFLFAVYDQSATALHFLGRVANPLSTA	Y	42.31	4650.46	42	-1.4	1163.621	4	77.34		6	14903	17041104.	1	444	485	Pyro-glu fr	Q1:Pyro-glu from Q:1000.00
106	14095	P02741|CRP_HUMAN	G.Q(-17.03)TDMSRKAFVFPKESDTSYVSLK.A	Y	95.51	2646.3	23	-0.1	662.5822	4	45.83	7.29E+05	5	8267	17041103.	1	19	41	Pyro-glu fr	Q1:Pyro-glu from Q:1000.00
106	14095	P02741|CRP_HUMAN	G.Q(-17.03)TDMSRKAFVFPKESDTSYVSLKAPLT.K	Y	53.13	3028.522	27	-0.9	758.137	4	51.32	1.09E+05	5	9294	17041103.	1	19	45	Pyro-glu fr	Q1:Pyro-glu from Q:1000.00
106	14095	P02741|CRP_HUMAN	G.Q(-17.03)TDMSRKAFVFPKESDTSYVS.L	Y	47.71	2405.121	21	0.2	802.7144	3	45.57	2.22E+06	5	8219	17041103.	1	19	39	Pyro-glu fr	Q1:Pyro-glu from Q:1000.00
28	85	P02768|ALBU_HUMAN	R.DAHKSEVAHRFKDLGEENFKALVLIAFAQYLQ.Q	Y	73.1	3686.921	32	-1.5	738.3903	5	65.79	4.42E+06	5	12007	17041103.	4	25	56		
28	85	P02768|ALBU_HUMAN	R.DAHKSEVAHRFKDLGEENFK.A	Y	64.77	2356.156	20	1.8	590.0474	4	30.31	3.03E+05	5	5371	17041103.	3	25	44		
28	85	P02768|ALBU_HUMAN	R.DAHKSEVAHRFKDLGEENFKALVL.I	Y	58.58	2752.43	24	0.9	551.4937	5	44.73	2.13E+06	5	8065	17041103.	3	25	48		
40	14096	P37802|TAGL2_HUMAN	M.GTNRGASQAGMTGYGMPRQIL	Y	74.74	2165.047	21	0.6	722.69	3	40.92	2.46E+06	6	7315	17041104.	2	179	199		
40	14096	P37802|TAGL2_HUMAN	Q.MGTNRGASQAGMTGYGMPRQIL	Y	71.74	2296.087	22	0.5	766.3701	3	42.51	4.20E+05	6	7624	17041104.	2	178	199		
40	14096	P37802|TAGL2_HUMAN	M.GTNRGASQAGM(+15.99)TGYGMPRQIL	Y	70.02	2181.042	21	2.7	728.0233	3	37.45	3.02E+05	6	6653	17041104.	2	179	199	Oxidation	M11:Oxidation (M):68.60
40	14096	P37802|TAGL2_HUMAN	M.TGYGMPRQIL	Y	52.42	1134.586	10	0.3	568.3002	2	42.56	1.88E+05	5	7667	17041103.	1	190	199		
40	14096	P37802|TAGL2_HUMAN	M.GTNRGASQAGMTGYGM(+15.99)PRQIL	Y	44.09	2181.042	21	-3.2	728.019	3	35.87	5.06E+04	5	6412	17041103.	1	179	199	Oxidation	M16:Oxidation (M):68.60
80	14016	P01298|PAHO_HUMAN	R.HKEDTLAFSEWGSPHAAVPR.E	Y	73.19	2234.087	20	0.9	559.5295	4	38.07	3.83E+05	6	6766	17041104.	1	69	88		
80	14016	P01298|PAHO_HUMAN	P.LEPVYPGDNATPEQMAQYAADLRRYINMLTRPRY(-.98).G	Y	66.36	4010.988	34	-5.1	803.2008	5	64.87	2.23E+06	6	11996	17041104.	3	32	65	Amidation	Y34:Amidation:1000.00
80	14016	P01298|PAHO_HUMAN	P.LEPVYPGDNATPEQMAQYAADLRRY.I	Y	23.19	2867.355	25	-2.8	956.7896	3	56.42	1.86E+05	6	10351	17041104.	1	32	56		
80	14016	P01298|PAHO_HUMAN	G.AQGAPLEPVYPGDNATPEQMAQYAADLRRYINMLTRPRY(-.98).G	Y	14.37	4435.195	39	-3.8	888.043	5	65.35		5	11924	17041103.	1	27	65	Amidation	Y39:Amidation:1000.00
80	14016	P01298|PAHO_HUMAN	P.LEPVYPGDNATPEQMAQYAADLRRYINMLTRPRY.G	Y	9.05	4011.972	34	7.7	1004.008	4	65.54	8.94E+04	5	11959	17041103.	1	32	65		
31	194	O15240|VGF_HUMAN	P.GRPEAQPPPLSSEHKEPVAGDAVPGPKDGSAPEVRGA.R	Y	59.32	3685.845	37	0.4	615.315	6	32.46	2.27E+06	6	5737	17041104.	4	26	62		
31	194	O15240|VGF_HUMAN	P.GRPEAQPPPLSSEHKEPVAGDAVPGPKDGSAPEV.R	Y	47.5	3401.685	34	1.1	681.345	5	35.11	4.77E+05	6	6222	17041104.	1	26	59		
31	194	O15240|VGF_HUMAN	A.APPGRPEAQPPPLSSEHKEPVAGDAVPGPKDGSAPEVRGA.R	Y	47.06	3950.987	40	2.1	659.5065	6	34.09	2.46E+06	6	6036	17041104.	6	23	62		
31	194	O15240|VGF_HUMAN	N.APPEPVPPPRAAPAPTHV.R	Y	33.39	1799.968	18	0.2	600.9968	3	33.75	6.21E+04	6	5974	17041104.	1	486	503		
31	194	O15240|VGF_HUMAN	K.NAPPEPVPPPRAAPAPTHV.R	Y	25.51	1914.011	19	1	639.0116	3	34.2	2.96E+05	5	6102	17041103.	3	485	503		
31	194	O15240|VGF_HUMAN	A.APPGRPEAQPPPLSSEHKEPVAGDAVPGPKDGSAPEV.R	Y	11.59	3666.828	37	-1.4	734.3718	5	36.22	4.80E+05	6	6423	17041104.	1	23	59		
31	194	O15240|VGF_HUMAN	A.AEALLTET.V	Y	11.48	846.4335	8	4.5	847.4446	1	82.77	3.38E+04	5	15229	17041103.	1	111	118		
31	194	O15240|VGF_HUMAN	K.NAPPEPVPPPRAAPAPTHVRSPQPPPPAPAPARDELPD.W	Y	5.42	3903.018	38	-0.6	781.6104	5	37.8	1.98E+05	6	6718	17041104.	1	485	522		
125	2831	P00450|CERU_HUMAN	C.HVTDHIHAGMETTYTVLQNEDTKSG	Y	74.43	2783.282	25	1.3	696.8287	4	39.3	2.18E+05	6	6999	17041104.	1	1041	1065		
125	2831	P00450|CERU_HUMAN	A.KEKHYYIGIIETTWDYA.S	Y	60.68	2129.047	17	-0.5	710.6892	3	52.31	8.45E+05	6	9596	17041104.	1	20	36		
54	13998	O00151|PDLI1_HUMAN	A.SPASSTTARVITNQY.N	Y	69.27	1594.795	15	2.3	798.4066	2	34.21	7.03E+04	6	6058	17041104.	2	130	144		
54	13998	O00151|PDLI1_HUMAN	Q.EILESEEKGDPNKPSGFRSVKAPVT.K	Y	45.26	2713.392	25	2.4	679.3569	4	33.74	1.01E+05	6	5973	17041104.	1	221	245		
54	13998	O00151|PDLI1_HUMAN	Y.NNPAGLYSSENISNFNNALESKTAASGVE.A	Y	38.02	2997.395	29	5.2	1000.144	3	52.57	1.34E+05	6	9645	17041104.	1	145	173		
151	14023	P68366|TBA4A_HUMAN	A.TYAPVISAEKAYHEQ.L	Y	68.43	1705.831	15	2.6	569.6191	3	35.74	1.71E+05	6	6336	17041104.	1	271	285		
151	14023	P68366|TBA4A_HUMAN	A.TYAPVISAEKAYHEQL.S	Y	57.8	1818.915	16	0.1	607.3124	3	42.21	5.73E+05	6	7568	17041104.	1	271	286		
151	14023	P68366|TBA4A_HUMAN	S.AEKAYHEQLSVAEITN.A	Y	39.01	1801.885	16	0.7	601.6359	3	35.72	8.49E+04	6	6332	17041104.	1	278	293		
100	14020	tr|F8W6P5|F8W6P5_HUMAN	M.VHLTPEEKSAVTALWGKVNVDEVGGEALGRL.L	Y	65.46	3273.736	31	-0.2	655.7543	5	57.85	2.52E+05	5	10520	17041103.	1	2	32		
100	14020	tr|F8W6P5|F8W6P5_HUMAN	M.VHLTPEEKSAVTALWGKVNVDEVGGEALGRLLVVYPWTQRF.F	Y	59.77	4563.428	41	4.2	913.6967	5	68.01	3.18E+05	5	12418	17041103.	2	2	42		
100	14020	tr|F8W6P5|F8W6P5_HUMAN	L.LVVYPWTQRF.F	Y	43	1307.703	10	-0.3	654.8584	2	54.34	3.72E+05	5	9862	17041103.	1	33	42		
100	14021	P68871|HBB_HUMAN	M.VHLTPEEKSAVTALWGKVNVDEVGGEALGRL.L	Y	65.46	3273.736	31	-0.2	655.7543	5	57.85	2.52E+05	5	10520	17041103.	1	2	32		
100	14021	P68871|HBB_HUMAN	M.VHLTPEEKSAVTALWGKVNVDEVGGEALGRLLVVYPWTQRF.F	Y	59.77	4563.428	41	4.2	913.6967	5	68.01	3.18E+05	5	12418	17041103.	2	2	42		
100	14021	P68871|HBB_HUMAN	L.LVVYPWTQRF.F	Y	43	1307.703	10	-0.3	654.8584	2	54.34	3.72E+05	5	9862	17041103.	1	33	42		
44	14077	Q13201|MMRN1_HUMAN	T.VGGTGGIGGVGGTGGVGNRAPR.E	Y	58.67	1851.966	22	-0.4	618.3291	3	29.51	8.70E+05	6	5198	17041104.	2	159	180		
44	14077	Q13201|MMRN1_HUMAN	V.GGTGGIGGVGGTGGVGNRAPR.E	Y	43.94	1752.898	21	-1.5	585.3057	3	27.7	1.22E+05	5	4883	17041103.	2	160	180		
44	14077	Q13201|MMRN1_HUMAN	T.VGGTGGIGGVGGTGGVGNRAP.R	Y	40.67	1695.865	21	0.3	848.9402	2	34.57	3.64E+04	6	6123	17041104.	1	159	179		
44	14077	Q13201|MMRN1_HUMAN	N.TVGGTGGIGGVGGTGGVGNRAPR.E	Y	37.89	1953.014	23	-0.2	652.0118	3	30.55	1.16E+05	6	5387	17041104.	1	158	180		
134	72	P19823|ITIH2_HUMAN	G.FEIPINGLSEFVDYEDLVELAPGKFQLVAENRRYQ.R	Y	93.06	4098.074	35	-1.1	1025.525	4	73.4	1.13E+07	6	14025	17041104.	2	19	53		
134	72	P19823|ITIH2_HUMAN	R.SILQMSLDHHIVTPLTSLVIENEAGDERMLA.D	Y	29.26	3431.743	31	-2.5	858.9409	4	64.47		5	11758	17041103.	1	611	641		
261	14018	P10909|CLUS_HUMAN	V.ASHTSDSDVPSGVTEVVVKLFDSDPITVTVPVEV.S	Y	79.7	3524.777	34	-0.2	1175.933	3	75.83	2.72E+06	6	14559	17041104.	1	390	423		
261	14018	P10909|CLUS_HUMAN	K.FMETVAEKALQEYRKKHREE	Y	53.57	2521.275	20	1	505.2627	5	37.44	6.17E+05	6	6652	17041104.	1	430	449		
3	115	P02654|APOC1_HUMAN	G.TPDVSSALDKLKEFGNTLEDKARELISRIKQSELSAKMREWFSETFQKVKEKLKIDS	Y	72.77	6626.506	57	0.6	829.321	8	75.29	1.64E+08	5	13803	17041103.	117	27	83		
3	115	P02654|APOC1_HUMAN	P.DVSSALDKLKEFGNTLEDKARELISRIKQSELSAKMREWFSETFQKVKEKLKIDS	Y	66.26	6428.405	55	1.4	804.559	8	74.67	4.46E+08	6	14288	17041104.	42	29	83		
3	115	P02654|APOC1_HUMAN	G.TPDVSSALDKLKEFGNTLEDKARELISRIKQSELSAKM(+15.99)REWFSETFQKVKEKLKIDS	Y	57.48	6642.501	57	0.8	739.0635	9	70.89	1.72E+06	5	12952	17041103.	14	27	83	Oxidation	M38:Oxidation (M):1000.00
3	115	P02654|APOC1_HUMAN	P.DVSSALDKLKEFGNTLEDKARELISRIKQSELSAKM(+15.99)REWFSETFQKVKEKLKIDS	Y	28.9	6444.4	55	2.4	645.4488	10	70.72		6	13448	17041104.	1	29	83	Oxidation	M36:Oxidation (M):1000.00
55	59	tr|C9JEU5|C9JEU5_HUMAN	K.AIQLTYNPDESSKPNMIDAATLK.S	Y	64.07	2519.258	23	0	840.7599	3	47.59	3.86E+05	5	8602	17041103.	1	89	111		
55	59	tr|C9JEU5|C9JEU5_HUMAN	G.HLSPTGTTEFWLGNEKIHLIS.T	Y	43.28	2379.222	21	-1.2	794.0804	3	52.41	2.02E+04	5	9498	17041103.	1	251	271		
55	59	tr|C9JEU5|C9JEU5_HUMAN	T.I(+42.01)GEGQQHHLGGAKQAGDV	Y	34.23	1842.897	18	-0.3	615.3062	3	29.41	3.93E+05	5	5205	17041103.	1	428	445	Acetylatioı	I1:Acetylation (N-term):1000.00
30	169	Q96PD5|PGRP2_HUMAN	R.EPPPRTLPATDLQ	Y	65.89	1433.751	13	1.4	717.884	2	36.91	7.82E+06	6	6551	17041104.	7	564	576		
30	169	Q96PD5|PGRP2_HUMAN	E.PPPRTLPATDLQ	Y	48.65	1304.709	12	0.1	653.3618	2	34.73	8.86E+05	6	6153	17041104.	3	565	576		

85	189	tr|A0A075B6J7|A0A075B6J7_HUMAN	T.SYVLTQPPSVS.V	Y	60.34	1176.603	11	0.3	589.3088	2	44.9	1.23E+06	5	8097	17041103.	1	20	30		
85	189	tr|A0A075B6J7|A0A075B6J7_HUMAN	S.YVLTQPPSVS.V	Y	58.27	1089.571	10	0.4	545.7928	2	44.35	2.72E+07	5	7997	17041103.	3	21	30		
85	189	tr|A0A075B6J7|A0A075B6J7_HUMAN	L.TQPPSVS.V	N	33.79	714.3548	7	-1.3	715.3611	1	53.42		5	9688	17041103.	1	24	30		
57	187	P10412|H14_HUMAN	M.S(+42.01)ETAPAAPAAPAPAEKTPVKKKA.R	Y	61.03	2272.243	23	0.8	569.0684	4	26.18	3.88E+05	6	4589	17041104.	2	2	24	Acetylatioı	S1:Acetylation (N-term):1000.00
57	187	P10412|H14_HUMAN	M.S(+42.01)ETAPAAPAAPAPAEKTPV.K	Y	52.53	1816.921	19	1.3	909.4688	2	41.15	1.23E+05	6	7358	17041104.	2	2	20	Acetylatioı	S1:Acetylation (N-term):1000.00
57	187	P10412|H14_HUMAN	M.S(+42.01)ETAPAAPAAPAPAEKTPVK.K	Y	40.25	1945.016	20	1.6	973.5166	2	33.9	5.00E+04	5	6045	17041103.	2	2	21	Acetylatioı	S1:Acetylation (N-term):1000.00
35	73	Q86SQ4|GP126_HUMAN	F.THFGVLMDLPRSAS.Q	Y	67.53	1529.766	14	0.3	765.8905	2	45.42	2.92E+05	6	8156	17041104.	4	841	854		
35	73	Q86SQ4|GP126_HUMAN	F.THFGVLMDLPRSASQLDA.R	Y	63.48	1956.973	18	2.4	653.3331	3	51.56	1.88E+06	6	9458	17041104.	2	841	858		
35	73	Q86SQ4|GP126_HUMAN	F.THFGVLM(+15.99)DLPRSAS.Q	Y	51.96	1545.761	14	0.9	516.2614	3	40.74	4.43E+05	6	7282	17041104.	1	841	854	Oxidation	M7:Oxidation (M):1000.00
35	73	Q86SQ4|GP126_HUMAN	F.THFGVLM(+15.99)DLPRSASQLDA.R	Y	42.88	1972.968	18	-1.5	658.6622	3	47.84	1.31E+05	5	8649	17041103.	1	841	858	Oxidation	M7:Oxidation (M):1000.00
84	144	P01717|LV403_HUMAN	SYELTQPPSVS.V	Y	62.43	1206.577	11	0.4	604.2959	2	42.92	8.51E+06	5	7735	17041103.	3	1	11		
84	144	P01717|LV403_HUMAN	S.YELTQPPSVS.V	Y	47.96	1119.545	10	0.5	560.78	2	42.28	4.80E+06	5	7617	17041103.	1	2	11		
84	144	P01717|LV403_HUMAN	L.TQPPSVS.V	N	33.79	714.3548	7	-1.3	715.3611	1	53.42		5	9688	17041103.	1	5	11		
84	145	tr|A0A075B6J4|A0A075B6J4_HUMAN	A.SYELTQPPSVS.V	Y	62.43	1206.577	11	0.4	604.2959	2	42.92	8.51E+06	5	7735	17041103.	3	20	30		
84	145	tr|A0A075B6J4|A0A075B6J4_HUMAN	S.YELTQPPSVS.V	Y	47.96	1119.545	10	0.5	560.78	2	42.28	4.80E+06	5	7617	17041103.	1	21	30		
84	145	tr|A0A075B6J4|A0A075B6J4_HUMAN	L.TQPPSVS.V	N	33.79	714.3548	7	-1.3	715.3611	1	53.42		5	9688	17041103.	1	24	30		
36	149	P01702|LV104_HUMAN	Q(-17.03)SVLTQPPSVS.A	Y	56.41	1124.571	11	0.7	563.2933	2	53.46	2.07E+07	5	9695	17041103.	4	1	11	Pyro-glu fr	Q1:Pyro-glu from Q:1000.00
36	149	P01702|LV104_HUMAN	Q.SVLTQPPSVS.A	Y	53.78	1013.539	10	1.2	507.7776	2	40.2	1.06E+06	5	7223	17041103.	2	2	11		
36	149	P01702|LV104_HUMAN	L.TQPPSVS.A	N	33.79	714.3548	7	-1.3	715.3611	1	53.42		5	9688	17041103.	1	5	11		
36	154	tr|A0A0B4J1U3|A0A0B4J1U3_HUMAN	A.Q(-17.03)SVLTQPPSVS.E	Y	56.41	1124.571	11	0.7	563.2933	2	53.46	2.07E+07	5	9695	17041103.	4	20	30	Pyro-glu fr	Q1:Pyro-glu from Q:1000.00
36	154	tr|A0A0B4J1U3|A0A0B4J1U3_HUMAN	Q.SVLTQPPSVS.E	Y	53.78	1013.539	10	1.2	507.7776	2	40.2	1.06E+06	5	7223	17041103.	2	21	30		
36	154	tr|A0A0B4J1U3|A0A0B4J1U3_HUMAN	L.TQPPSVS.E	N	33.79	714.3548	7	-1.3	715.3611	1	53.42		5	9688	17041103.	1	24	30		
46	275	O00187|MASP2_HUMAN	A.TPLGPKWPEPVFGRLA.S	Y	71.99	1763.972	16	0.4	588.9982	3	53.02	3.70E+06	5	9613	17041103.	2	16	31		
38	135	P18135|KV312_HUMAN	G.EIVLTQSPGTLS.L	Y	61.16	1243.666	12	-0.2	622.8401	2	47.57	2.58E+07	5	8598	17041103.	6	21	32		
38	135	P18135|KV312_HUMAN	E.IVLTQSPGTLS.L	Y	49.31	1114.623	11	0.4	558.3192	2	44.19	5.64E+05	5	7967	17041103.	1	22	32		
87	14051	P06310|KV206_HUMAN	G.DVVMTQSPLSLPVT.L	Y	60.1	1485.775	14	-0.1	743.8947	2	63.46	1.42E+06	5	11570	17041103.	1	21	34		
87	14051	P06310|KV206_HUMAN	G.DVVM(+15.99)TQSPLSLPVT.L	Y	54.28	1501.77	14	-1.4	751.8911	2	58.73	4.66E+05	5	10683	17041103.	1	21	34	Oxidation	M4:Oxidation (M):1000.00
87	14051	P06310|KV206_HUMAN	G.DVVMTQSPLS.L	Y	48.57	1075.522	10	0.4	538.7685	2	46.3	1.09E+06	5	8358	17041103.	2	21	30		
63	160	P01344|IGF2_HUMAN	K.FFQYDTWKQSTQ.R	Y	60.31	1577.715	12	0.8	789.8654	2	44.59	2.89E+05	6	8003	17041104.	2	113	124		
63	160	P01344|IGF2_HUMAN	K.FFQYDTWKQSTQRL.R	Y	56.02	1846.9	14	0.6	616.6411	3	45.75	6.63E+06	6	8222	17041104.	4	113	126		
152	14029	P02652|APOA2_HUMAN	L.SYFVELGTQPATQ	Y	59.79	1439.693	13	3.2	720.8562	2	51.58	1.40E+05	6	9460	17041104.	1	88	100		
152	14029	P02652|APOA2_HUMAN	A.GTELVNFLSYFVELGTQPATQ	Y	34.18	2313.153	21	1.9	1157.586	2	89.74		6	17871	17041104.	1	80	100		
152	14029	P02652|APOA2_HUMAN	V.TDYGKDLMEKVKSPEL.Q	Y	32.71	1851.929	16	0.2	618.317	3	43.83	7.91E+04	6	7865	17041104.	1	42	57		
58	143	tr|A0A075B6R9|A0A075B6R9_HUMAN	G.DIVMTQTPLSSPVT.L	Y	66.04	1487.754	14	-0.2	744.8842	2	56.52	1.49E+06	5	10271	17041103.	2	21	34		
58	143	tr|A0A075B6R9|A0A075B6R9_HUMAN	G.DIVM(+15.99)TQTPLSSPVT.L	Y	57.73	1503.749	14	0.7	752.8823	2	51.14	4.30E+05	5	9261	17041103.	2	21	34	Oxidation	M4:Oxidation (M):1000.00
58	143	tr|A0A075B6R9|A0A075B6R9_HUMAN	G.DIVMTQTPLS.S	N	43.25	1103.553	10	-0.5	552.7836	2	50.77	6.02E+05	5	9192	17041103.	1	21	30		
56	14009	P00734|THRB_HUMAN	T.SEYQTFFNPR.T	Y	49.02	1287.588	10	-0.2	644.8013	2	42.24	7.97E+05	6	7573	17041104.	2	318	327		
56	14009	P00734|THRB_HUMAN	S.EYQTFFNPR.T	Y	48.18	1200.556	9	3.3	601.2875	2	41.45	5.51E+04	5	7454	17041103.	2	319	327		
56	14009	P00734|THRB_HUMAN	S.Q(-17.03)HVFLAPQQARSLLQRV.R	Y	42.05	1973.096	17	-0.7	658.7054	3	52.53	1.87E+05	5	9519	17041103.	2	25	41	Pyro-glu fr	Q1:Pyro-glu from Q:1000.00
83	14090	P00740|FA9_HUMAN	C.TVFLDHENANKILNRPK.R	Y	63.42	2008.085	17	0.5	670.3694	3	33.16	2.27E+05	6	5868	17041104.	3	29	45		
83	14090	P00740|FA9_HUMAN	V.FLDHENANKILNRPK.R	Y	46.18	1807.969	15	-0.6	452.9993	4	29.33	6.63E+05	6	5165	17041104.	2	31	45		
228	141	O14791|APOL1_HUMAN	A.EEAGARVQQNVPSGTDTGDPQSKPLG.D	Y	63.56	2637.263	26	0	880.0949	3	32.21	1.25E+06	6	5692	17041104.	1	28	53		
228	141	O14791|APOL1_HUMAN	A.EEAGARVQQNVPSGTDTGDPQSKP.L	Y	43.94	2467.158	24	4.4	823.3967	3	28.33		6	4983	17041104.	1	28	51		
428	14063	Q9UGM3|DMBT1_HUMAN	R.DVGSYQEKVDVVLGPIQLQTPPRREEEPR	Y	85.33	3333.732	29	0.7	667.7541	5	48.35	2.31E+06	6	8798	17041104.	1	2385	2413		
91	74	P32926|DSG3_HUMAN	R.IETKGQYDEEEMTMQQAKRRQ.K	Y	56.98	2598.217	21	0.2	650.5615	4	28.23	3.66E+05	6	4966	17041104.	1	27	47		
91	74	P32926|DSG3_HUMAN	G.ELRIETKGQYDEEEMTMQQAKRRQ.K	Y	55.84	2996.444	24	-0.7	600.2957	5	32.59	8.29E+05	6	5762	17041104.	3	24	47		
97	14057	O94985|CSTN1_HUMAN	A.AQPQFVHPEHRSFVDLSGHNLANPHPF.A	Y	52.76	3077.501	27	2.3	616.5089	5	42.62	5.86E+05	6	7645	17041104.	1	827	853		
97	14057	O94985|CSTN1_HUMAN	H.MAAQPQFVHPEHRSFVDLSGHNLANPHPF.A	Y	46.59	3279.578	29	-0.6	656.9225	5	44.14	1.82E+05	6	7921	17041104.	1	825	853		
52	14004	tr|A0A075B6L0|A0A075B6L0_HUMAN	T.VAWKADSSPVKAGVE.T	Y	47.6	1542.804	15	-0.9	515.2749	3	33.31	9.50E+04	5	5934	17041103.	1	40	54		
52	14004	tr|A0A075B6L0|A0A075B6L0_HUMAN	T.VAWKADSSPVKAGVETTTPSKQSNNKYAASSYLS.L	Y	37.52	3571.779	34	-4.7	893.9479	4	37.82	2.34E+05	5	6773	17041103.	1	40	73		
52	14004	tr|A0A075B6L0|A0A075B6L0_HUMAN	K.AGVETTTPSKQSNNKYAASSYLS.L	Y	36.15	2403.155	23	1.9	802.0605	3	33.38	2.52E+06	5	5947	17041103.	2	51	73		
101	14034	P01031|CO5_HUMAN	G.Q(-17.03)EQTYVISAPKIFRVG.A	Y	58.49	1817.968	16	-2	909.9893	2	56.05	2.20E+05	5	10181	17041103.	1	19	34	Pyro-glu fr	Q1:Pyro-glu from Q:1000.00
139	4651	P01023|A2MG_HUMAN	D.LSFSPSQSLPASHAHLRVT.A	Y	49.02	2034.065	19	0	509.5234	4	39.19	7.72E+04	5	7030	17041103.	1	570	588		
139	4651	P01023|A2MG_HUMAN	A.SVSGKPQYMVLVPSLLH.T	Y	45.97	1854.007	17	1.6	619.0107	3	53.31	3.01E+05	5	9666	17041103.	1	24	40		
187	87	P35579|MYH9_HUMAN	M.A(+42.01)QQAADKYLYVDKNF.I	Y	79.61	1814.884	15	-3.4	908.4462	2	50.41	1.76E+05	6	9240	17041104.	2	2	16	Acetylatioı	A1:Acetylation (N-term):1000.00
266	14101	P54710|ATNG_HUMAN	M.TGLSMDGGGSPKGDVDPFYYDYETVRNGGL.I	Y	62.25	3166.419	30	5.6	1056.486	3	56.89	1.15E+05	6	10435	17041104.	1	2	31		
266	14101	P54710|ATNG_HUMAN	M.TGLSMDGGGSPKGDVDP.F	Y	33.44	1588.704	17	-6.7	795.3539	2	35.21	6.87E+04	6	6240	17041104.	1	2	18		
62	14046	tr|A0A075B6P5|A0A075B6P5_HUMAN	G.DIVMTQSPLS.L	Y	52.51	1089.538	10	0.8	545.7765	2	49.96	2.00E+06	5	9042	17041103.	2	21	30		
62	14046	tr|A0A075B6P5|A0A075B6P5_HUMAN	G.DIVMTQSPLSLPVT.P	Y	38.11	1499.791	14	-5.4	750.8985	2	59.94	1.87E+04	5	10913	17041103.	1	21	34		
62	14046	tr|A0A075B6P5|A0A075B6P5_HUMAN	G.DIVM(+15.99)TQSPLS.L	Y	37.6	1105.533	10	-0.1	553.7734	2	43.25	1.66E+06	5	7795	17041103.	1	21	30	Oxidation	M4:Oxidation (M):1000.00
264	63	Q8WWZ8|OIT3_HUMAN	R.GAGGEDSAGLQGQTLTGGPIRIDWED	Y	76.33	2599.215	26	-1.8	867.4107	3	57.09	1.37E+06	6	10471	17041104.	2	520	545		
141	14062	P10124|SRGN_HUMAN	F.HDNLRSLDRNLPSDSQDLGQHGLEEDFML	Y	59.9	3350.559	29	1.3	671.1199	5	51.64	3.90E+05	6	9471	17041104.	2	130	158		
265	55	P00748|FA12_HUMAN	N.KPGVYTDVAYYLAWIREHTVS	Y	75	2467.253	21	0.3	823.4253	3	63.16	4.18E+05	5	11511	17041103.	2	595	615		
103	161	P00488|F13A_HUMAN	M.S(+42.01)ETSRTAFGGRRAVPPNNSNAAEDDLPTVELQGVVPR.G	Y	74.48	3948.968	37	1.2	988.2504	4	48.2	1.16E+07	5	8716	17041103.	3	2	38	Acetylatioı	S1:Acetylation (N-term):1000.00
160	98	P01308|INS_HUMAN	R.EAEDLQVGQVELGGGPGAGSLQPLALEGSLQ.K	Y	73.97	3018.514	31	0.1	1007.179	3	68.5	4.36E+06	6	12918	17041104.	3	57	87		
104	113	P04196|HRG_HUMAN	K.SGFPQVSMFFTHTFPK	Y	73.96	1856.892	16	0.5	619.9716	3	58.16	3.53E+06	5	10578	17041103.	3	510	525		
104	113	P04196|HRG_HUMAN	K.SGFPQVSM(+15.99)FFTHTFPK	Y	55.31	1872.887	16	0.2	625.303	3	55.22	5.20E+05	5	10026	17041103.	1	510	525	Oxidation	M8:Oxidation (M):1000.00
61	14039	P04433|KV309_HUMAN	G.EIVLTQSPATLS.L	Y	59.1	1257.682	12	0.4	629.8483	2	49.34	7.53E+06	5	8924	17041103.	3	21	32		
61	445	tr|A0A0A0MRZ8|A0A0A0MRZ8_HUMAN	G.EIVLTQSPATLS.L	Y	59.1	1257.682	12	0.4	629.8483	2	49.34	7.53E+06	5	8924	17041103.	3	21	32		
82	14050	P04431|KV123_HUMAN	C.DIQMTQSPSSLS.A	Y	58.93	1292.592	12	0.4	647.3034	2	42.87	1.55E+06	5	7725	17041103.	2	23	34		
82	14050	P04431|KV123_HUMAN	C.DIQM(+15.99)TQSPSSLS.A	Y	39.88	1308.587	12	-0.6	655.3003	2	34.33	2.22E+05	5	6126	17041103.	2	23	34	Oxidation	M4:Oxidation (M):1000.00
70	52	P02452|CO1A1_HUMAN	D.FSFLPQPPQ.E	Y	47.1	1059.539	9	1.9	530.7778	2	55.98	1.69E+05	6	10269	17041104.	1	1198	1206		
379	167	Q86UX2|ITIH5_HUMAN	S.Q(-17.03)EEAQSWGHSSEQDGLRVPR.Q	Y	65.93	2278.036	20	-0.5	760.3523	3	38.62	3.00E+06	6	6872	17041104.	1	18	37	Pyro-glu fr	Q1:Pyro-glu from Q:1000.00
107	14138	Q14767|LTBP2_HUMAN	A.Q(-17.03)RDPVGRYEPAGGDANRLRRPGGSYPAAAAAKV.Y	Y	49.53	3405.74	33	1.4	568.6315	6	35.69	2.98E+05	5	6379	17041103.	2	36	68	Pyro-glu fr	Q1:Pyro-glu from Q:1000.00
107	14138	Q14767|LTBP2_HUMAN	A.Q(-17.03)RDPVGRYEPAGGDANRLRRPGG.S	Y	30.67	2476.243	23	0.9	496.2564	5	31.23	1.10E+05	6	5512	17041104.	1	36	58	Pyro-glu fr	Q1:Pyro-glu from Q:1000.00
99	716	P08514|ITA2B_HUMAN	R.Q(-17.03)IFLPEPEQPSR.L	Y	51.21	1422.714	12	-0.1	712.3643	2	58.14	1.15E+06	6	10668	17041104.	2	891	902	Pyro-glu fr	Q1:Pyro-glu from Q:1000.00
430	14093	O00264|PGRC1_HUMAN	K.IVRGDQPAASGDSDDDEPPPLPRL.K	Y	64.17	2516.214	24	2.3	839.7473	3	43.36	2.72E+05	6	7780	17041104.	1	45	68		
37	14164	P06319|LV605_HUMAN	A.NFMLTQPHSVS.E	Y	45.89	1259.597	11	0.6	630.806	2	38.97	5.82E+06	5	6988	17041103.	2	20	30		
37	14164	P06319|LV605_HUMAN	A.NFM(+15.99)LTQPHSVS.E	Y	45.14	1275.592	11	-0.1	638.8031	2	35.58	2.50E+06	5	6358	17041103.	3	20	30	Oxidation	M3:Oxidation (M):1000.00
37	14164	P06319|LV605_HUMAN	N.FMLTQPHSVS.E	Y	31.48	1145.554	10	0.4	573.7845	2	36.9	4.22E+05	5	6602	17041103.	1	21	30		
262	14052	P04207|KV308_HUMAN	G.EIVM(+15.99)TQSPATLS.V	Y	48.92	1291.633	12	1.7	646.8248	2	39.39	9.25E+05	5	7067	17041103.	1	21	32	Oxidation	M4:Oxidation (M):1000.00
267	14107	Q9NZP8|C1RL_HUMAN	G.SVLLAQELPQQLT.S	Y	59.53	1438.803	13	0.9	720.4095	2	58.86	2.29E+06	5	10709	17041103.	2	41	53		
109	14177	P01716|LV402_HUMAN	YDLTQPPSVS.V	Y	39.9	1105.529	10	1.3	553.7726	2	43.82	1.66E+06	5	7900	17041103.	2	1	10		
109	14177	P01716|LV402_HUMAN	L.TQPPSVS.V	N	33.79	714.3548	7	-1.3	715.3611	1	53.42		5	9688	17041103.	1	4	10		
380	245	P16403|H12_HUMAN	M.S(+42.01)ETAPAAPAAAPPAEKAPVKKKAA.K	Y	55.23	2313.269	24	1.1	579.3252	4	25.98	2.18E+05	6	4552	17041104.	1	2	25	Acetylatioı	S1:Acetylation (N-term):1000.00
516	14115	tr|F5H3P3|F5H3P3_HUMAN	M.T(+42.01)EKAPEPHVEEDDDDELDSKLNY.K	Y	55.14	2729.183	23	-0.8	910.7341	3	42.83	1.40E+05	6	7683	17041104.	1	2	24	Acetylatioı	T1:Acetylation (N-term):1000.00

427	3391	tr|A0A0C4DH07|A0A0C4DH07_HUMAN	R.EAPYGAPRFDMPDFEDDGGPYGESEAPAPPGPGTRWPYRSRDT.R	Y	54.49	4710.078	43	2.7	943.0255	5	52.32	4.86E+05	6	9598	17041104.	1	1426	1468		
227	62	P39060|COIA1_HUMAN	A.DDILASPPRLPEPQPYPGAPHHSS.Y	Y	41.81	2577.261	24	-0.4	645.3223	4	44.56	2.77E+05	6	7997	17041104.	1	1534	1557		
164	14184	tr|A0A075B6J9|A0A075B6J9_HUMAN	A.Q(-17.03)SALTQPPSVS.G	Y	37.26	1096.54	11	0.4	549.2775	2	48.63	1.24E+06	5	8793	17041103.	2	20	30	Pyro-glu fr	Q1:Pyro-glu from Q:1000.00
164	14184	tr|A0A075B6J9|A0A075B6J9_HUMAN	L.TQPPSVS.G	N	33.79	714.3548	7	-1.3	715.3611	1	53.42		5	9688	17041103.	1	24	30		
518	14120	P25311|ZA2G_HUMAN	A.QPLVVPWEAS	Y	54.08	1124.587	10	1.2	563.3012	2	57.36	3.78E+05	5	10429	17041103.	1	289	298		
81	4729	Q9Y490|TLN1_HUMAN	Q.QQYNRVGKVEHGSVALPAIMRSGASGPENFQVG.S	Y	53.2	3482.748	33	-0.9	871.6934	4	44.22	2.45E+05	5	7973	17041103.	4	434	466		
81	4729	Q9Y490|TLN1_HUMAN	Q.Q(-17.03)QYNRVGKVEHGSVALPAIMRSGASGPENFQVG.S	Y	48.47	3465.721	33	1.7	867.4391	4	48.12	4.41E+05	5	8701	17041103.	1	434	466	Pyro-glu fr	Q1:Pyro-glu from Q:1000.00
517	14116	P01765|HV304_HUMAN	EVQLLESGGGLVQPGGSLR.L	Y	53.19	1895.011	19	3.1	948.5159	2	50.66	5.08E+04	5	9170	17041103.	1	1	19		
165	14212	P01718|LV404_HUMAN	L.TQPPSVS.V	N	33.79	714.3548	7	-1.3	715.3611	1	53.42		5	9688	17041103.	1	4	10		
165	14212	P01718|LV404_HUMAN	Y(+42.01)ALTQPPSVS.V	Y	33.16	1103.55	10	2.7	552.7837	2	47.44	2.69E+05	5	8572	17041103.	2	1	10	Acetylatio	Y1:Acetylation (N-term):1000.00
77	14013	P05060|SCG1_HUMAN	P.ADASEAHESSSRGEAGAPGEEDIQGPTKADTEKWAEGGGHSRE.R	Y	34.47	4392.935	43	0.2	733.1633	6	31.69	2.26E+05	6	5597	17041104.	1	90	132		
161	14144	P01719|LV501_HUMAN	YVLSQPPSVS.V	Y	49.24	1075.555	10	0.3	538.7849	2	42.29	4.86E+05	5	7619	17041103.	3	1	10		
519	14147	P59666|DEF3_HUMAN	A.EPLQARADEVAAAPEQI.A	Y	48.34	1806.911	17	1.3	904.4641	2	43.45	1.62E+05	6	7795	17041104.	1	20	36		
268	14152	P05181|CP2E1_HUMAN	N.Q(-17.03)EFPDPEKF.K	Y	47.3	1118.492	9	0.9	560.2537	2	54.82	4.92E+05	6	10058	17041104.	2	401	409	Pyro-glu fr	Q1:Pyro-glu from Q:1000.00
520	69	P09681|GIP_HUMAN	G.EKKEGHFSALPSLPVGSHAKVSSPQPRGPR.Y	Y	46.72	3179.695	30	0.5	455.2497	7	31.33	2.53E+05	6	5530	17041104.	1	22	51		
521	14151	P01595|KV103_HUMAN	DIQMTQSPSPLS.A	Y	46.64	1302.613	12	-0.1	652.3135	2	46.72	7.77E+04	5	8435	17041103.	1	1	12		
429	14154	tr|A0A0B4J1V0|A0A0B4J1V0_HUMAN	C.EVQLVESGGGLVKPGGSLR.L	Y	46.56	1881.032	19	0.1	628.0179	3	41.03	1.42E+05	5	7376	17041103.	1	20	38		
108	14156	P01624|KV306_HUMAN	EIVMTQSPVT.L	Y	46.36	1103.553	10	0.2	552.784	2	42.52		5	7660	17041103.	2	1	10		
108	14156	P01624|KV306_HUMAN	EIVM(+15.99)TQSPVT.L	Y	42.07	1119.548	10	1.3	560.7821	2	35.28	3.14E+05	5	6303	17041103.	2	1	10	Oxidation	M4:Oxidation (M):1000.00
522	14157	P01708|LV205_HUMAN	Q(-17.03)SALTQPRSVS.G	Y	46.08	1155.588	11	-0.1	578.8014	2	34.08	9.02E+05	5	6078	17041103.	1	1	11	Pyro-glu fr	Q1:Pyro-glu from Q:1000.00
102	14056	P21675|TAF1_HUMAN	C.KLM(+15.99)PPPPPP.P	Y	35.53	988.5415	9	-0.5	495.2778	2	33.98	2.15E+05	6	6016	17041104.	1	154	162	Oxidation	M3:Oxidation (M):1000.00
123	256	Q02388|CO7A1_HUMAN	R.DGPPGLPGTPGPPGPPGP.K	Y	28.66	1562.773	18	2.4	782.3956	2	39.14		6	6968	17041104.	1	2075	2092		
88	195	P01042|KNG1_HUMAN	K.RPPGFSPF.R	Y	43.05	903.4602	8	-0.1	452.7373	2	41.14	4.73E+06	6	7356	17041104.	5	381	388		
523	221	Q9Y287|ITM2B_HUMAN	H.FENKFAVET.L	Y	41.91	1083.524	9	1	542.7697	2	35.53	1.97E+05	6	6298	17041104.	1	254	262		
162	14176	P36955|PEDF_HUMAN	H.LTFPLDYHLNQPFIFVLRDTDTGALLFIGKILDPRGP	Y	41.45	4212.277	37	-0.1	843.4626	5	80.83	6.31E+07	6	15883	17041104.	2	382	418		
136	2569	Q00975|CAC1B_HUMAN	A.RHKAQPAHEAVEKETTEKEATEKEAEIVEADKE.K	Y	27.33	3759.855	33	-0.9	752.9776	5	37.06	6.60E+05	6	6578	17041104.	1	982	1014		
163	14182	P01603|KV111_HUMAN	DIQM(+15.99)TQSPSTLS.V	Y	39.37	1322.602	12	1.4	662.3094	2	34.22	8.35E+04	5	6105	17041103.	1	1	12	Oxidation	M4:Oxidation (M):1000.00
163	14182	P01603|KV111_HUMAN	DIQMTQSPSTLS.V	Y	38.44	1306.607	12	0.4	654.3112	2	43.42	5.13E+05	5	7826	17041103.	2	1	12		
269	4100	tr|A0A075B6J3|A0A075B6J3_HUMAN	S.YELTQPSSVS.V	Y	36.17	1109.524	10	1	555.7698	2	37.38	4.16E+05	5	6690	17041103.	2	21	30		
234	14272	Q9UJF2|NGAP_HUMAN	L.QPLSFQ.N	Y	27.54	718.365	6	-4	719.3694	1	51.03	1.12E+06	5	9240	17041103.	1	777	782		
381	14183	Q04695|K1C17_HUMAN	R.TIVEEVQDGKVISSREQVHQTT.R	Y	34.07	2482.266	22	-0.7	621.5734	4	35.96	3.65E+05	6	6375	17041104.	1	410	431		
431	14228	P07437|TBB5_HUMAN	F.SVVPSPKVSDTVVEPYN.A	Y	33.29	1815.925	17	0.7	908.9706	2	43.04	2.77E+05	6	7723	17041104.	1	168	184		
382	14216	O00391|QSOX1_HUMAN	R.AAPGQEPPEHMAELQRNEQEQPLGQWHLS.K	Y	32.39	3306.548	29	-0.1	827.6441	4	45.07	2.68E+06	5	8126	17041103.	1	615	643		
525	14236	P06314|KV404_HUMAN	G.DIVMTQSPDSLAV.S	Y	31.26	1374.67	13	-4.1	688.3395	2	56.36	1.55E+05	6	10338	17041104.	1	21	33		
524	14221	tr|U3KPZ7|U3KPZ7_HUMAN	G.PGPGPGHS.M	Y	31.09	704.3242	8	7.5	705.3367	1	33.89		5	6043	17041103.	1	353	360		
432	1312	P00736|C1R_HUMAN	G.SIPIPQKLFGEVT.S	Y	29.74	1427.802	13	0.8	714.909	2	55.16	2.10E+05	5	10016	17041103.	1	18	30		
528	14252	P14652|HXB2_HUMAN	S.QSATSPSP.A	Y	28.72	773.3555	8	3.2	774.3653	1	33.72	3.07E+04	5	6013	17041103.	1	109	116		
436	14270	P56693|SOX10_HUMAN	P.TVSPPG.V	Y	27.94	556.2856	6	1.3	557.2936	1	54.19		5	9833	17041103.	1	344	349		
271	14262	O43255|SIAH2_HUMAN	C.SKQPPPQPQHTPSP.A	Y	27.47	1524.768	14	-5.8	763.3871	2	36.31	7.94E+04	5	6492	17041103.	1	16	29		
