# Supplementary material for: Peptidomic analysis of endogenous plasma peptides from patients with pancreatic neuroendocrine tumours
Source: Rapid Commun Mass Spectrom. 2018 Jul 17;32(16):1414–24. doi: 10.1002/rcm.8183 (PMC6099210; doi:10.1002/rcm.8183)
Supplement: Supplementary file 1 — Data S1. Supporting information [file RCM-32-1414-s001.zip › Supplementary table 2.pdf]

Protein Gr	Protein ID	Protein Ac	Peptide	Unique	-10lgP	Mass	Length	ppm	m/z	z	RT	Area	Fraction	Scan	Source File	#Spec	Start	End	PTM	AScore
2	82	P10645|CI	K.HSGFEDELSEVLENQSSQAELKEAVEEPSSKDV.M	Y	71.72	3645.68	33	2.7	912.4299	4	13.37	9.71E+04	5	2725	17100403.	2	97	129		
2	82	P10645|CI	K.HSGFEDELSEVLENQSSQAELKEAVEEPSSKDVME.K	Y	62.11	3905.764	35	2	977.4502	4	13.74	1.01E+06	5	2800	17100403.	4	97	131		
2	82	P10645|CI	K.HSGFEDELSEVLENQSSQAELKEAVEEPSSKDVM.E	Y	57.33	3776.721	34	3.1	945.1904	4	13.95	2.33E+05	5	2850	17100403.	4	97	130		
2	82	P10645|CI	K.EEEGSANRRPEDQELESLSAIEAELEKVAHQLQALRR(-.98).G	Y	56.01	4229.143	37	3.2	705.8666	6	18.53	1.42E+05	5	3773	17100403.	4	420	456	Amidation	R37:Amidation:1000.00
2	82	P10645|CI	K.HSGFEDELSEVLENQSSQAELKEAVEEPSSKDVM(+15.99)E.K	Y	53.11	3921.759	35	0.6	981.4475	4	13.04	6.30E+04	5	2656	17100403.	2	97	131	Oxidation (M)	M34:Oxidation (M):1000.00
2	82	P10645|CI	R.GYPEEKKEEEGSANRRPEDQELESLSAIEAELEKVAHQLQALRR(-.98).G	Y	50.61	5060.555	44	3.7	844.4362	6	17.34	3.77E+05	5	3542	17100403.	4	413	456	Amidation	R44:Amidation:1000.00
2	82	P10645|CI	K.EEEGSANRRPEDQELESLSAIEAELEKVAHQLQALRRG	Y	36.59	4287.148	38	3.5	715.5344	6	18.68	4.48E+04	5	3799	17100403.	1	420	457		
2	82	P10645|CI	R.LEGQEEEEDNRDSSMKLSFRARAYGFRGPGPQL.R	Y	29.01	3768.791	33	1.3	629.14	6	9.07	7.24E+04	5	1788	17100403.	2	358	390		
2	82	P10645|CI	R.LEGQEEEEDNRDSSMKLSFRA.R	Y	28.19	2469.108	21	2.8	618.2859	4	6.39	2.10E+04	4	1170	17100402.	1	358	378		
2	82	P10645|CI	K.HSGFEDELSEVLENQSSQAELKE.A	Y	27.86	2604.183	23	0.9	869.0689	3	12.56	1.12E+04	4	2548	17100402.	2	97	119		
2	82	P10645|CI	K.HSGFEDELSEVLENQSSQAE.L	Y	23.68	2233.961	20	2.5	745.6628	3	12.12	8.57E+03	5	2465	17100403.	1	97	116		
2	82	P10645|CI	K.HSGFEDELSEVLENQSSQAEL.K	Y	22.59	2347.045	21	1.5	783.3568	3	14.18	1.26E+04	4	2894	17100402.	1	97	117		
1	137	P02675|FI	S.Q(-17.03)GVNDNEEGFFSARGHRPLDKKREEAPSLRPAPPPISGGGYR.A	Y	63.51	4571.28	42	3	762.8896	6	7.84	4.74E+05	5	1499	17100403.	2	31	72	Pyro-glu from Q	Q1:Pyro-glu from Q:1000.00
1	137	P02675|FI	R.EEAPSLRPAPPPISGGGY.R	Y	51.39	1793.895	18	1.5	897.956	2	9.3	4.57E+04	5	1839	17100403.	2	54	71		
1	137	P02675|FI	S.Q(-17.03)GVNDNEEGFFSARGHRPLDKKREEAPSLRPAPPPISGGGY.R	Y	51.01	4415.179	41	2.3	884.0451	5	8.74	1.48E+06	5	1705	17100403.	4	31	71	Pyro-glu from Q	Q1:Pyro-glu from Q:1000.00
1	137	P02675|FI	S.Q(-17.03)GVNDNEEGFFSARGHRPLD.K	Y	44.25	2227.004	20	1.1	743.3428	3	8.27	8.33E+05	5	1597	17100403.	3	31	50	Pyro-glu from Q	Q1:Pyro-glu from Q:1000.00
1	137	P02675|FI	S.Q(-17.03)GVNDNEEGFFSAR.G	Y	44.18	1551.659	14	0.8	776.8374	2	9.06	1.07E+04	4	1776	17100402.	2	31	44	Pyro-glu from Q	Q1:Pyro-glu from Q:1000.00
1	137	P02675|FI	S.Q(-17.03)GVNDNEEGFFSARGHRP.L	Y	39.28	1998.893	18	1.2	667.3058	3	6.59	1.11E+05	5	1227	17100403.	2	31	48	Pyro-glu from Q	Q1:Pyro-glu from Q:1000.00
1	137	P02675|FI	S.Q(-17.03)GVNDNEEGFFSARGHRPLD.K	Y	38.31	2355.099	21	2.5	589.7835	4	6.69	2.05E+05	5	1248	17100403.	4	31	51	Pyro-glu from Q	Q1:Pyro-glu from Q:1000.00
1	137	P02675|FI	G.VNDNEEGFFSARGHRPLD.K	Y	36.35	2058.951	18	2.8	515.7464	4	6.38	4.78E+04	4	1169	17100402.	2	33	50		
1	137	P02675|FI	S.Q(-17.03)GVNDNEEGFFSA.R	Y	33.18	1395.558	13	2.1	698.7877	2	10.9	3.29E+04	4	2195	17100402.	2	31	43	Pyro-glu from Q	Q1:Pyro-glu from Q:1000.00
1	137	P02675|FI	K.KREEAPSLRPAPPPISGGGYR.A	Y	32.98	2234.192	21	1.1	559.5558	4	5.62	1.69E+04	4	1010	17100402.	1	52	72		
1	137	P02675|FI	S.Q(-17.03)GVNDNEEGFFSARGHRPLDKKREEAPSLRPAPPPISGGGYRARP.A	Y	30.97	4895.472	45	1.2	816.9202	6	7.51	5.92E+04	4	1424	17100402.	2	31	75	Pyro-glu from Q	Q1:Pyro-glu from Q:1000.00
1	137	P02675|FI	E.EAPSLRPAPPPISGGGY.R	Y	30.44	1664.852	17	2	833.4351	2	9.11	8.12E+03	5	1797	17100403.	2	55	71		
1	137	P02675|FI	K.KREEAPSLRPAPPPISGGGY.R	Y	30.32	2078.091	20	0.4	693.7045	3	7.01	3.67E+03	5	1318	17100403.	1	52	71		
1	137	P02675|FI	G.VNDNEEGFFSARGHRPLDKKREEAPSLRPAPPPISGGGY.R	Y	26.74	4247.126	39	1.8	708.8629	6	7.75	9.02E+04	5	1480	17100403.	1	33	71		
1	137	P02675|FI	L.DKKREEAPSLRPAPPPISGGGY.R	Y	25.61	2321.213	22	3.1	581.3123	4	6.76	7.40E+03	5	1265	17100403.	1	50	71		
1	137	P02675|FI	V.NDNEEGFFSARGHRPLDKKREEAPSLRPAPPPISGGGY.R	Y	23.18	4148.058	38	-0.4	692.3499	6	7.69	2.08E+04	4	1463	17100402.	1	34	71		
12	133	P02766|T	N.DSGPRRYTIAALLSPYSYSTTAVVTNPKE	Y	72.25	3156.609	29	1.8	790.161	4	13.85	9.44E+05	5	2826	17100403.	3	119	147		
12	133	P02766|T	S.GPRRYTIAALLSPYSYSTTAVVTNPKE	Y	67.19	2954.55	27	2.5	739.6467	4	12.85	1.80E+05	5	2614	17100403.	3	121	147		
12	133	P02766|T	S.GPRRYTIAALLSPYSYSTTAVVTNPK.E	Y	60.68	2825.508	26	2.2	707.3857	4	12.79	5.83E+04	5	2603	17100403.	2	121	146		
12	133	P02766|T	D.SGPRRYTIAALLSPYSYSTTAVVTNPKE	Y	55.38	3041.582	28	3.6	761.4055	4	12.87	1.05E+05	5	2620	17100403.	2	120	147		
12	133	P02766|T	G.PRRYTIAALLSPYSYSTTAVVTNPKE	Y	42.88	2897.529	26	2.5	725.3912	4	12.82	1.67E+05	4	2605	17100402.	2	122	147		
4	68	P02671|FI	K.SSSYSKQFTSSTSYNRGDSTFESKSY.K	Y	52.08	2930.284	26	1.4	733.5793	4	5.22	8.38E+04	5	928	17100403.	2	576	601		
4	68	P02671|FI	G.SFRPDSPGSGNARPNNPDWGTFEEVSGNVSPGTR.R	Y	51.56	3587.641	34	1.1	897.9186	4	9.19	2.14E+04	5	1816	17100403.	2	392	425		
4	68	P02671|FI	A.DSGEGDFLAEGGGVR.G	Y	50.47	1464.648	15	1.6	733.3325	2	6.92	4.20E+04	5	1300	17100403.	1	21	35		
4	68	P02671|FI	T.ADSGEGDFLAEGGGVR.G	Y	49.81	1535.685	16	1.2	768.8508	2	6.64	8.44E+03	5	1239	17100403.	2	20	35		
4	68	P02671|FI	G.EGDFLAEGGGVR.G	Y	35.32	1205.568	12	1.3	603.7919	2	6.09	1.18E+04	5	1114	17100403.	2	24	35		
4	68	P02671|FI	M.DLGTLSGIGTLDGFRHRHPDEAAF.F	Y	32.76	2581.267	24	0.8	646.3246	4	12.02	2.55E+04	4	2437	17100402.	2	496	519		
4	68	P02671|FI	K.SSSYSKQFTSSTSYNRGDSTFESKSYKMA.D	Y	31.16	3260.457	29	0.3	653.0988	5	5.55	5.75E+04	5	998	17100403.	2	576	604		
4	68	P02671|FI	G.DFLAEGGGVR.G	Y	30.33	1019.504	10	2.4	510.7603	2	5.96	4.72E+03	5	1086	17100403.	2	26	35		
4	68	P02671|FI	K.DSHSLTTNIMEILRGDFSSANN.R	Y	29.99	2421.123	22	3.2	808.0508	3	17.42	2.43E+04	5	3560	17100403.	1	101	122		
4	68	P02671|FI	P.GSTGNRNPGSSGTGGTATWKPGSSGP.G	Y	29.19	2374.09	26	1.3	792.3716	3	3.24	1.36E+04	4	526	17100402.	2	303	328		
4	68	P02671|FI	K.SSSYSKQFTSSTSYNRGDSTFES.K	Y	26.94	2552.094	23	1.1	851.7062	3	5.15	4.73E+04	5	914	17100403.	2	576	598		
4	68	P02671|FI	K.MKPVPDLVPGNFK.S	Y	24.77	1440.78	13	2.3	721.3989	2	9.69	2.07E+04	5	1926	17100403.	1	226	238		
4	68	P02671|FI	K.ALTDMPQMR.M	Y	23.96	1061.5	9	2	531.7582	2	6.22	3.61E+03	4	1135	17100402.	1	250	258		
8	143	P62328|T	M.S(+42.01)DKPDMAEIEKFDKSKLKKTETQEKNPLPSKETIEQEKQAGES	Y	54.91	4960.486	43	2.8	827.7573	6	6.58	1.37E+06	5	1223	17100403.	4	2	44	Acetylation (N-ter	S1:Acetylation (N-term):1000.00
8	143	P62328|T	M.S(+42.01)DKPDMAEIEKFDKSKLK.K	Y	54.24	2150.093	18	2	538.5316	4	7.07	4.93E+04	5	1330	17100403.	2	2	19	Acetylation (N-ter	S1:Acetylation (N-term):1000.00
8	143	P62328|T	M.S(+42.01)DKPDMAEIEKFDKSKLKKTE.T	Y	53.26	2508.278	21	1.6	628.0778	4	6.03	3.47E+04	5	1100	17100403.	3	2	22	Acetylation (N-ter	S1:Acetylation (N-term):1000.00
8	143	P62328|T	M.S(+42.01)DKPDMAEIEKFDKSKLKKTET.Q	Y	43.37	2609.326	22	1	653.3394	4	6.1	2.81E+04	4	1108	17100402.	3	2	23	Acetylation (N-ter	S1:Acetylation (N-term):1000.00
8	143	P62328|T	M.S(+42.01)DKPDMAEIEKFDKSKL.K	Y	39.76	2021.998	17	2.1	506.5078	4	8.77	2.08E+05	4	1704	17100402.	3	2	18	Acetylation (N-ter	S1:Acetylation (N-term):1000.00
8	143	P62328|T	E.TQEKNPLPSKETIEQEKQAGES	Y	24.12	2470.219	22	0.1	618.562	4	3.25	5.18E+04	5	530	17100403.	1	23	44		
8	143	P62328|T	M.S(+42.01)DKPDMAEIEKFDKSKLKKTETQE.K	Y	23.02	2866.427	24	2.2	574.2939	5	6.07	5.46E+04	4	1102	17100402.	1	2	25	Acetylation (N-ter	S1:Acetylation (N-term):1000.00
8	143	P62328|T	M.S(+42.01)DKPDMAEIEKFDKSKLKKTETQ.E	Y	21.94	2737.384	23	1.7	548.485	5	6	1.53E+04	4	1088	17100402.	1	2	24	Acetylation (N-ter	S1:Acetylation (N-term):1000.00
7	80	P0C0L5|C(	Q.KPRLLLFSPSVVHLGVPLSVGVQLQDVPRG.Q	Y	58.07	3206.866	30	2.5	642.382	5	16.3	5.04E+04	5	3329	17100403.	4	20	49		
7	80	P0C0L5|C(	K.DDPDAPLQPVTPLQL.F	Y	41.25	1617.825	15	3.2	809.9223	2	15.06	2.55E+04	5	3078	17100403.	2	1429	1443		
7	80	P0C0L5|C(	R.GLEEELQFSLGSKINVKVGGNS.K	Y	40.42	2304.196	22	2.6	769.0746	3	13.19	6.91E+04	5	2690	17100403.	2	1353	1374		
7	80	P0C0L5|C(	Q.KPRLLLFSPSVVHLGVPLSVGVQLQDVPRGQVVKGSVFLRNPSRN.N	Y	37.76	4888.803	45	4.1	699.4106	7	15.07	1.74E+05	5	3079	17100403.	5	20	64		
7	80	P0C0L5|C(	K.DDPDAPLQPVTPLQLFEGRRN.R	Y	35.72	2377.203	21	2	793.4097	3	14.94	1.30E+06	4	3049	17100402.	4	1429	1449		
7	80	P0C0L5|C(	R.NGFKSHALQLNNRQI.R	Y	32.8	1738.923	15	1.6	580.649	3	5.2	1.11E+05	5	922	17100403.	2	1337	1351		
7	80	P0C0L5|C(	R.NGFKSHALQLNNRQIR.G	Y	30.95	1895.024	16	2.6	632.6835	3	3.4	1.56E+05	5	562	17100403.	2	1337	1352		
7	80	P0C0L5|C(	K.DDPDAPLQPVTPLQ.L	Y	24.27	1504.741	14	1.8	753.379	2	10.63	1.69E+04	5	2143	17100403.	1	1429	1442		
7	80	P0C0L5|C(	R.GLEEELQFSLGSK.I	Y	21.58	1435.72	13	1.2	718.8679	2	11.48	4.87E+03	5	2331	17100403.	1	1353	1365		
3	87	P01009|A	A.EDPQGDAAQKTDTSHHDQDHPTFNKITPNLAEFAFSLYRQLAHQ.S	Y	53.61	5018.361	44	3.2	837.4034	6	17.53	9.62E+05	5	3581	17100403.	7	25	68		
3	87	P01009|A	G.DAAQKTDTSHHDQDHPTFNKITPNLAEFAFSLYRQLAHQ.S	Y	44.77	4492.158	39	3.2	899.4418	5	17.66	6.97E+03	5	3610	17100403.	6	30	68		
3	87	P01009|A	A.EDPQGDAAQKTDTSHHDQDHPTFNKITPNLAEFAFSLYRQLAH.Q	Y	40.81	4890.302	43	3.7	816.0607	6	17.21	1.87E+05	5	3517	17100403.	2	25	67		
3	87	P01009|A	A.EDPQGDAAQKTDTSHHDQDHPTFNKITPNLAEFAFSLYRQLA.H	Y	31.52	4753.243	42	3.8	793.2175	6	17.83	9.42E+04	5	3639	17100403.	1	25	66		
3	87	P01009|A	G.DAAQKTDTSHHDQDHPTFNKITPNLAEFAFSLYRQLAHQS.N	Y	29.91	4579.19	40	1.9	764.2072	6	17.64	9.71E+04	4	3614	17100402.	1	30	69		
3	87	P01009|A	A.EDPQGDAAQKTDTSHHDQDHPTFNKITPNLAEFAFSLYRQLAHQS.N	Y	25.47	5105.393	45	2.6	851.9083	6	17.56	2.94E+05	5	3588	17100403.	1	25	69		
3	87	P01009|A	E.AAGAMFLEAIPMSIPPEVKFNKPFVFLMIEQNTKSPLFMGKVVNPTQK	Y	25.03	5334.809	48	-2.1	890.1401	6	20.36	2.59E+05	4	4116	17100402.	2	371	418		
3	87	P01009|A	A.MFLEAIPMSIPPEVKFNKPFVFLMIEQNTKSPLFMGKVVNPTQK	Y	22.77	5064.676	44	9.7	845.1281	6	19.87	6.55E+04	5	4017	17100403.	1	375	418		
3	87	P01009|A	A.GAMFLEAIPMSIPPEVKFNKPFVFLMIEQNTKSPLFMGKVVNPTQK	Y	21.82	5192.734	46	0.2	866.4631	6	20.21	8.74E+04	5	4081	17100403.	1	373	418		
6	70	Q15942|Z	S.LANTQPRGPPASSPAPAPKFSPVTPKFTPVAS.K	Y	45.07	3214.714	32	1.3	804.6868	4	10.14	5.41E+04	5	2031	17100403.	2	247	278		
6	70	Q15942|Z	M.A(+42.01)APRPSPAISVSVSAPAF.Y	Y	36.31	1765.936	18	1.4	883.9766	2	13.87	4.72E+04	4	2829	17100402.	2	2	19	Acetylation (N-ter	A1:Acetylation (N-term):1000.00
6	70	Q15942|Z	F.HVQPQPQPKPQVQLHVQ.S	Y	35.43	1987.075	17	1.2	663.3665	3	4.68	2.04E+04	5	819	17100403.	2	223	239		
6	70	Q15942|Z	F.HVQPQPQPKPQVQLHVQSQT.Q	Y	34.05	2303.213	20	0.9	768.7458	3	4.55	4.08E+04	4	788	17100402.	3	223	242		

6	70	Q15942|Z	K.VNPFRPGDSEPPPAPGAQRAQ.M	Y	29.07	2187.082	21	1.3	730.0356	3	5.72	3.46E+04	5	1034	17100403.	2	36	56		
6	70	Q15942|Z	K.VNPFRPGDSEPPPAPGAQRAQMGRVG.E	Y	28.96	2687.335	26	0.9	672.8416	4	6.63	1.51E+04	5	1237	17100403.	2	36	61		
6	70	Q15942|Z	V.RSPGAPGPLTLKEVEELE.Q	Y	28.52	1921.016	18	2.6	641.3475	3	11	1.16E+04	5	2223	17100403.	2	343	360		
6	70	Q15942|Z	F.SPVTPKFTPVAS.K	Y	27.43	1229.666	12	2	615.8412	2	7.35	1.65E+04	4	1388	17100402.	2	267	278		
6	70	Q15942|Z	N.TQPRGPPASSPAPAPKFSPVTPKFTPVAS.K	Y	27.04	2916.55	29	0.7	730.1452	4	9.83	1.42E+04	4	1951	17100402.	2	250	278		
6	70	Q15942|Z	M.A(+42.01)APRPSPAISVSVS.A	Y	23.19	1379.741	14	2.2	690.8792	2	9.1	5.38E+04	5	1794	17100403.	1	2	15	Acetylation (N-ter	A1:Acetylation (N-term):1000.00
6	70	Q15942|Z	F.YAPQKKFGPVVAPKPKVNPFRPGDSEPPPAPGAQRAQ.M	Y	22.21	3923.096	37	0.7	654.857	6	6.9	2.26E+04	5	1296	17100403.	2	20	56		
10	83	O95810|S	A.SALVEGEIAEEAAEKAT.S	Y	50.78	1716.842	17	1.5	859.4294	2	9.21	1.60E+04	5	1819	17100403.	2	344	360		
10	83	O95810|S	Y.ALTSEEAERSDGDPVQPAVLQVHQTS	Y	44.35	2763.331	26	1.1	922.1187	3	7.97	4.06E+04	5	1527	17100403.	2	400	425		
10	83	O95810|S	M.G(+42.01)EDAAQAEKFQHPGSDMR.Q	Y	36.91	2014.88	18	0.6	672.6345	3	4.56	1.43E+04	5	793	17100403.	1	2	19	Acetylation (N-ter	G1:Acetylation (N-term):1000.00
10	83	O95810|S	M.G(+42.01)EDAAQAEKFQHPG.S	Y	32.31	1525.68	14	2.2	763.8488	2	4.22	2.92E+04	4	722	17100402.	1	2	15	Acetylation (N-ter	G1:Acetylation (N-term):1000.00
10	83	O95810|S	R.YEGSYALTSEEAERSDGDPVQPAVLQVHQTS	Y	31.97	3362.554	31	1.5	841.647	4	9.68	3.60E+04	5	1925	17100403.	4	395	425		
10	83	O95810|S	G.SYALTSEEAERSDGDPVQPAVLQVHQTS	Y	26.92	3013.427	28	1.8	1005.485	3	9.16	9.99E+03	5	1807	17100403.	1	398	425		
10	83	O95810|S	M.G(+42.01)EDAAQAEKFQHPGSDM.R	Y	26.23	1858.779	17	-0.6	930.3963	2	5.88	5.14E+03	4	1064	17100402.	2	2	18	Acetylation (N-ter	G1:Acetylation (N-term):1000.00
14	114	P37802|T	M.GTNRGASQAGMTGYGMPRQIL	Y	67.64	2165.047	21	1.7	722.6908	3	9.02	6.22E+04	5	1776	17100403.	2	179	199		
14	114	P37802|T	T.NRGASQAGMTGYGMPRQIL	Y	30.56	2006.978	19	0.7	670.0003	3	9.04	1.56E+04	4	1772	17100402.	2	181	199		
14	114	P37802|T	M.TGYGMPRQIL	Y	29.82	1134.586	10	1.6	568.301	2	8.96	1.90E+04	4	1753	17100402.	2	190	199		
14	114	P37802|T	A.GMTGYGMPRQIL	Y	25.53	1322.648	12	2.1	662.3324	2	10.29	4.31E+03	4	2055	17100402.	1	188	199		
14	114	P37802|T	M.A(+42.01)NRGPAYGLS.R	Y	23.83	1046.515	10	1.7	524.2654	2	5.35	1.49E+04	5	956	17100403.	1	2	11	Acetylation (N-ter	A1:Acetylation (N-term):1000.00
14	114	P37802|T	Q.MGTNRGASQAGMTGYGMPRQIL	Y	22.81	2296.087	22	1.5	766.3709	3	9.42	4.74E+03	5	1864	17100403.	1	178	199		
9	89	P13521|S	R.TNEIVEEQYTPQSLATLESVFQELGKLTGPNNQ.K	Y	49.24	3676.811	33	3.4	1226.615	3	21.97	3.12E+05	5	4423	17100403.	4	182	214		
9	89	P13521|S	P.VGPPKNDDTPNRQYWDEDLLMKVLEYLNQEKAEKGREHIA.K	Y	46.9	4738.345	40	3	677.9157	7	15.63	5.21E+05	5	3189	17100403.	5	571	610		
9	89	P13521|S	R.FPVGPPKNDDTPNRQYWDEDLLMKVLEYLNQEKAEKGREHIA.K	Y	46.02	4982.466	42	2.6	831.4204	6	16.21	1.51E+05	5	3307	17100403.	2	569	610		
9	89	P13521|S	P.VGPPKNDDTPNRQYWDEDLLMKVLEYLNQEKAEKGREH.I	Y	35.94	4554.224	38	1.6	651.6117	7	15.69	4.83E+04	5	3202	17100403.	2	571	608		
11	72	P07437|T	Y.NEATGGKYVPRAILVDLEPGTMDSVR.S	Y	42.85	2787.422	26	3.8	697.8655	4	12	1.56E+04	5	2438	17100403.	2	52	77		
11	72	P07437|T	F.VFGQSGAGNNWAKGHYTEGAELVDSVLDVVR.K	Y	39.45	3274.601	31	3.4	819.6602	4	15.55	2.52E+04	5	3174	17100403.	2	91	121		
11	72	P07437|T	S.DEHGIDPTGTYHGDSDLQLDRISVYY.N	Y	36.91	2965.336	26	2.7	742.3434	4	12.02	1.08E+04	5	2444	17100403.	1	26	51		
11	72	P07437|T	F.SVVPSPKVSDTVVEPYN.A	Y	31.83	1815.925	17	0.9	908.9708	2	8.89	3.61E+04	5	1744	17100403.	2	168	184		
11	72	P07437|T	G.GKYVPRAILVDLEPGTMDSVR.S	Y	28.98	2315.231	21	2.5	772.7527	3	11.29	7.77E+03	4	2281	17100402.	2	57	77		
11	72	P07437|T	G.GGTGSGMGTLLISKIREEYPDRIMNTF.S	Y	24.86	2942.463	27	2.4	736.6248	4	14.96	3.79E+04	4	3054	17100402.	1	141	167		
13	117	P01024|C	G.SPMYSIITPNILRLESEETMVLEAHDAQ.G	Y	51.47	3186.558	28	1.4	797.6478	4	17.57	4.95E+04	5	3591	17100403.	4	23	50		
13	117	P01024|C	G.SPMYSIITPNILRLES.E	Y	34.52	1832.971	16	1.9	917.4943	2	16.32	1.74E+04	4	3336	17100402.	2	23	38		
13	117	P01024|C	G.SPMYSIITPNILRLESEETMVLEAHDA.Q	Y	34.06	3058.499	27	5.2	1020.512	3	17.92	3.42E+04	5	3657	17100403.	2	23	49		
13	117	P01024|C	G.SPMYSIITPNILRLESEETMVLEAHDAQGDVPVTVTVHDFPG.K	Y	28.15	4607.256	42	0.7	1152.822	4	18.63	3.56E+04	4	3797	17100402.	2	23	64		
13	117	P01024|C	G.SPMYSIITPNILRLESEETMVLEAHDAQGDVPVTVTVHDFPGK.K	Y	23.23	4735.351	43	-0.5	948.077	5	17.5	2.27E+04	5	3575	17100403.	1	23	65		
5	138	P02654|A	G.TPDVSSALDKLKEFGNTLEDKARELISRIKQSELSAKMREWFSETFQKVKEKLKIDS	Y	60.74	6626.506	57	-1.6	829.3192	8	23.01	6.99E+07	5	4628	17100403.	14	27	83		
5	138	P02654|A	P.DVSSALDKLKEFGNTLEDKARELISRIKQSELSAKMREWFSETFQKVKEKLKIDS	Y	50.75	6428.405	55	-0.1	919.3508	7	22.66	3.29E+07	5	4555	17100403.	10	29	83		
15	91	P68366|T	G.AGKHVPRAVFVDLEPTVIDEIR.N	Y	51.24	2460.349	22	2.7	616.0961	4	12.04	4.32E+04	5	2447	17100403.	3	58	79		
15	91	P68366|T	A.SLRFDGALNVDLTEFQTNLVPYPRIHFPLA.T	N	42.76	3442.804	30	6.1	861.7134	4	19.53	2.75E+04	5	3956	17100403.	2	241	270		
15	91	P68366|T	A.SLRFDGALNVDLTEFQTN.L	N	33.77	2038.996	18	1.4	1020.507	2	14.31	1.76E+04	4	2920	17100402.	3	241	258		
25	136	P19823|IT	G.FEIPINGLSEFVDYEDLVELAPGKFQLVAENRRYQ.R	Y	68.04	4098.074	35	3	1025.529	4	20.42	1.65E+06	5	4119	17100403.	2	19	53		
25	136	P19823|IT	F.EIPINGLSEFVDYEDLVELAPGKFQLVAENRRYQ.R	Y	29.64	3951.005	34	3.4	988.762	4	19.01	1.46E+04	5	3863	17100403.	2	20	53		
20	142	P35579|M	M.A(+42.01)QQAADKYLYVDKNF.I	Y	56.23	1814.884	15	2.9	908.4518	2	10.61	3.66E+04	5	2138	17100403.	2	2	16	Acetylation (N-ter	A1:Acetylation (N-term):1000.00
20	142	P35579|M	M.A(+42.01)QQAADKYLY.V	Y	32.04	1211.582	10	2.1	606.7997	2	8.25	9.48E+03	5	1592	17100403.	2	2	11	Acetylation (N-ter	A1:Acetylation (N-term):1000.00
20	142	P35579|M	M.A(+42.01)QQAADKYLYVD.K	Y	28.08	1425.678	12	1.4	713.8471	2	9.52	4.99E+03	5	1887	17100403.	2	2	13	Acetylation (N-ter	A1:Acetylation (N-term):1000.00
16	92	Q9BQE3|T	G.AGKHVPRAVFVDLEPTVIDEVR.T	Y	45.73	2446.333	22	1.8	612.5917	4	11.13	3.39E+04	5	2250	17100403.	2	58	79		
16	92	Q9BQE3|T	A.SLRFDGALNVDLTEFQTNLVPYPRIHFPLA.T	N	42.76	3442.804	30	6.1	861.7134	4	19.53	2.75E+04	5	3956	17100403.	2	241	270		
16	92	Q9BQE3|T	A.SLRFDGALNVDLTEFQTN.L	N	33.77	2038.996	18	1.4	1020.507	2	14.31	1.76E+04	4	2920	17100402.	3	241	258		
22	139	P01019|A	N.KPEVLEVTLNRPFLFAVYDQSATALHFLGRVANPLSTA	Y	56.76	4184.242	38	2.6	1047.071	4	20.83	9.54E+05	5	4204	17100403.	4	448	485		
17	116	O15240|V	P.GRPEAQPPPLSSEHKEPVAGDAVPGPKDGSAPEVRGA.R	Y	37.53	3685.845	37	2.5	615.3163	6	5.79	4.86E+04	4	1044	17100402.	4	26	62		
17	116	O15240|V	A.APPGRPEAQPPPLSSEHKEPVAGDAVPGPKDGSAPEVRGA.R	Y	27.18	3950.987	40	1.2	659.506	6	6.12	1.78E+04	5	1121	17100403.	2	23	62		
17	116	O15240|V	R.Q(-17.03)QETAAAETETRTHTLTRVNLESPGPERVW.R	Y	24.37	3389.66	30	-1.5	848.421	4	9.77	9.19E+03	4	1937	17100402.	1	177	206	Pyro-glu from Q	Q1:Pyro-glu from Q:1000.00
19	110	P02765|F	D.PDAPPSPPLGAPGLPPAGSPPDSHVLLAAPPGHQLHRAHYDLRHTFMGVVSLGSPSGEVSHPRKT.R	Y	35.83	6663.41	65	5.6	833.9382	8	12.29	4.73E+05	4	2495	17100402.	1	275	339		
19	110	P02765|F	R.TVVQPSVGAAAGPVVPPCPGRIRHFKV	Y	31.57	2738.517	27	1.9	685.6378	4	9.73	4.36E+04	5	1935	17100403.	2	341	367		
19	110	P02765|F	D.APPSPPLGAPGLPPAGSPPDSHVLLAAPPGHQLHRAHYDLRHTFMGVVSLGSPSGEVSHPRKT.R	Y	31.51	6451.33	63	-5.9	922.6204	7	12.18	1.11E+05	4	2472	17100402.	2	277	339		
18	96	Q9Y490|T	Q.Q(-17.03)QYNRVGKVEHGSVALPAIMRSGASGPENFQVG.S	Y	35.1	3465.721	33	1.2	867.4386	4	11.18	9.99E+04	4	2257	17100402.	2	434	466	Pyro-glu from Q	Q1:Pyro-glu from Q:1000.00
18	96	Q9Y490|T	Q.L(+42.01)DEGPM(+15.99)GEPEGSF.V	Y	25.26	1421.566	13	9.3	711.7967	2	14.05	9.75E+03	5	2870	17100403.	1	1838	1850	Acetylation (N-ter	L1:Acetylation (N-term):1000.00;M6:Oxidation (M):1000.00
18	96	Q9Y490|T	Q.Q(-17.03)QYNRVGKVEHGSVALPAIM.R	Y	22.4	2179.121	20	1.3	727.3818	3	10.71	2.24E+04	5	2161	17100403.	1	434	453	Pyro-glu from Q	Q1:Pyro-glu from Q:1000.00
21	112	Q9NY65|T	A.SLRFDGALNVDLTEFQTN.L	N	33.77	2038.996	18	1.4	1020.507	2	14.31	1.76E+04	4	2920	17100402.	3	241	258		
21	112	Q9NY65|T	G.NGKHVPRAVMIDLEPTVVDEVR.A	Y	32.5	2473.311	22	2.3	619.3364	4	10.08	8.54E+03	4	2010	17100402.	1	58	79		
30	127	Q9UHG2|I	A.DHDVGSELPPEGVLGALLRV.K	Y	47.82	2072.09	20	2.7	691.7058	3	18.84	3.02E+04	5	3830	17100403.	2	223	242		
23	161	P02652|A	Y.FVELGTQPATQ	Y	27.77	1189.598	11	1.4	595.8071	2	7.21	4.95E+03	4	1358	17100402.	2	90	100		
23	161	P02652|A	F.LSYFVELGTQPATQ	Y	25.6	1552.777	14	1.1	777.3968	2	12.41	2.02E+04	5	2525	17100403.	1	87	100		
23	161	P02652|A	F.VELGTQPATQ	Y	21.68	1042.529	10	1.5	522.2728	2	3.7	4.19E+03	4	620	17100402.	1	91	100		
24	99	P05060|S	R.Q(-17.03)YDRVAQLDQLL.H	Y	28.26	1443.736	12	3.5	722.8777	2	14.91	1.42E+04	5	3044	17100403.	2	600	611	Pyro-glu from Q	Q1:Pyro-glu from Q:1000.00
26	157	P08697|A	A.MEPLGRQLTSGP.N	Y	30.27	1284.65	12	1.5	643.3331	2	7.11	1.52E+04	4	1333	17100402.	2	28	39		
26	157	P08697|A	M.EPLGRQLTSGP.N	Y	25.26	1153.609	11	0.9	577.8124	2	5.02	5.62E+04	4	880	17100402.	2	29	39		
27	158	P81172|H	G.SVFPQQTGQLAELQPQDRAGARAS.W	Y	29.52	2554.289	24	0.2	852.4371	3	8.9	2.44E+04	4	1738	17100402.	2	25	48		
27	158	P81172|H	G.SVFPQQTGQLAELQPQDRAGARASWMPM.F	Y	21.41	3099.502	28	5.5	1034.18	3	13.43	2.22E+04	4	2735	17100402.	1	25	52		
38	121	P02656|A	A.SEAEDASLLSFMQGYMKHA.T	Y	38.88	2113.945	19	2.9	705.6576	3	15.37	4.47E+04	5	3142	17100403.	1	21	39		
29	124	tr|H0Y300	L.GAVIALLLWGQ.L	Y	27.69	1139.67	11	4	570.8447	2	19.66	6.00E+04	4	3984	17100402.	1	5	15		
65	164	tr|A0A0C4	R.EAPYGAPRFDMPDFEDDGGPYGESEAPAPPGPGTRWPYRSRDT.R	Y	26.74	4710.078	43	1.2	943.024	5	12.08	2.87E+04	4	2450	17100402.	1	1426	1468		
50	170	tr|E5RG36	G.DQTVSDNELQEMSNQGSKYVNKEIQNAVNGVKQI.K	Y	24.06	3806.838	34	3.1	952.7197	4	14.51	6.53E+04	4	2960	17100402.	1	23	56		
66	184	P18206|V	L.APPKPPLPEGEVPPPRPPPPEEKDEEFPEQKAGEVIN.Q	Y	22.38	4037.042	37	1.2	808.4166	5	9.2	2.41E+04	4	1809	17100402.	1	859	895		
